# Transcriptional signature in microglia associated with Aβ plaque phagocytosis

**DOI:** 10.1038/s41467-021-23111-1

**Published:** 2021-05-21

**Authors:** Alexandra Grubman, Xin Yi Choo, Gabriel Chew, John F. Ouyang, Guizhi Sun, Nathan P. Croft, Fernando J. Rossello, Rebecca Simmons, Sam Buckberry, Dulce Vargas Landin, Jahnvi Pflueger, Teresa H. Vandekolk, Zehra Abay, Yichen Zhou, Xiaodong Liu, Joseph Chen, Michael Larcombe, John M. Haynes, Catriona McLean, Sarah Williams, Siew Yeen Chai, Trevor Wilson, Ryan Lister, Colin W. Pouton, Anthony W. Purcell, Owen J. L. Rackham, Enrico Petretto, Jose M. Polo

**Affiliations:** 1 Department of Anatomy and Developmental Biology, Monash University, Clayton, VIC Australia; 2 Development and Stem Cells Program, Monash Biomedicine Discovery Institute, Clayton, VIC Australia; 3 Australian Regenerative Medicine Institute, Monash University, Clayton, VIC Australia; 4 Department of Pathology, The University of Melbourne, Parkville, VIC Australia; 5 Program in Cardiovascular and Metabolic Disorders, Duke-National University of Singapore Medical School, 169857 Singapore, Singapore; 6 Department of Biochemistry and Molecular Biology, Monash University, Clayton, VIC Australia; 7 Infection and Immunity Program, Biomedicine Discovery Institute, Monash University, Clayton, VIC Australia; 8 ARC Center of Excellence in Plant Energy Biology, The University of Western Australia, Perth, WA Australia; 9 The Harry Perkins Institute of Medical Research, Perth, WA Australia; 10 Monash Institute of Pharmaceutical Sciences, Monash University (Parkville Campus), Parkville, VIC Australia; 11 Department of Microbiology, Monash University, Clayton, VIC Australia; 12 Victorian Brain Bank, Parkville, VIC Australia; 13 Department of Physiology, Monash University, Clayton, VIC Australia; 14 MHTP Medical Genomics Facility, Clayton, VIC Australia

**Keywords:** Gene expression, Alzheimer's disease, Microglia

## Abstract

The role of microglia cells in Alzheimer’s disease (AD) is well recognized, however their molecular and functional diversity remain unclear. Here, we isolated amyloid plaque-containing (using labelling with methoxy-XO4, XO4^+^) and non-containing (XO4^−^) microglia from an AD mouse model. Transcriptomics analysis identified different transcriptional trajectories in ageing and AD mice. XO4^+^ microglial transcriptomes demonstrated dysregulated expression of genes associated with late onset AD. We further showed that the transcriptional program associated with XO4^+^ microglia from mice is present in a subset of human microglia isolated from brains of individuals with AD. XO4^−^ microglia displayed transcriptional signatures associated with accelerated ageing and contained more intracellular post-synaptic material than XO4^+^ microglia, despite reduced active synaptosome phagocytosis. We identified HIF1α as potentially regulating synaptosome phagocytosis in vitro using primary human microglia, and BV2 mouse microglial cells. Together, these findings provide insight into molecular mechanisms underpinning the functional diversity of microglia in AD.

## Abstract (web summary)

Microglia associated with Aβ plaques may have a distinct transcriptional signature compared to those in plaque-free areas of the brain in Alzheimer’s disease (AD) models. Here the authors show that amyloid plaque phagocytosis is associated with a specific microglia transcriptional signature in a mouse model of AD.

## Introduction

Microglia are specialist immune sentinel cells in the brain that respond to stranger or danger signals, remove cellular and extracellular debris, and regulate synaptic plasticity, maturation and removal^1–3^. Thus, microglial function is vital to physiological processes in the brain. AD is a progressive neurodegenerative condition, with no effective treatment options. Synapse loss, which occurs in cortical and hippocampal regions, most strongly correlates with cognitive dysfunction in AD^4^, and is accompanied by extracellular amyloid beta (Aβ) plaques and intraneuronal neurofibrillary tau tangles^5^. The role of microglia in AD has been highlighted by several unbiased data-driven functional genomics studies^6–11^. Indeed, almost all of the risk loci implicated in genome-wide association studies (GWAS) of the more common late-onset AD (LOAD; > 95% cases) are associated with genes that have been reported to be microglia specific, or highly expressed in microglia^12^. Additionally, recent reports in mouse models have found that microglia obtained from areas rich in plaques have a different transcriptional signature from microglia from plaque-free areas^13,14^. However, the origin of these microglia, their gene expression signatures and functions remain unknown. Furthermore, knockouts of microglial receptor genes on an AD genetic background have yielded both protective and exacerbated phenotypes, often at different disease stages^15–21^. Coupled with the increasingly recognized spatio-temporal diversity of microglia^22–24^, these reports highlight the dynamic nature and complexity of microglial responses, which might be explained by the presence of multiple microglial subpopulations that may differentially affect disease course.

Here we show using an aggressive plaque-depositing AD mouse model that differences in amyloid plaque phagocytosis are directly associated, molecularly and functionally, with specific microglia phenotypes in AD.

## Results

### Methoxy-XO4 labels molecularly distinct plaque-associated microglia populations

**Fig. 1:**
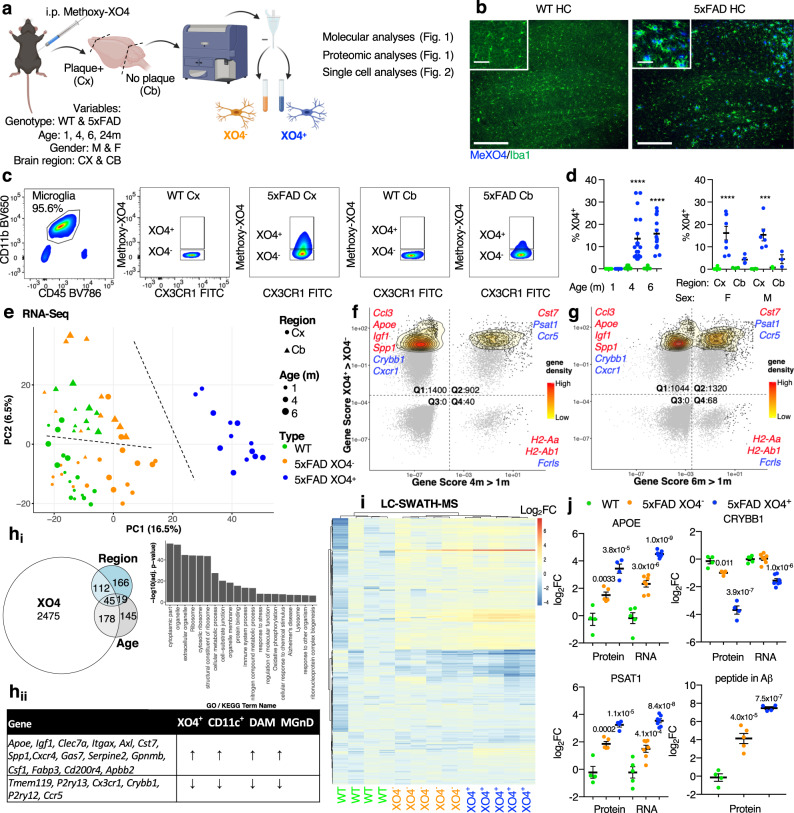
Methoxy-XO4 labels a molecularly distinct plaque-phagocytic population in 5xFAD mice. **a** Schematic of the methodology employed in this study, created with BioRender.com. M, male, F, female, WT, wild-type, Cx, cortex and subcortical regions, Cb, cerebellum. **b** Representative immunofluorescence image of the hippocampus (HC) of WT and 5xFAD mice injected with methoxy-XO4 and stained with Iba1 (AlexaFluor 488, *n* = 6 animals per genotype), scale bar = 250 μm, inset 50 μm. **c** Representative FACS plot showing that XO4^+^ microglia are present in 6 m 5xFAD plaque-affected regions (top panels). **d** Left, the percentage of XO4^+^ microglia isolated from plaque-affected regions in 1, 4 and 6 m old WT (m, month) and 5xFAD mice (from *n* = 6 animals per genotype at 1 m; 4 m WT, *n* = 19 animals; 4 m 5xFAD, *n* = 22; 6 m WT, *n* = 14; 6 m 5xFAD *n* = 14) and right, the percentage of XO4^+^ microglia isolated from plaque-affected and non-affected regions in 6 m old male and female WT and 5xFAD mice (F, Cx, *n* = 8 per genotype; M, Cx, *n* = 6 per genotype; F, Cb, *n* = 4 per genotype; M, Cb, *n* = 3 per genotype), expressed as mean ± SEM, ***p = 0.003 and ****p = 4.6 × 10^−5^ for 4 m, *p* = 9 × 10^−6^ for 6 m, and *p* = 5.2 × 10^−5^ for F Cx vs Cb by Kruskal-Wallis and Dunn’s multiple comparison tests. **e** PCA of bulk RNA-seq. Cx, Cortex; Cb, Cerebellum. **f**, **g** Gene cytometry plots showing DEGs between XO4^+^ and XO4^−^ microglia and/or DEGs expressed between old (4, 6 m) and young (1 m) microglia. Gene scores are calculated as the product of the LFC and –log_10_(FDR). Example genes in each quadrant are labelled in red (upregulated over time or phagocytosis) or blue (downregulated). Gene density low = 0, high = 0.2. **h**_**i**_ Venn diagram showing the overlap between genes whose expression levels could be explained by the age, region and XO4 covariate as well as GO and KEGG terms associated with XO4 covariate genes. **h**_**ii**_ Table showing the 21 core microglial neurodegeneration signature genes and their direction of differential expression in DAM^28^, CD11c^+^^29^, MGnD^30^ and XO4^+^ microglia. **i** Heatmap of targeted LC-SWATH-MS analysis of detected peptides within DEGs in biological replicates of WT (green, *n* = 4 animals), XO4^−^ 5xFAD (orange, *n* = 5) and XO4^+^ 5xFAD (blue, *n* = 4) microglia. Colour scale represents log_2_-transformed normalized fold changes compared to WT microglia. clustering method = ward.D2, distance = maximum. **j** Comparison of RNA and protein expression for selected genes, and quantitation of a tryptic peptide in Aβ in microglia. Data are expressed as mean ± SEM LFC compared to WT microglia, normalized relative to peptides in Supplementary Data 4. *p*-Values were calculated by one-way ANOVA using Holm-Sidak’s multiple comparison test. Data are from WT (*n* = 4 animals), XO4^−^ 5xFAD (*n* = 5), XO4^+^ 5xFAD (*n* = 4) for protein analyses; WT (*n* = 5), XO4^−^ 5xFAD (*n* = 7), XO4^+^ 5xFAD (*n* = 7) for RNA analyses.

To understand the spatio-temporal and functional differences between plaque-phagocytic and non-phagocytic microglia in AD, we took advantage of in vivo Aβ plaque labelling using a fluorescent Congo-red derivative, methoxy-XO4^25^, which co-localized with CD68^+^ phagosomes in plaque-associated Iba1^+^ microglia (Fig. 1a, b and Supplementary Fig. 1a–c). We found that 13.5% and 15.8% of cerebral microglia were actively amyloid-phagocytosing (XO4^+^) specifically in 4 m and 6 m old 5xFAD mice, respectively (Fig. 1c, d). Only 4.35% of cerebellar microglia in 5xFAD mice were XO4^+^, in accordance with the relative resistance of this region to pathology in mouse AD models and AD patients^26^.

Next, we transcriptionally profiled XO4^+^ and XO4^−^ microglia during disease progression in 5xFAD mice^27^. XO4^+^ microglia were transcriptionally most different from remaining microglia by principal component analysis (PCA, Fig. 1e). The first PC evidenced the progression from wild-type (WT) to XO4^+^ microglia, whereas the second PC separated cerebellar from cerebral microglia (Fig. 1e and Supplementary Fig. 1d). The factor explaining the greatest variance in gene expression was plaque phagocytosis (i.e., XO4^+^/XO4^−^, Supplementary Fig. 2a). To investigate whether plaque phagocytosis is affected by age, we created gene cytometry plots, which show the distribution of differentially expressed genes (DEGs) by their significance score (i.e., false discovery rate (FDR)-weighted log fold change (LFC)) for both age and XO4^+^/XO4^−^ factors (Supplementary Fig. 2b). We identified gene sets that are associated with either age, plaque internalization or both (Fig. 1f, g and volcano plots in Supplementary Fig. 2c–e). Most DEGs (black points and contour plot) were associated with XO4^+^ microglia (Fig. 1f, Q1 and Q2) which was amplified by ageing, as 39% (902/2302 genes) of XO4^+^-associated genes were also associated with age at 4 m (m, month; Fig. 1f, Q2), rising to 56% (1320/2364 genes) by 6 m (Fig. 1g, Q2; and Supplementary Fig. 2f).

Overall, out of 2810 XO4^+^ genes we identified 2475 genes associated only with the plaque-phagocytic XO4^+^ state, which were enriched for the Gene Ontology (GO) terms’ ribosome, oxidative phosphorylation and phagolysosome pathways (Fig. 1h_i_ and Supplementary Data 1). Among the most upregulated genes in XO4^+^, microglia were the two most highly penetrant LOAD risk factor genes, the receptor *Trem2* and its ligand *Apoe*^28^, as well as genes encoding their interacting partners, *Tyrobp*^10^ and *Lpl, Ldlr, Lrpap1* (reviewed in^29^), respectively, suggesting a link between phagocytosis and cholesterol transport pathways and the XO4^+^ phenotype in AD. A significant proportion (63%, *p* = 6.1 × 10^−11^, hypergeometric test) of microglial sensome genes^30^ were DEGs in XO4^+^ microglia (Supplementary Fig. 3a), including c-lectins (*Clec4a2, Clec4a3*) and CD markers (*Cd33, Cd68*). The XO4^+^-associated gene expression signature identified here partially overlaps with other microglia signatures obtained from aged, APP/PS1, 5xFAD or tau model mice^13,14,31–33^ (Supplementary Fig. 3b–e and Supplementary Data 2). Twenty one core genes associated with XO4^+^/XO4^−^ phenotype were identified as altered in several studies of neurodegenerative disease-associated microglia (DAM)^13,14,32^ (Fig. 1h_ii_). However, despite overlap of KEGG pathways with previously reported microglial gene expression signatures, our analysis showed differences between each signature (Supplementary Fig. 3c, e). First, XO4^+^-associated microglia genes were more significantly enriched for additional functions including HIF1 signalling pathway, steroid biosynthesis, mitophagy and protein processing in endoplasmic reticulum, and highly enriched for neurodegenerative signatures including Alzheimer’s, Parkinson’s and Huntington’s disease genes. Second, our analysis identified 2031 (out of 2810, 72.3%) genes associated with XO4^+^ microglia that were not reported in previous RNA-sequencing studies of microglia in neurodegeneration^13,14,33^ (Supplementary Fig. 3b and Supplementary Data 2). These newly identified genes were highly enriched for Alzheimer’s disease, oxidative phosphorylation, cell cycle and HIF1 signalling pathway (Supplementary Fig. 3d). Our detection of this previously unappreciated gene expression signature associated with XO4^+^ microglia might be due to several factors. First, we specifically profiled the microglia (CD11b^+^CD45^lo^CX3CR1^+^) population in AD that have phagocytosed plaques (XO4^+^), rather than using more heterogeneous approaches (CD11c^+^ or Clec7a^+^) which did not include a marker for plaque per se. Second, we obtained a more homogenous dataset than either bulk APP/PS1 or CD11c^+^ microglia (which contains both plaque-associated and -distal microglia^13^), and we sequenced to a greater depth than the study defining DAM^13^. Importantly, our study describes the transcriptional signature of a functionally distinct microglial subtype defined by active fibrillar Aβ (fAβ) phagocytosis.

To determine whether the observed transcriptional signatures correspond to changes in the proteome of XO4^+^ microglia, we performed targeted liquid-chromatography sequential window acquisition of all theoretical spectra mass spectrometry (LC-SWATH-MS)^34^ analysis (Fig. 1i). Out of 304 proteins corresponding to the detected DEGs, downregulation of 19 genes in XO4^+^ microglia was mirrored at the protein level, including for DOCK10, CRYBB1, PLXNA1 and FGD2 (Supplementary Fig. 4 and Supplementary Data 3). We also detected significantly elevated concentrations of peptides corresponding to 99 DEGs upregulated in XO4^+^ microglia, including numerous ribosomal RPS and RPL family proteins, lysosomal proteins (e.g., CD68, CTSA, CTSB, CTSD, CTSZ, RAB7), HIF1A target proteins (e.g., ALDOA, GAPDH, LDHA, PKM), lipid-associated proteins (LGALS3BP, LRPAP1, APOE) and proteins specific to XO4^+^ microglia (e.g., ANXA5, RPL6L, PKM). Nonetheless, some transcripts induced in XO4^+^ microglia were repressed post-transcriptionally (e.g., SERPINE2, UQCRH and MYO5A). Importantly, in 5xFAD, but not in WT microglia, we detected a single peptide within the amyloid precursor protein (APP) sequence (LVFFAEDVGSNK), compared to multiple tryptic peptides arising from APP fragments detected within purified synaptosome fractions (Supplementary Data 4). LVFFAEDVGSNK is the only one of the tryptic peptides we detected within the APP sequence that is present within the Aβ sequence (Aβ_17–28_)^35^, suggesting this peptide arose from microglial phagocytosis of Aβ and not from microglial expression of APP or phagocytosis of APP. XO4^+^ microglia contained high levels of internalized Aβ (Fig. 1j). Surprisingly, XO4^−^ microglia also contained Aβ, albeit at ~10-fold lower levels than XO4^+^ microglia. This is consistent with low levels of non-fibrillar (i.e., oligomeric) Aβ phagocytosis by XO4^−^ microglia, although this could also be explained by few contaminating XO4^+^ cells containing partially digested Aβ fragments and thus not detected by methoxy-XO4 staining. Our data therefore suggest that XO4^+^ are distinct from homeostatic and XO4^−^ microglia and are directly associated with high levels of internalized fibrillar Aβ.

### Two distinct molecular processes identified in microglial alterations in AD

**Fig. 2:**
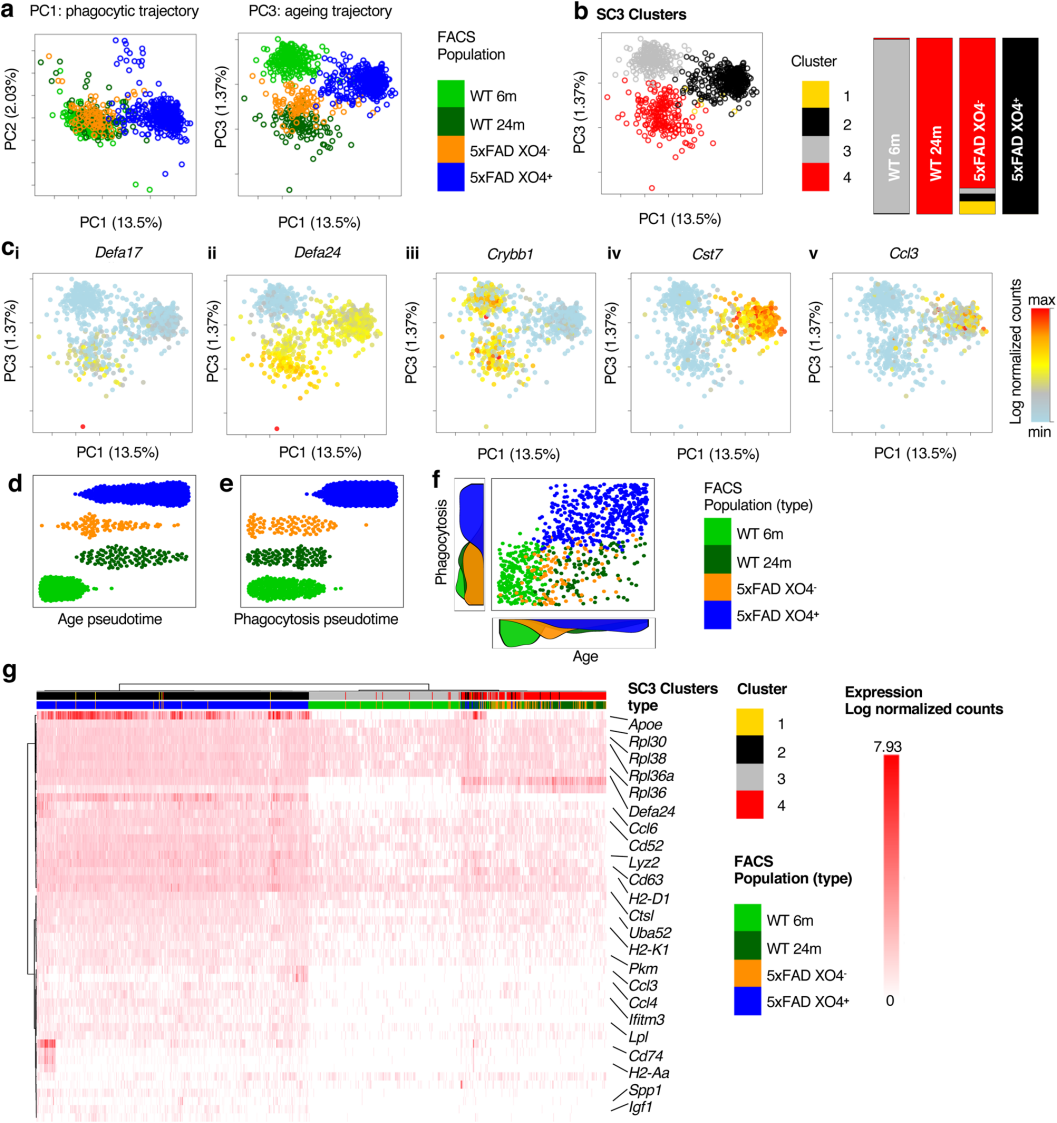
Single-cell sequencing identifies an ageing profile in 5xFAD XO4^−^ microglia. **a** PCA of 893 single cells (6 m WT = 243 cells, 24 m WT = 121 cells, 6 m 5xFAD XO4^**−**^ = 95 cells, 6 m 5xFAD XO4^+^ = 434 cells; m, month) and 1671 feature genes showing the distribution of cells from each FACS-sorted sample. PC, principal component. **b** PCA plot of single microglia coloured by single cell consensus (SC3) clusters and composition of automated clusters as a percentage of sequenced FACS-sorted cell populations. **c** PCA plots for single microglia coloured by expression of selected ageing microglia genes (i-ii), homeostatic (iii) and signature genes associated with XO4^+^ microglia (iv-v). min = 0 for all genes, *Defa17* max = 4.77*, Defa24* max = 7.41*, Crybb1* max = 4.13*, Cst7* max = 5.47*, Ccl3* max = 4.89. **d**, **e** Diffusion maps pseudotime analysis of microglial populations ordered by their expression of (**d**) ageing DEGs (24 m WT vs 6 m WT, 42 DEGs) or (**e**) phagocytic DEGs (6 m 5xFAD XO4^+^ vs 6 m 5xFAD XO4^−^, 474 DEGs). **f** Scatter plot showing the relationship between ageing and phagocytosing pseudotime in individual cells, and the density of cells at each point during the ageing (bottom) and phagocytosing (left) trajectories. **g** Hierarchical clustering and heatmap showing expression of the top 50 DEGs across the 4 SC3 clusters.

Recent reports highlight subtle transcriptional differences between individual microglial cells, even those residing within the same anatomical regions^36,37^. Exploratory viSNE analysis of FACS datasets showed heterogeneity inside the microglia cell populations (Supplementary Fig. 5a–c), which we further investigated by single-cell RNA-sequencing (scRNA-seq) using the 10X Genomics Chromium system. Importantly, in order to control for potential confounding effects due to cell sorting^31,38^, we checked and confirmed these do not affect our detection of gene expression signatures associated with XO4^+^/XO4^−^ microglia (Supplementary Fig. 5d, e). Aging is the most important risk factor for LOAD, and microglia are known to express an altered ageing gene signature^22,39^. We examined whether microglial subpopulations in 5xFAD mice adopted an aging phenotype by including 893 FACS-sorted microglia from WT adult (6 m) and WT old mice (24 m) as well as both XO4^−^ and XO4^+^ populations from a 6 m old 5xFAD mouse (Fig. 2a). Similar to our bulk analyses of XO4^+/−^ microglia, PC1 was dominated by the shift to a plaque-phagocytosing phenotype. We detected a substantial overlap of 344 DEGs (64.2% of single-cell gene expression signature associated with XO4^+^ microglia, *p* = 3.35 × 10^−34^, hypergeometric test) between the single-cell (536 DEGs) and bulk gene expression signatures associated with XO4^+^ microglia (2810 DEGs), most strongly enriched for ribosomal and protein synthesis-related processes. The overlapping gene set included key AD-related genes such as *Cst7, Apoe* and *Tyrobp*, and *Trem2* and was substantially enriched for *Hif1a*-related genes such as *Gapdh, Igf1, Aldoa, Pkm* and *Ldha* (Supplementary Fig. 5f, g).

PC3 separated 6 m from aged WT cells, while 5xFAD XO4^−^ cells were also shifted in the direction of aged WT microglia (Fig. 2a). Single-cell consensus clustering (SC3)^40^ separated microglia into four clusters, whereby 99.8% of 5xFAD XO4^+^ cells and 99.2% of WT 6 m microglia were entirely contained within Clusters 2 and 3, respectively (Fig. 2b). 5xFAD 6 m XO4^−^ microglia and aged WT microglia clustered together (i.e., have similar transcriptional profiles), suggesting that the XO4^−^ population may represent an accelerated aging phenotype in AD mice. The ageing signature was enriched in α-defensin genes (Fig. 2c_i-ii_), antimicrobial peptides which mobilize immune cells and enhance phagocytosis in the periphery^41^, although their role in the brain has not been described previously. Consistent with our bulk RNA-seq data (Fig. 1e, h), we observed loss of homeostatic genes (e.g., *Crybb1*, Fig. 2c_iii_) and upregulated genes associated with XO4^+^ microglia (e.g., *Cst7, Ccl3*, Fig. 2c_iv-v_) in XO4^+^ microglia. Comparing the single-cell signature associated with XO4^+^ microglia to previously described mouse microglia gene signatures in AD (Supplementary Fig. 6a–e) revealed a substantial number of XO4^+^-specific DEGs, which are enriched uniquely for inflammatory-related processes including response to interferon-gamma, T cell homeostasis and regulation of mononuclear cell migration. Some XO4^+^-specific DEGs include phagocytosis and immune process-related genes like *Il17ra, Lst1, Crip1*, and also including c-lectins and galectins, *Clec4a3, Clec4a2, Lgals2, Lgals3* and *Lgals4*. Therefore, despite the overlap between the functional phagocytic gene expression signature associated with XO4^+^ microglia and other transcriptionally defined mouse microglia signatures (i.e., DAM or Trem2-associated), we report XO4^+^-associated DEGs specifically reflecting immune, metabolic, and/or phagocytosis-related processes.

To further elucidate the molecular microglial trajectories in 5xFAD mice, we used the diffusion maps algorithm Destiny^42^ to order cells in aging pseudotime, defined by the expression of the 42 ageing-specific DEGs (FDR < 0.05, top 20 DEGs for ageing are shown in Supplementary Fig. 7a, full list in Supplementary Data 5) between 6 m and 24 m WT microglia (Fig. 2d). Despite heterogeneity in the pseudoage of individual cells within 5xFAD microglial populations, both the XO4^−^ and XO4^+^ groups shifted along the aging trajectory, suggesting gradual acquisition of the ageing signature. Conversely, cells ordered by their phagocytic pseudotime, using 474 phagocytosis-specific DEGs (FDR < 0.05, top 20 DEGs for phagocytosis are shown in Supplementary Fig. 7b, full list in Supplementary Data 5) between XO4^+^ and XO4^−^ AD microglia, switched from a non-phagocytic to a phagocytic gene expression signature with few cells exhibiting intermediate signatures (Fig. 2e). Although the molecular signatures associated with XO4^+^ and XO4^−^ microglia are distinct, the pseudoage of individual microglia lies on a continuum within each 5xFAD population that is controlled by an independent component of aging, unrelated to phagocytosis (Fig. 2f), and may reflect differences in cellular age. The top 50 most variable genes included microglial identity genes (*Crybb1, Alox5ap, Maf*), *Hif1a* and its target genes (*Hif1a, Igf1, Spp1, Pkm, Gapdh*), α-defensin genes (*Defa20, Defa21, Defa24, Gm15284*), chemokines (*Ccl3, Ccl4, Ccl6*) and lysosomal genes (*Lyz2, Cst7, Ctsa*), and clustered samples according to both age and phagocytic phenotype (Fig. 2g). The two separate components corresponding to an ageing and phagocytic trajectory were independently reconstructed using Slingshot^43^ (see “Methods”), a minimum spanning tree algorithm for lineage construction (Supplementary Fig. 8a, b). Moreover, microglia from 9 and 12 m old CX3CR1^GFP^ (WT for the AD mutations) animals showed intermediate ageing pseudotime (Supplementary Fig. 8c). Furthermore, neither Stage I nor Stage II DAM genes^13^ are dysregulated in XO4^−^ as compared with WT cells (Supplementary Fig. 8d, e). This suggests that XO4^−^ microglia are on an independent trajectory and are not necessary cellular intermediates poised to become XO4^+^ cells.

### The molecular signature associated with XO4^+^ microglia can be induced by phagocytosis of amyloid plaques

**Fig. 3:**
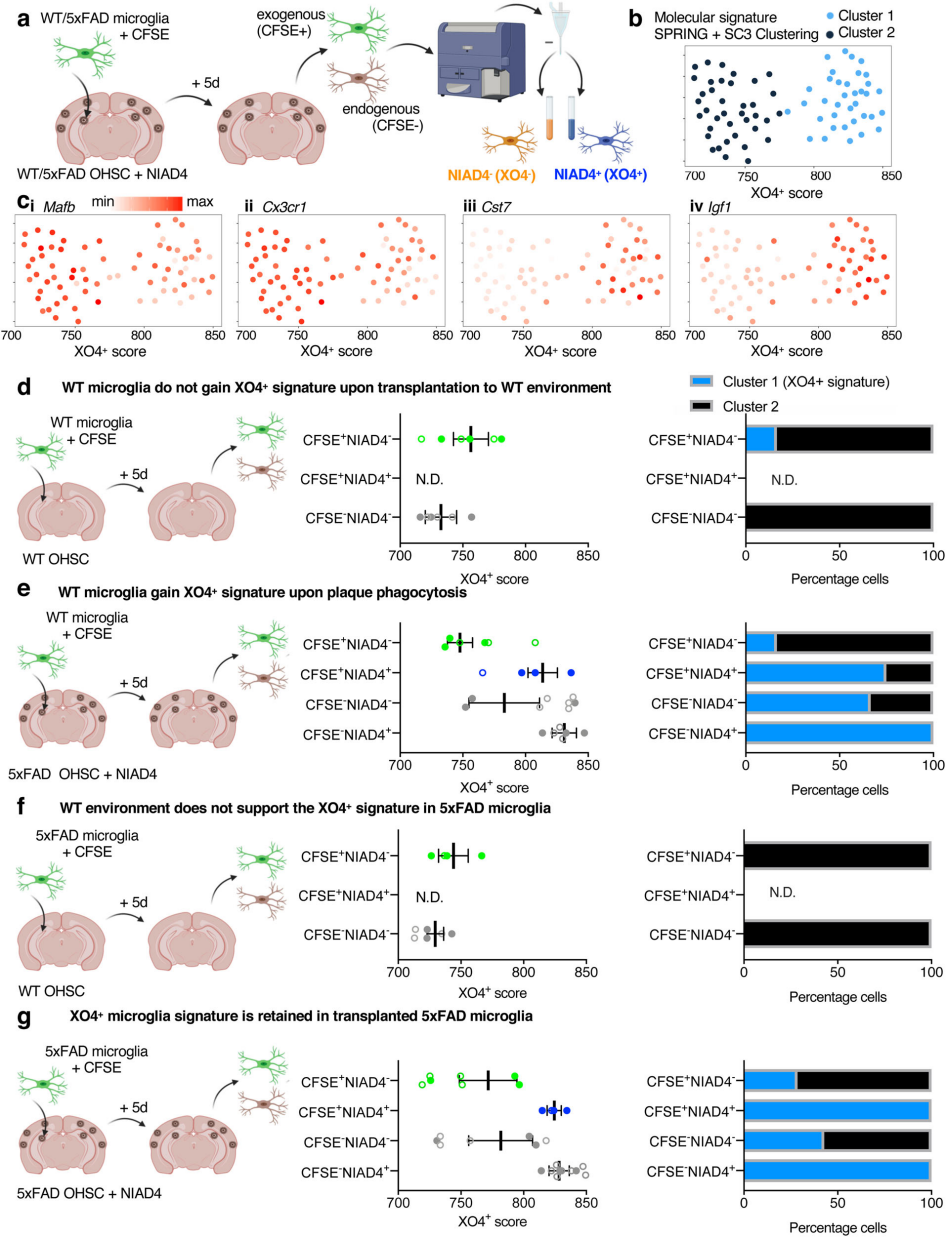
The gene expression signature associated with XO4^+^ microglia is reversible and is acquired through phagocytosis of amyloid plaques. **a** Schematic representing the experimental design involving addition of 2 × 10^4^ microglia to NIAD4-stained organotypic hippocampal slice cultures (OHSCs), followed by FACS isolation of carboxyfluorescein succinimidyl ester (CFSE)-labelled replenished and CFSE^-^ endogenous microglia that differentially phagocytose endogenous NIAD4-labelled plaques after 5 days co-culture with wild-type (WT) or 5xFAD OHSCs, created with BioRender.com. **b**, **c** *k*-nearest-neighbour (kNN) graph rendered using a force-directed layout (SPRING)^142^, coloured by single cell consensus (SC3) cluster (**b**), and log_2_-transformed ΔCt values of selected DEGs (**c**). Each dot represents 10 sorted cells, and data are from (**d**) *n* = 120 cells, (**e**) *n* = 240 cells, (**f**) *n* = 110 cells, (**g**) *n* = 280 cells sorted during 3 independent experiments. Replicates from independent experiments are closed circles, technical replicates are open circles. The XO4^+^ score is defined as the *x*-axis position of each sorted population on the kNN graph. The colour scales are log_2_(ΔCt), *Mafb* min ΔCt = 0.0003, max ΔCt = 3.37; *Cx3cr1* min ΔCt = 0.0001, max ΔCt = 9.69; *Cst7* min ΔCt = 0.0002, max ΔCt = 4.56; *Igf1* min ΔCt = 0.0001, max ΔCt = 1.07. **d**–**g** Experimental schematic, XO4^+^ score and proportion Cluster 1 and Cluster 2 membership of groups of exogenous and endogenous (**d**) WT microglia added into a WT OHSC, (**e**) WT microglia added into 5xFAD slices, recapitulating the gene expression signature associated with XO4^+^ microglia upon plaque phagocytosis. **f** XO4^+^ phenotype is stable in exogenous CFSE^+^NIAD4^+^ 5xFAD microglia recovered from 5xFAD slices, but (**g**) is lost in CFSE^+^ 5xFAD microglia recovered from WT slices. N.D., not detected. Data are presented as mean ± SEM.

We next asked whether the XO4^+^-associated transcriptional program is a consequence of the microenvironment in AD brains per se, or if plaque phagocytosis predates and is required for this molecular switch. Thus, to determine whether and how any microglia could activate an XO4^+^-associated gene expression signature, we added exogenous CFSE-labelled WT microglia onto ex vivo organotypic hippocampal slice cultures (OHSCs) from 6 m 5xFAD mice (Fig. 3a). To ensure that the gene expression signature associated with XO4^+^ microglia occurred independently of the methoxy-XO4 dye, we instead labelled OHSCs with NIAD4^44^, an alternative fluorescent Aβ-binding dye. To establish that (1) healthy and (2) plaque-associated microglial signatures can be detected using our system, we also cultured (1) WT microglia with WT OHSCs and (2) 5xFAD microglia with 5xFAD OHSCs, respectively. Using FACS, we sorted groups of 10 endogenous (CSFE^−^) and exogenous (CFSE^+^) microglial cells that were plaque-positive (NIAD4^+^) or -negative (NIAD4^−^) for molecular profiling (Supplementary Fig. 9a). QPCR analysis followed by SC3 clustering of a panel of 42 homeostatic and XO4^+^-associated signature genes identified two main clusters of microglia cells (Fig. 3b and Supplementary Data 6). Cluster 1 represents the XO4^+^-associated molecular signature, with numerous homeostatic microglia signature genes downregulated (e.g., *Cx3cr1, Maf, P2ry12;* Fig. 3c_i-ii_ and Supplementary Fig. 9b), and activated expression of genes associated with XO4^+^ microglia (e.g., *Cst7, Igf1*, *Apoe, Spp1, Trem2, Lgals3;* Fig. 3c_iii-iv_ and Supplementary Fig. 9c). Conversely, Cluster 2 is characterized by high expression of homeostatic microglial genes and low expression of genes associated with XO4^+^ microglia. WT microglia generally retained a Cluster 2 signature when transplanted onto a WT ex vivo brain slice (Fig. 3d). Similarly, most WT microglia that had not phagocytosed plaques (CFSE^+^NIAD4^−^) retained their homeostatic Cluster 2 signature even in a 5xFAD ex vivo brain slice (Fig. 3e). However, most cells sorted from WT mice that were actively phagocytosing the plaques in 5xFAD OHSCs (CFSE^+^NIAD4^+^) acquired the Cluster 1 XO4^+^-associated gene expression signature (Fig. 3e). These data suggest that microglial plaque phagocytosis, and not exposure to an ex vivo AD-like brain microenvironment, is the main trigger for conversion to the XO4^+^ state and demonstrate that WT microglia are capable of acquiring the gene expression signature typical of XO4^+^ microglia.

Our data suggest that the gene expression signature associated with XO4^+^ microglia is reversed in a WT brain slice (Fig. 3f), as over half of exogenous CFSE^+^ 5xFAD cells isolated from 5xFAD OHSCs molecularly resembled Cluster 1/XO4^+^ microglia (Fig. 3g). This suggests that cell survival is not impacted, whereas all 5xFAD CFSE^+^ cells recovered from WT OHSCs were in the homeostatic Cluster 2 (Fig. 3f). An alternative possibility is that the signature is transient and reverts upon digestion of internalized fibrillar Aβ. However, our results do not uncover whether the microglia that were originally XO4^+^ in vivo died within the slices, or degraded phagocytosed fAβ. Collectively, our data show that the XO4^+^ transcriptional program is activated by plaque phagocytosis and does not persist after exposure to a healthy brain microenvironment ex vivo.

### *Hif1a* is associated with XO4^+^ microglia-induced phagocytosis of synaptosomes in vitro

**Fig. 4:**
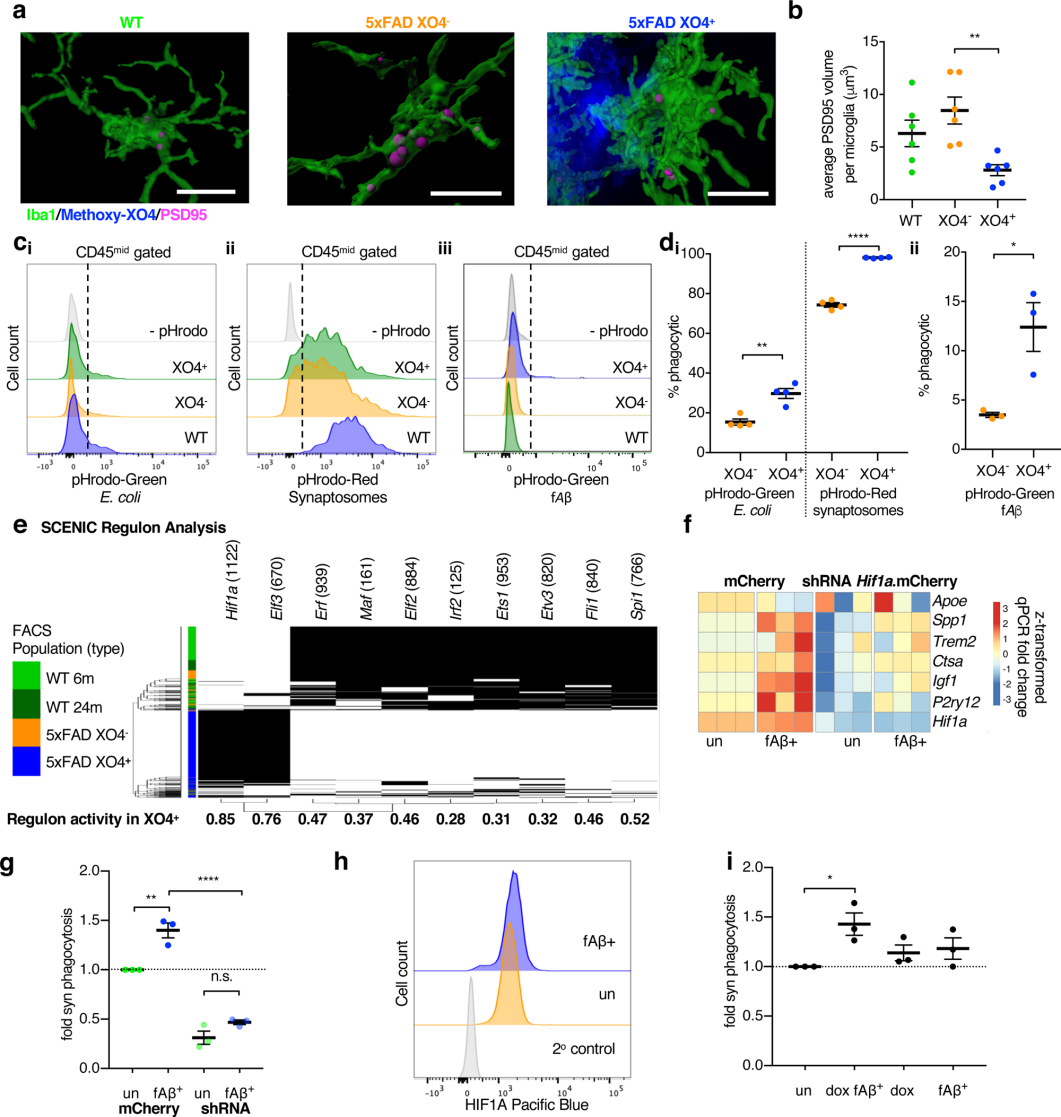
5xFAD XO4^+^ microglia contain less post-synaptic material than 5xFAD XO4^−^ microglia in the dentate gyrus. **a** Representative 3D reconstructions of confocal *z*-stacks showing PSD95 internalized within WT, 5xFAD XO4^−^ or 5xFAD XO4^+^ microglia cells (scale bars = 15 μm). **b** PSD95 within microglia quantified as the average volume of phagocytosed PSD95 volume per microglia volume in each dentate gyrus section (*n* = 6 *z*-stacks per condition; *p = 0.0057, using one-way ANOVA and Tukey’s multiple comparison test). All data are from *n* = 3 WT and *n* = 6 5xFAD animals and is presented as mean ± SEM per individual section. **c** Functional analysis of ex vivo mouse microglia phagocytosis following 1 h incubation with (**c**_**i**_) pHrodo-green-labelled *E. coli*, (**c**_**ii**_) pHrodo-red-labelled synaptosomes or (**c**_**iii**_) pHrodo-green-labelled fAβ by FACS. Each population is gated based on XO4^+^ signal and compared to controls not incubated with pHrodo particles. **d**_**i**_ Quantitation of the percentage of XO4^+^ and XO4^−^ microglia that phagocytose pHrodo-red-labelled synaptosomes or pHrodo-green-labelled *E. coli* (comparing XO4^−^ and XO4^+^ microglia from *n* = 4 animals), or **d**_**ii**_ pHrodo-green-labelled fAβ (comparing XO4^−^ and XO4^+^ microglia from *n* = 3 animals). Data in (**d**) are presented as mean ± SEM. ^*^p = 0.0233, ^**^p = 0.0027 and ^****^p = 9.2 × 10^−7^ by paired 2-tailed *t*-test. **e** SCENIC regulon analysis showing that *Hif1a* and *Elf3* are predicted to control the XO4^+^ gene regulatory network. The number of genes in each regulon is shown in parentheses. **f**, **g** BV2 cells were stably transduced with mCherry or mCherry.*shHif1a* lentivirus and treated with DMSO or AF488-labelled fAβ for 24 h, then blue-labelled synaptosomes for 1.5 h. mCherry^+^ cells were FACS sorted for AF488-fAβ. **f** Normalized heatmap of gene expression, measured by qPCR, of signature genes associated with XO4^+^ microglia in fAβ^+^ and non-treated (un) BV2 cells with or without *shHif1a*, including *Hif1a* regulon genes (*Igf1, Spp1, Ctsa, Hif1a*) and genes not part of the *Hif1a* regulon (*Apoe, Trem2, P2ry12*). Data are expressed as fold change relative to non-treated mCherry transduced cells, based on ΔCt values relative to *Actb*. The data are from 3 independent experiments. **g** The proportion of cells that are highly phagocytic for blue-bead-labelled synaptosomes. Data are expressed as fold change in % phagocytosis relative to non-treated mCherry transduced cells (mean ± SEM). The data are from 3 independent experiments performed in triplicate. n.s., *p* = 0.22, ^**^p = 0.0026, ^****^p = 6.0 × 10^−6^ by two-way ANOVA using Tukey’s multiple comparison test. **h** Histograms showing fluorescence intensity of HIF1A intracellular staining in AF488-fAβ^+^ and non-treated BV2 cells. Secondary antibody control cells are stained with Pacific-blue-labelled secondary antibodies alone. **i** The proportion of dox-treated (or not) and fAβ^+^ or non-treated (un) BV2 cells transduced with dox-inducible *Hif1a* expression constructs that are highly phagocytic for blue-bead-labelled synaptosomes. Data are expressed as fold change in % phagocytosis relative to non-treated mCherry transduced cells (mean ± SEM). The data are from 3 independent experiments performed in triplicate. ^*^p = 0.0253 by one-way ANOVA using Holm-Sidak’s multiple comparison test.

Aβ oligomers have been reported to induce microglia to aberrantly engulf synapses via dysregulated complement deposition^45^. Thus, we hypothesized that due to their transcriptional differences arising as a consequence of phagocytosis of plaques, additional microglial functions might be different between XO4^−^ and XO4^+^ states, for example, a differential capacity for engulfment of synaptic proteins. Quantification of the internalized post-synaptic marker, PSD95^46^, in individual microglia in the dentate gyrus of WT and 5xFAD mice (Fig. 4a and Supplementary Fig. 10a) showed an increase in steady-state internal PSD95 in XO4^−^ microglia compared to XO4^+^ microglia (Fig. 4b; *p* = 0.0057, one-way ANOVA and Tukey’s multiple comparison test).

However, functional phagocytosis assays tracing pHrodo-red-labelled synaptosomes on freshly isolated ex vivo microglia from 5xFAD mice by FACS revealed a higher rate of internalization by XO4^+^ microglia (Fig. 4c, d; *p* = 9.2 × 10^−7^, 2-tailed Student’s *t*-test). pHrodo-green-labelled *Escherichia coli* particles and pHrodo-green-labelled fibrillar Aβ (fAβ) were also more efficiently phagocytosed by XO4^+^ microglia compared to XO4^−^ (Fig. 4c, d; *p* = 0.0027 and *p* = 0.0233, respectively, 2-tailed Student’s *t*-test), suggesting that, at least at 6 m, XO4^+^ microglia are primed for increased phagocytic and degradative capacity, which is consistent with their increased gene and protein expression of lysosomal enzymes (i.e., Cathepsins CTSA, CTSB, CTSD, CTSZ, RAB7, Supplementary Data 3). We cannot rule out a possibility that over time and in the presence of chronic inflammation, digestion of fAβ (and/or synapses) may be impaired as described in response to fAβ in LPS-primed microglia^47^. Alternatively, fAβ clearance may also be impaired in vivo compared to PSD95 due to differences in substrate structure and/or high concentrations of fAβ in plaques. The reduced steady-state PSD95 detected within XO4^+^ microglia by immunofluorescence may be reflective of loss of synapses around plaques (Supplementary Fig. 10b) and in line with^48^.

To identify transcription factors (TFs) driving the gene expression signature associated with XO4^+^ microglia we used Single-Cell Regulatory Network Inference and Clustering (SCENIC)^49^. For each cell population, SCENIC defines its regulon (i.e., the TF and its putative targets) and infers the regulon’s activity. The higher the regulon’s activity, the greater the predicted TF-influence in the cell. The highest regulon’s activity in XO4^+^ cells was identified for *Hif1a* and *Elf3* TFs (Fig. 4e and Supplementary Data 7).

*Hif1a* was also identified as a driving TF in XO4^+^ microglia using pySCENIC^50^. Additionally, *Hif1a* was predicted for both 87.8% of XO4^+^ microglia and a subpopulation of 30% of 5xFAD microglia^51^ (Supplementary Fig. 10c). We confirmed *Hif1a* regulon activity in 54.3% of the cluster (from study^51^) transcriptionally most similar to DAM microglia^13^, suggesting that XO4^+^ cells may be a subset of DAM microglia, defined not only by their transcriptomic signature but also by active plaque phagocytosis associated with a transient or reversible signature. To investigate the role of the *Hif1a* regulon in driving XO4^+^ cells in vitro, cells of the BV2 mouse microglial line expressing mCherry-tagged *Hif1a* shRNA or mCherry were treated with AlexaFluor488 (AF488)-labelled fAβ for 24 h. Transcriptionally, a number of XO4^+^ genes predicted to be part of the *Hif1a* regulon showed *Hif1a*-dependent induction in response to fAβ, namely *Spp1* and *Igf1* (Fig. 4f, *p* = 0.0025, Fisher’s combined probability test, and Supplementary Fig. 10d). To investigate the role of fAβ and *Hif1a* in functional synaptosome phagocytosis in vitro, cells were treated with fluorescent-blue-labelled synaptosomes for the last 1.5 h of the AF488-fAβ incubation (Fig. 4g). fAβ phagocytosis induced a synaptosome-phagocytic phenotype (Fig. 4g and Supplementary Fig. 10e, *p* = 0.0026, two-way ANOVA, Tukey’s multiple comparison test) akin to XO4^+^ cells, which was modulated via *Hif1a* (Fig. 4g, *p* = 6.0 × 10^−6^, 2-way ANOVA, Tukey’s multiple comparison test). fAβ internalization was associated with increased HIF1A protein expression in vitro (Fig. 4h). Moreover, *Hif1a* overexpression synergized with fAβ phagocytosis to enhance synaptosome phagocytosis (Fig. 4i, *p* = 0.0253, one-way ANOVA with Holm-Sidak’s multiple comparison test), and slightly but significantly increased fAβ phagocytosis (Supplementary Fig. 10f, 10% increase, *p* = 0.0382, paired *t*-test). Together these data support a role of HIF1A and fAβ phagocytosis in synaptosome phagocytosis in microglia in vitro.

### Microglia isolated from the brains of AD patients display similarities to the gene expression signature associated with XO4^+^ microglia

**Fig. 5:**
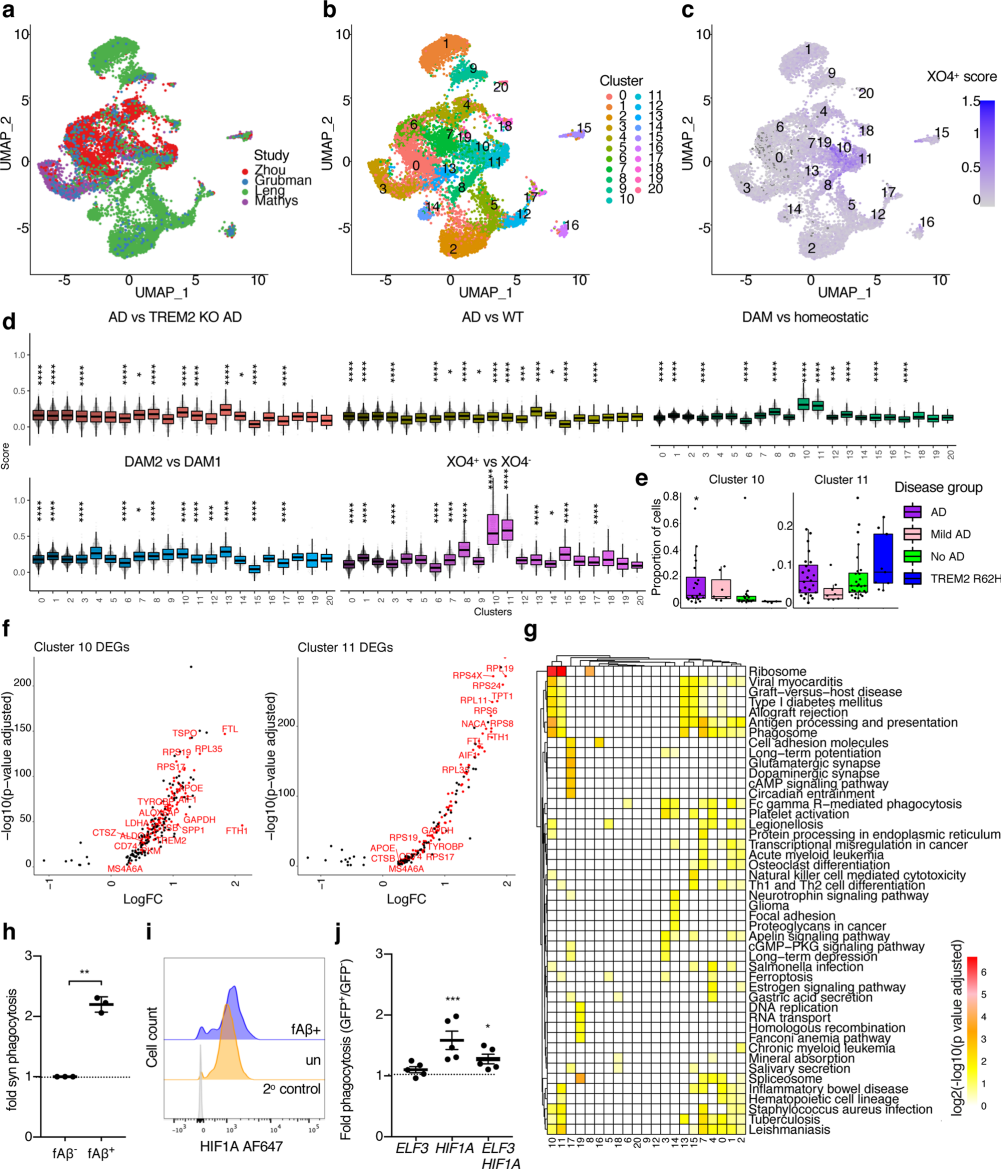
The gene expression signature associated with XO4^+^ microglia is molecularly and functionally replicated in microglia isolated from the brains of AD patients and non-AD patients. **a**–**c** UMAP projection of single microglia nuclei from control and AD patient entorhinal and frontal cortex samples, combined by integrating data from^51–54^, comprising 102 patients; AD (*n* = 5891 microglia nuclei), mild AD (*n* = 1591 microglia nuclei), controls (*n* = 2988 microglia nuclei), Other Dementia (*n* = 3 microglia nuclei) and TREM2 R62H variant (*n* = 1458 microglia nuclei). Clustering and analysis of signature scores is performed using Seurat v3. UMAP projection is coloured by (**a**) study of origin, (**b**) Seurat cluster and (**c**) XO4^+^ score. **d** Box plots for gene signature scores in each human microglial cluster for the AD vs Trem2KO AD signature, AD vs WT signature^51^, DAM vs homeostatic, and DAM2 vs DAM1 signatures^13^. The lower, middle and upper hinges represent the lower quartile, median and upper quartile, respectively, while the upper and lower whiskers extend ±1.5 times of the interquartile range from the hinges. For each signature score category, pairwise Wilcoxon test between each cluster and base mean was computed. Multiple testing was corrected for using Bonferroni correction. *p < 0.05, **p < 0.01; ***p < 0.001, ****p < 0.0001, exact *p* values are provided in the Source data. **e** The proportion of cells in Clusters 10 and 11 in patients with any cells in Cluster 10 or Cluster 11, respectively (please see Supplementary Fig. 11 for sample size details), grouped according to disease status and/or TREM2 genotype (^*^p = 0.047, Wilcoxon Test with No AD as reference). The lower, middle, and upper hinges represent the lower quartile, median and upper quartile, respectively, while the upper and lower whiskers extend ±1.5 times of the interquartile range from the hinges. **f** Cluster 10 and Cluster 11 DEGs relative to all other human microglia clusters (adjusted *p*-value < 0.05). Genes of interest associated with XO4^+^ microglia are highlighted in red. **g** Heatmap of enriched KEGG pathways in the human microglial Seurat clusters, coloured by log_2_(-log_10_(adjusted *p*-value)). **h** Fluorescently labelled synaptosome internalization by human primary microglia treated with AF647-labelled fAβ. The data are mean ± SEM of 3 independent biological replicates and are expressed as fold change in synaptosome internalization relative to non-treated microglia. Differences are reported between AF488-fAβ^+^ and AF488-fAβ^−^ cells tested from within the same well. **i** Histograms showing fluorescence intensity of HIF1A intracellular staining in AF488-fAβ^+^ and AF488-fAβ^−^ human primary microglia assayed from within the same well. Secondary antibody control cells are stained with AF647 secondary antibodies alone. **j** Fluorescently labelled synaptosome internalization by primary microglia transfected with GFP-tagged inducible *HIF1A* and/or *ELF3* overexpression constructs. The data are the mean ± SEM of 5 independent biological replicates and are expressed as fold change in synaptosome internalization between GFP^+^ and GFP^−^ (non-transfected) cells tested from within the same well. *p = 0.0188, ***p = 0.0002 by two-way ANOVA and Sidak’s multiple comparison test on the raw synaptosome internalization percentages.

Studies have shown that plaque-adjacent microglia in human post mortem AD brains upregulate LPL^13^ and downregulate P2RY12^14^, consistent with the gene expression signature associated with XO4^+^ microglia surrounding plaques. To assess whether the gene expression signature associated with XO4^+^ microglia is present in microglia isolated from human brains, we performed a comprehensive integration and analysis of microglia from the publicly available single-nucleus datasets from the brains of AD patients and non-AD individuals (Fig. 5a), comprising single cells from 102 human entorhinal and prefrontal cortex samples from 4 independent datasets^51–54^ (total 11,931 microglial nuclei, 2988 controls, 1591 mild AD, 5891 AD, 1458 with *TREM2* R62H variant, and 3 nuclei of Other Dementia). Microglia from human brains formed 21 clusters (Fig. 5b), some specific to individual datasets (e.g., Clusters 1, 2, 3, 5, 6, 12; with >90% cells originating from one dataset; Supplementary Fig. 11a, b), whereas others contained microglia from all studies (e.g., Clusters 0, 7, 9, 10 and 11). To examine the conservation of the gene expression signature associated with XO4^+^ microglia, we calculated the signature scores of the phagocytosis DEG set (XO4^+^ vs XO4^−^) in microglia isolated from human brains (Fig. 5c). Importantly, the score for the gene expression signature associated with XO4^+^ microglia was significantly enriched in Clusters 10 and 11 (Fig. 5c, *p* = 6.8 × 10^−58^, 2.1 × 10^−24^, respectively, Wilcoxon test), containing cells from all 4 studies (Supplementary Fig. 11a). We observed that, compared to additional gene signatures reported for mouse microglia, the phagocytic signature associated with XO4^+^ microglia identified in this study shows greatest variation across clusters, and can be recapitulated in microglial nuclei from human brains to a greater extent than the gene expression signatures associated with DAM or Trem2KO microglia (Fig. 5d and Supplementary Fig. 11c). The gene expression signature associated with XO4^+^ microglia was not enriched in microglia isolated from AD patients (Supplementary Fig. 11b and Supplementary Data 8), however, for patients containing any cells with a high XO4^+^ score (i.e., in Cluster 10 or Cluster 11), the proportion of total microglia in Cluster 10, but not in Cluster 11, was significantly higher in AD patients (Fig. 5e and Supplementary Fig. 11d, e; *p* = 0.047, Wilcoxon test). In addition, XO4^+^ scores were significantly lower in *TREM2* R62H cells (Supplementary Fig. 11e; *p* < 2.22 × 10^−16^, Wilcoxon test). This analysis suggests that, as for DAM microglia, aspects of the gene expression signature associated with XO4^+^ microglia described here are likely to be TREM2 dependent. The DEGs for Clusters 10 and 11 significantly overlapped with the gene expression signature associated with XO4^+^ microglia, particularly ribosomal subunit genes (Fig. 5f and Supplementary Fig. 11f). Cluster 10 DEGs overlap with 20% of the genes associated with XO4^+^ microglia compared to 10% of the DAM signature genes, *p* = 3.50 × 10^−7^, one-sided two-proportions *z*-test). As observed in the mouse gene expression signature associated with XO4^+^ microglia, we found transcriptional changes to AD GWAS risk genes and their interacting partners: *TREM2, APOE, TYROBP* and a number of *Hif1a* regulon target genes including *ALDOA, LDHA* and *PKM* encoding for the enzymes: aldolase, lactate dehydrogenase, pyruvate kinase (Supplementary Data 8). Similarly to mice, human microglia clusters with the XO4^+^-associated gene expression signature (Clusters 10 and 11) were enriched for functional processes relating to ribosome, phagosome and antigen presentation (Fig. 5g).

We next semi-quantified the amount of post-synaptic material in plaque-adjacent and distal microglia in human frontal cortex sections (Supplementary Fig. 12a–c). Analogous to 5xFAD mice, we observed that plaque-associated microglia in human AD patients contained modestly, but significantly less PSD95 staining than in non-plaque-associated microglia from the same brain region (*p* = 0.02, 1-tailed one-sample *t*-test; Supplementary Fig. 12c). As in mouse BV2 cells, primary human microglia in vitro that had internalized AF488-labelled fAβ had an increased capacity for phagocytosis of fluorescent synaptosomes (Fig. 5h; *p* < 0.0001, 2-tailed *t*-test), and increased HIF1A expression (Fig. 5i). We transfected primary human microglia in vitro with dox-inducible GFP-tagged constructs to genetically turn on *HIF1A* or *ELF3*. We found that *HIF1A*, but not *ELF3*, overexpression increased synaptosome phagocytosis, consistent with a role for *HIF1A* in regulating XO4^+^ functions in vitro (Fig. 5j and Supplementary Fig. 12d, e; *p* = 0.0002, two-way ANOVA and Sidak’s multiple comparison test). Together, our data show that the gene expression signature associated with XO4^+^ microglia is recapitulated in a subset of microglia isolated from human brains and can be functionally modulated via *HIF1A* or fAβ to increase synaptosome phagocytosis in vitro.

### In vitro modulation of the gene expression signature associated with XO4^+^ microglia through small molecules upstream of *HIF1A*

**Fig. 6:**
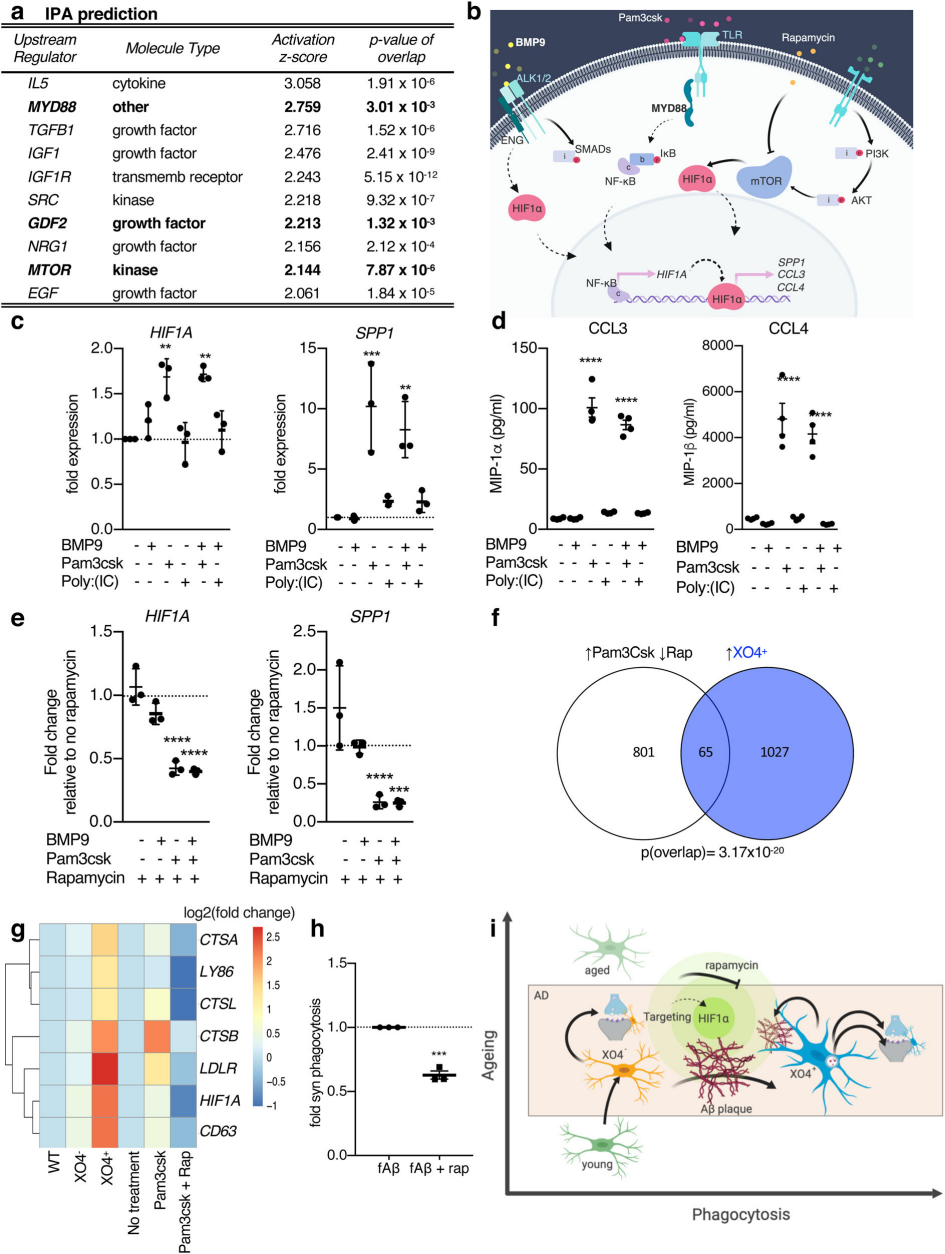
The gene expression signature associated with XO4^+^ microglia can be manipulated through the *Hif1a* regulon. **a** Top ten activators of the *Hif1a* regulon predicted by IPA. The activation *z*-score is a statistical measure of the match between the expected relationship direction of regulation and the observed gene expression; positive *z*-scores are indicative of predicted activation. *p*-Value of overlap refers to the significance of the overlap between the *Hif1a* regulon gene set and the regulated target genes predicted by IPA. The 3 predicted regulators tested in this figure are in bold. **b** Cartoon diagram of hypothesis generated by IPA. **c** Stimulation of iMGLs with MyD88-dependent TLR-agonist Pam3csk (alone or with BMP9) induces genes associated with XO4^+^ microglia within the *Hif1a* regulon as identified by qPCR (*HIF1A* ^**^p = 0.0014 and *p* = 0.0012, respectively, *SPP1* ^***^p = 0.00024 and ^**^p = 0.0015 by one-way ANOVA and Holm-Sidak post-test), *n* = 3 independent experiments. **d** Cytometric bead array (^****^p < 0.0001 by one-way ANOVA and Holm-Sidak post-test, CCL3: F(5,18)=137.1; CCL4: F(5,18)=42.04), *n* = 4 independent experiments. MyD88-independent TLR stimulation (Poly:IC) does not shift iMGLs towards a gene expression signature associated with XO4^+^ microglia^.^ Data are fold changes normalized to non-treated cells. **e** MyD88-dependent expression of genes associated with XO4^+^ microglia is modulated by rapamycin. Data are fold changes induced by rapamycin normalized to each respective treatment in the absence of rapamycin. *HIF1A* ^****^p = 3.5 × 10^−7^ and 1.7 × 10^−7^ and *SPP1* ^****^p = 6.5 × 10^−5^ and ^***^p = 0.0006, respectively, by two-way ANOVA compared to non-rapamycin-treated cells and Holm-Sidak post-test. *n* = 3 independent experiments. **f** Venn diagram showing the overlap between a XO4^+^-like state induced in iMGLs using Pam3csk and reversed by rapamycin (RNA-seq, *n* = 4 independent experiments) as predicted by ingenuity pathway analysis (IPA) with the mouse gene expression signature associated with XO4^+^ microglia^,^ as measured by RNA-seq (*p* = 3.17 × 10^−20^, hypergeometric test). **g** Representative gene expression heatmap of selected genes that are part of the overlap, showing expression levels in mice (WT, 5xFAD XO4^−^, 5xFAD XO4^+^) and human iMGLs (non-treated, Pam3csk and Pam3csk+rapamycin). **h** Fluorescently labelled synaptosome internalization by iMGLs treated with amyloid fibrils, alone, or in combination with rapamycin for 48 h, as measured by FACS. The data are presented as mean ± SEM, and *p* = 0.0002 by unpaired *t*-test, *n* = 3 independent experiments. **i** Proposed model of generation and regulation of microglia diversity in AD, created with BioRender.com.

To infer upstream small molecules to target the *Hif1a* regulon and in turn the XO4^+^ network, we employed Ingenuity Pathway Analysis (IPA)^55^. Among others (Fig. 6a and Supplementary Data 9), IPA identified BMP9 (or GDF2), a ligand of the TGF-β superfamily, the Toll-like receptor (TLR) adaptor MyD88, and mTOR, which were previously involved in restricting amyloidosis^56^, microglial response to pathogens^57^, and pro-inflammatory microglia^58^ (Fig. 6b). We validated these predictions using human ESC-derived microglia-like cells (iMGLs)^59,60^ in vitro. Stimulation of iMGLs with the MyD88-dependent TLR1/2 agonist, Pam3csk, alone or with BMP9, resulted in upregulation of *HIF1A* mRNA (Fig. 6c; *p* = 0.0014 and *p* = 0.0012, respectively, by one-way ANOVA and Tukey’s multiple comparison test), downstream targets *SPP1* and *GAPDH*, and secretion of chemokines MIP1α and MIPβ, encoded by the genes associated with XO4^+^ microglia, *CCL3* and *CCL4*, respectively (Fig. 6c, d and Supplementary Fig. 13a). As predicted by IPA, treatment of cells in vitro with the mTOR inhibitor, rapamycin, blocked MyD88/BMP9-dependent induction of microglial genes within the *Hif1a* regulon associated with the XO4^+^ signature (Fig. 6e). Moreover, rapamycin treatment reduced TREM2 expression at both the RNA and protein levels and induced the homeostatic marker CX3CR1 (Supplementary Fig. 13a, b). The network of genes induced by Pam3csk and repressed by rapamycin in vitro significantly overlapped with the upregulated genes associated with XO4^+^ microglia (65 genes overlap, *p* = 3.17 × 10^−20^, hypergeometric test; Supplementary Fig. 13c) that included *CTSB, LDLR, HIF1A, PKM, LDHA*, and was enriched for GOs including lysosome (Fig. 6f, g and Supplementary Data 10). Notwithstanding the widespread effects of Pam3csk on inflammatory processes and rapamycin on cell growth, proliferation and survival, we were able to modulate the function of iMGLs in vitro, as rapamycin was able to reduce synaptosome phagocytosis in fAβ-treated iMGLs (Fig. 6h; *p* = 0.0002, 2-tailed Student’s *t*-test). Together these data show not only the predicted regulatory role of the *Hif1a* regulon on the gene expression signature associated with XO4^+^ microglia (Fig. 4e), but also that components of this regulon can be modulated by upstream small molecules in vitro to control microglia cell fate along the homeostatic-to-XO4^+^ axis.

## Discussion

Recent studies^13,14^ identified a novel microglial phenotype near Aβ plaques that was dependent on *Trem2* and *Apoe* in two mouse models of AD. Here, we expanded on previous work by firstly defining the specific gene expression signature of AD microglia that have actively phagocytosed plaques in vivo, and then proposed the mechanisms responsible for induction and maintenance of this phenotype. Furthermore, we uncovered a similar gene expression signature in a subset of microglia isolated from the brains of AD patients and started to untangle the controversy regarding the beneficial^61^ or detrimental^14^ functional role of these cells. A key challenge is the direct isolation of these phenotypically different cells. Keren-Shaul et al.^13^ approached this problem through single-cell transcriptomics, which did not permit additional functional characterizations due to the destructive nature of the technique. Krasemann et al.^14^ chose instead to purify these cells through either sorting for CLEC7A, also present on a subset of WT microglia, or purifying microglia that phagocytosed apoptotic neurons injected stereotaxically into mice. Here, we used a direct approach with methoxy-XO4 labelling^25^ to transcriptionally profile a functionally defined microglial subset actively phagocytosing the plaques at the time of experiment. We note that our data do not rule out a possibility that the XO4^−^ population may include a mix of microglia that never contained internalized fibrillar amyloid as well as microglia that were XO4^+^ and have subsequently degraded amyloid, however, these cells are both functionally and transcriptionally indistinguishable by our analyses.

We show that two distinct but interrelated processes are associated with microglial changes in AD: (i) accelerated aging and (ii) direct response to plaque phagocytosis (resulting in XO4^+^ cells). Keren-Shaul et al.^13^ reported that 3% of aged WT microglia exhibited a DAM signature that was undetected in younger animals. Interestingly, in the present study, no 24 m WT microglia clustered together with the XO4^+^ microglia (Fig. 2b), highlighting the specificity of the gene expression signature associated with XO4^+^ microglia to phagocytosis. As the DAM population^13^ is not defined functionally by plaque uptake and hence may collectively be comprised of both XO4^+^ and XO4^−^ microglia, it was not possible to assess an independent effect of ageing and amyloid phagocytosis on DAM. Our data show that the ageing-associated signature acquired by XO4^−^ microglia is independent of uptake of amyloid plaque, thus allowing us to disentangle the ageing from amyloid phagocytic processes. The transcriptional signature of aged human microglia has been previously described^39,62^. Post mortem microglia from cognitively normal subjects displayed similar gene expression profiles to mouse microglia, but human and mouse signatures diverged significantly with ageing^62^. Furthermore, The HuMi_Aged dataset is enriched for susceptibility genes for LOAD^39^, which is consistent with our mouse data. Several recent studies suggested AD-specific gene upregulation of *TREM2, TYROBP, CLEC7A, CD68, CD34*, *SPP1* and various MHC Class II genes^10,63^ and positive LPL staining in ThioS^+^ (plaque-associated) microglia in 4 out of 5 human AD patients tested^13^. Moreover, it was recently reported that a subset of microglia in the brains of AD patients display a *SPP1*^*+*^*CTSD*^*+*^ profile consistent with the gene expression signature associated with XO4^+^ microglia^52^. It is worthwhile noting that the XO4^+^-associated gene expression signature in 5xFAD mice exists under conditions of plaque deposition with little tau pathology^64^, whereas human AD pathology invariably includes tau, which likely produces altered signatures and responses in microglia.

Our results indicate that XO4^−^ microglia cells are functionally deregulated, set on a trajectory of accelerated ageing and not a transcriptional intermediate en route to become XO4^+^. XO4^−^ microglia are different from previously reported Stage I and II DAM^13^. XO4^−^ microglia contain more steady-state internal synaptic material than XO4^+^ microglia (Fig. 4a, b), despite the reduced capacity of XO4^−^ microglia for active phagocytosis of synaptosomes and fAβ ex vivo (Fig. 4c, d). Also, XO4^−^ microglia do not upregulate TREM2 (Supplementary Fig. 8e) and do not migrate towards plaques despite some capacity to internalize amyloid (Fig. 1j). On the other hand, plaque phagocytosis resulting in a gene expression signature associated with XO4^+^ microglia primes microglia for enhanced phagocytosis of synaptosomes, although it is important to note that the efficiency of individual phagocytic processes can be dependent on experimental conditions^65–67^. Rapid pruning of damaged synapses near dystrophic neurites localized around plaques appears to be, at least initially, protective and may go awry later in disease progression as described before^68^. Our results support previous studies showing improved behaviour in AD mouse models associated with enhanced microglial amyloid plaque phagocytosis in response to scanning ultrasound^69^, and treatment with IL-33 that signals exclusively via MyD88^70^. Similarly, genetic human data suggest that both DAM and XO4^+^ microglia are beneficial in AD and may be a protective phagocytic phenotype that may enhance plaque clearance^61^. However, Gal3, a TREM2 ligand encoded by the *Lgals3* gene specific to the XO4^+^-associated gene expression signature, is toxic in 5xFAD mice^71^. Gal3 is released in response to fAβ treatment in vitro, found in close association with plaques in human AD microglia, and Gal3 knockout in 5xFAD mice reduced plaques and improved memory function^71^. Similarly, targeting MyD88 has also yielded contradictory effects in AD models^72,73^ possibly due to differential effects on XO4^+^ and XO4^−^ populations. In the present study, targeting Myd88 induce lysosomal genes and LDLR, a lipoprotein receptor shown to bind APOE and Aβ, and regulate Aβ phagocytosis in astrocytes^74^.

We showed that microglia possess an innate capacity to activate the gene expression signature associated with XO4^+^ microglia, and phagocytosis of amyloid plaques per se is sufficient for the generation of this AD-associated transcriptomic signature in vitro. We identified the *Hif1a* regulon as one of the players underlying the molecular mechanism associated with the transition to the XO4^+^ phenotype and synaptosome phagocytosis. Our demonstration that this pathway can be modulated in ESC-derived iMGLs in vitro opens new avenues to examine how and to what extent the patients’ genetic background and/or additional neurodegeneration-associated molecules may influence the manipulation of the XO4^+/−^ axis. A recent study showed that α-synuclein application in vitro induced HIF1A accumulation through TLR7/8 in microglia, stimulating their migration^75^. *Hif1a* activation and increased protein production have also been recently described for mucolipidosis Type IV microglia^76^, yet the signature was distinct from other neurodegeneration-associated microglia signatures. From our examination of the literature, there is no published evidence of HIF1A involvement in microglial responses to tau or huntingtin. There is evidence of a microglial signature in ALS *SOD1G93A* mice that included upregulation of some *Hif1a* signature genes including *Tyrobp* and *Igf1*^77^, although the origin of this signature is not clear. It is interesting to note that *Hif1a* is not a signature gene described by Krasemann et al.^14^, who examined a common disease-associated signature in APP/PS1 mice, EAE model mice and SOD1^G93A^ mice. A *Hif1a* epigenetic and transcriptomic signature was recently identified in microglia following immune training by peripheral LPS administration in APP/PS1 mice^65^. We found little overlap between the *Hif1a* module reported in^65^ and the *Hif1a* regulon identified here (Supplementary Fig. 13d), reinforcing the importance of microglial fine tuning of context-dependent responses to specific stimuli. These data suggest that while certain aspects of the microglial signature overlap in neurodegeneration (and aging), such as *Apoe, Trem2, Cd11c*, etc., the *Hif1a* signature we describe appears to be specific to the XO4^+^-labelled subset of microglia in 5xFAD mice that could represent a subset of previously identified DAM microglia. Recent analyses showed a reproducible protein signature in 3 independent patient cohorts (total 197 patients) specifically in AD CSF relative to depression and mild cognitive impairment (MCI)^78^. Importantly, 20% of the upregulated proteins were part of the *Hif1a* downstream network predicted here and included the HIF1A target proteins ALDOA, SPP1, PLD3, PGK1, FABP3, LDHA and STMN1, and the XO4^+^-specific HIF1A target protein PKM was recently identified as a novel AD CSF biomarker^79^.

In summary, we hypothesize a model whereby as microglia age, they are set on a transcriptional trajectory which is accelerated in an AD microenvironment. However, upon plaque phagocytosis, microglia re-route on a different trajectory which is associated with the *Hif1a* regulon, resulting in enhanced phagocytosis of synaptic components around plaques, and a feed-forward loop that enhances Aβ phagocytosis (Fig. 6i). We show how the microglial gene signatures we uncovered can be harnessed by computational prediction of microglial subset-targeting drugs, network pharmacology and repositioning approaches. As further functional analyses shed light on beneficial or detrimental roles of phagocytic XO4^+^ microglia in AD, potential therapeutic strategies could involve targeted conversion between XO4^−^ and XO4^+^ microglia by using small molecules to tune the key transcriptional networks.

## Methods

### Animals

Heterozygous 5xFAD transgenic mice (B6SJL hybrid background) over-expressing FAD mutant forms of human APP (Swedish mutation K670/ 671NL, London mutation V717I, and Florida mutation I716V) and PSEN1 (M146L and L286V), regulated by the neuron-specific mouse *Thy1* promoter^64^ and CX3CR1^GFP^ were housed at Monash Animal Research Platform (MARP) under specific pathogen-free conditions in a day–night controlled light cycle, provided with food and water ad libitum. Animals were used for experiments at different ages through adulthood, as indicated, without undergoing any procedures prior to their stated use. All use and handling of animals for experimentation was approved by Monash Animal Ethics Committee (MARP/2016/112) and conformed to national and institutional guidelines.

### Human patient demographics

Paraffin-embedded human frontal cortex sections of post mortem Alzheimer’s disease and non-disease age-matched individuals (10 μm) were obtained from the Victorian Brain Bank (Ethics Approval: for patient tissue banking and consent: University of Melbourne HREC Approval No.: 1545740; patient demographics below). The study participants were allocated into disease or control groups based on the overall amyloid and tau pathology (Table 1).

**Table 1:** The age, gender, post mortem interval (PMI) and diagnosis for the controls and cases used for immunofluorescence analyses.

Case no.	Age (years)	Gender	PMI (h)	Diagnosis
Ct7	59	Female	30	Control
Ct8	63.4	Female	30.5	Control
Ct9	65.8	Female	43	Control
Ct1	67.3	Female	24	Control
Ct10	68.3	Female	71.5	Control
Ct11	78.8	Female	19	Control
Ct12	79	Female	32	Control
Ct13	80.7	Female	59	Control
Ct14	81.2	Female	25	Control
Ct2	82.7	Female	28.5	Control
AD7	62.5	Female	5.5	AD
AD8	64.7	Female	21	AD
AD9	65.9	Female	40	AD
AD3	67.8	Female	21	AD
AD10	68.4	Female	31	AD
AD11	78.9	Female	19.5	AD
AD12	79.9	Female	35	AD
AD13	80.3	Female	62	AD
AD14	82.5	Female	15.5	AD
AD15	82.7	Female	8	AD

### Acute isolation of microglia and fluorescence-activated cell sorting

At 2 h prior to killing, mice were injected intraperitoneally with methoxy-XO4 (2 mg/ml in 1:1 ratio of DMSO to 0.9% (w/v) NaCl, pH 12) at 5 mg/kg. Mice were euthanized by CO_2_ and transcardially perfused with ice-cold PBS prior to brain extraction. Whole brains, excluding brain stem and olfactory bulbs, were dissected into cerebellum and non-cerebellum regions for microglia isolation. Single-cell suspensions were prepared from brain tissues by mechanical dissociation using mesh of decreasing sizes from 250 to 70 μm and enriched for microglia by density gradient separation^80^. Briefly, the cell pellet was resuspended in 70% (v/v) isotonic Percoll (1x PBS + 90% (v/v) Percoll), overlaid with 37% (v/v) isotonic Percoll and centrifuged with slow acceleration and no brake at 2000*g* for 20 min at 4 °C. The microglia-enriched cell population isolated from the 37–70% interphase was diluted 1:5 in ice-cold PBS and recovered by cold centrifugation at maximum speed for 1 min in microcentrifuge tubes. The cell pellet was then stained with antibodies to microglial cell surface markers (CD11b-BV650, 1:200 Biolegend, #141723; CD45-BV786, 1:200, BD Biosciences #564225; CX3CR1-FITC, 1:100, Biolegend, #149019; CD11a, 1:20, BD Biosciences, #558191, TREM2-APC, 1:10, R&D Systems, #FAB17291N; CD33-PE, 1:20, eBioscience, #12-0331-82; CD115-BV711, 1:40, Biolegend, #135515) for isolation using the FACSAria™ III cell sorter. Microglia were defined as live/propidium iodide (PI)^−^ (Sigma-Aldrich, St. Louis, MO, #P4864), CD11b^+^, CD45^lo^, CX3CR1^+^ single cells and were negative for CD11a (Gating strategy in Supplementary Fig. 1a). The XO4^+^ population gate was set using methoxy-XO4-injected WT animals. XO4^+^ and XO4^−^ microglial populations were sorted separately for further analysis by bulk RNA-seq, nano LC-SWATH-MS (20,000 cells per sample) and scRNA-seq.

### viSNE analysis

The Cytobank platform (Fluidigm, South San Francisco, California) was utilized to generate viSNE plots^81^ from Flow Cytometry Standard files. Analyses were performed on live/propidium iodide (PI)^−^ single-cell population. A total of 40,000 events were sampled to generate viSNE maps. Seven fluorescent channels (CD11b, CX3CR1, CD45, CD115, CD33, TREM2 and methoxy-XO4) were engaged for dimensionality reduction. The run was performed 5 times to ensure the stability of the presented outcome.

### RNA-seq library construction and sequencing

RNA extraction from 1 to 10 × 10^4^ FACS-sorted microglia or iPS-derived iMGLs was performed on the QIAcube (Qiagen) using the RNeasy Micro Kit (Qiagen, #74004) and RNA quality was assessed using the Bioanalyser (Agilent RNA 6000 Pico kit; #5067-1513). The libraries were prepared using 0.5–2 ng of non-cerebellum microglia RNA samples with RIN value ≥ 7 and cerebellar microglia with RIN value ≥ 6. An 8 bp sample index (Supplementary Data 11) and a 10 bp unique molecular identifier (UMI) were added during initial poly(A) priming and pooled samples were amplified using a template switching oligonucleotide. The Illumina P5 (5ʹ AAT GAT ACG GCG ACC ACC GA 3ʹ) and P7 (5ʹ CAA GCA GAA GAC GGC ATA CGA GAT 3ʹ) sequences were added by PCR and Nextera transposase, respectively. The library was designed so that the forward read (R1) utilizes a custom primer (5ʹ GCC TGT CCG CGG AAG CAG TGG TAT CAA CGC AGA GTA C 3ʹ) to sequence directly into the index and then the 10 bp UMI. The reverse read (R2) uses the standard R2 primer to sequence the cDNA in the sense direction for transcript identification. Sequencing was performed on the NextSeq550 (Illumina), using the V2 High output kit (Illumina, #TG-160-2005) in accordance with the Illumina Protocol 15046563 v02, generating 2 reads per cluster composed of a 19 bp R1 and a 72 bp R2.

### Demultiplexing and mapping

Sequencing reads for the murine microglia dataset were sample demultiplexed with Je demultiplex from the JE suite^82^ using sequence barcodes in Supplementary Data 11. Short-sequence UMIs from read pair 1 of the demultiplexed sample sequencing reads were discarded from both sequencing read pairs with Prinseq (minimum length 9)^83^. Remaining UMIs were clipped with Je clip and added to the sequencing read header to allow UMI deduplication post read mapping. Demultiplexed UMI-tagged sequencing reads were filter-trimmed with Trimmomatic^84^ and aligned to the mouse genome (GENCODE’s GRCm38 primary assembly annotation version vM15) using STAR^85^ (only sequencing reads from pair 2 were used for transcript quantification). Read deduplication based on UMIs was performed with Je MarkDupes and transcript read counts calculated with featureCounts^86^. For the in vitro bulk RNA sequencing dataset, demultiplexing was performed as we recently described^60^. In short, we used in-house pipelines including a fork of sabre tools (https://github.com/serine/sabre), and demultiplexed UMI-tagged sequencing reads were aligned to the human genome (Ensembl GRCh38 primary assembly) using RNAsik^87^.

### Analysis of microglia bulk RNA-seq

The log_2_-transformed normalized gene expression from bulk RNA-seq was obtained using the Variance Stabilizing Transformation (VST) from the DESeq2 package (version 1.18) in R^88^. PCA (Fig. 1e) and hierarchical clustering (Supplementary Fig. 1d) were then performed on the VST counts. To investigate if the sequencing batch had an effect on the gene expression, we performed a covariate analysis. For each covariate of interest (XO4, batch, region, age, genotype and gender), a likelihood ratio test (LRT) was performed using the DESeq2 package, comparing the full model comprising all covariates and the reduced model which omits the covariate of interest. Thus, genes that are statistically significant under the LRT are genes whose variation in expression levels could be explained by the covariate of interest. The covariate analysis (Supplementary Fig. 2a) revealed that only 8 genes contribute to gender-related variation (FDR < 0.01). Thus, all subsequent analyses were performed excluding the gender covariate and both male and female microglial transcriptomes were analysed together. The covariate analysis was then performed again without the gender covariate to identify genes that are specific to the XO4 covariate (Fig. 1h_i_). GO and KEGG terms overrepresentation analyses were performed using the gProfileR package in R^89^. This covariate analysis also revealed a large number of genes associated with batch (997 genes, FDR < 0.01) and these genes significantly overlap with the region-related (*p* = 1.5 × 10^−53^ by hypergeometric test), age-related (*p* = 2.2 × 10^−28^ by hypergeometric test) and XO4-related genes (*p* = 1.5 × 10^−201^ by hypergeometric test; Supplementary Fig. 14a–c). Thus, the batch covariate was included in all subsequent analyses to account for batch effects. To generate the gene cytometry plots (Fig. 1f, g), a generalized linear model was constructed with the covariates XO4, batch, region, age and genotype. Separate pairwise differential expression analyses were then performed between XO4^+^ vs XO4^−^, 4 m vs 1 m, and 6 m vs 1 m microglia samples, respectively. For each differential expression analysis and each gene, a gene score was then calculated as the product of the absolute value of the log_2_ fold change and negative of the log-transformed FDR, abs(LFC)*-log_10_(FDR), combining the effect size and statistical significance of the differential expression^90^. The gene scores for XO4 and age differential expression were then plotted to give the gene cytometry plots.

For comparison with Kang et al.^31^, raw RNA-sequencing data were downloaded from Gene Expression Omnibus (GSE117646). Filtered gene list was taken from https://www.fryerlab.com/ribotag. limma (3.34.9)^91^ was used to find DEGs between the following groups: (i) APP and control, (ii) tau and control, (iii) old (24 m) male mice and young (3 m) male mice. Briefly, lmfit() was used and eBayes() was set to trend = TRUE and robust = TRUE. This yielded 359 amyloid, 282 tau and 2762 ageing DEGs which were defined with adjusted *p*-value < 0.05. VennDiagram^92^ (1.6.20) was used to plot all Venn diagrams.

For comparison with Haimon et al.^38^, we obtained gene signature of Cluster 2a consisting of 190 transcripts. These genes are enriched in both the transcriptomes and translatomes of sorted cells, thereby potentially representing artifactual perturbations caused by the cell-sorting procedure.

For comparison with Krasemann et al.^14^ and Friedman et al.^33^, we obtained the MGnD (*n* = 96 genes) and Neurodegenerative (*n* = 134 genes) signatures, respectively. For comparison with Keren-Shaul et al.^13^, DAM signature was defined by obtaining DEGs between DAM and homeostatic microglia with FDR < 0.05 resulting in 1176 genes. For GO enrichment analysis, we used clusterProfiler^93^ while bitr() was employed to map gene symbols to entrezIDs using org.Mm.eg.db (3.10.0)^94^ as reference database. simplify() was used to remove redundant GO terms using the following parameters: cut-off = 0.3, by = p.adjust, select_fun = min. Note that for all comparisons, our single-cell XO4^+^ and bulk RNA-seq XO4^+^ contain 536 genes (FDR < 0.05) and 2810 genes (FDR < 0.01), respectively.

### Single-cell sequencing

In all, 5000 microglia from each population (including XO4^+^ and XO4^−^ microglia, and 6, 9, 12 and 24 m CX3CR1^GFP^ microglia) were sorted into DMEM/F12 media (supplemented with 5% (v/v) FBS, 50 U/ml Penicillin and 50 µg/ml Streptomycin), centrifuged at 12,000*g* for 2 min at 4 °C and resuspended in 35 μl of PBS containing 0.04% (w/v) BSA (0.22 µm filtered). The samples were then diluted with nuclease-free water in accordance to 10X  Genomics single-cell protocol guidelines to achieve a target cell recovery of approximately 800 cells/sample (for 6 m WT, 24 m WT, 6 m XO4^−^ and 6 m XO4^+^ dataset), and target recovery of 10,000 cells per sample (age dataset 6, 9, 12 m CX3CR1^GFP^ microglia). Single-cell capture, RNA-seq library construction and sequencing were carried out at Micromon, Monash University using the 10X Genomics Chromium system (10X Genomics, Chromium Single Cell V(D)J Reagent Kits with Feature Barcoding technology for Cell Surface Protein, Document Number CG000186 Rev A, 10X Genomics, 2019, July 25). Library construction was performed by poly-A selection from total RNA using 10X Chromium controller with Chromium Single Cell 3ʹ Reagent Kit V2 (10X Genomics, #PN-120237). Sequencing was performed on one high-output lane of an Illumina NextSeq 550 (Illumina, California, USA) in paired-read 150 bp format. Chromium barcodes were used for demultiplexing and FASTQ files were generated using the Cellranger^95^ mkfastq pipeline. Alignment, filtering and UMI counting were performed using Cellranger count. To improve detection of microglia, due to their low RNA content, Cellranger reanalyse was used with the –force-cells option set at the inflection point when number of barcodes is plotted against the number of UMIs. Cells were manually filtered such that barcodes containing at least 10 counts corresponding to *Cx3cr1, P2ry12* or *Fcrls* genes and less than 6 counts corresponding to *Epcam* or *Ephb2* were classified as microglia, resulting in a total of 991 cells from the 4 FACS-sorted microglial populations.

### Single-cell analysis in the mouse AD model

The original mapped matrix dimensions were 10,484 genes by 991 cells. For quality control, various filtering steps were implemented. Genes without any counts in any of the cells were discarded. Cells were filtered by total counts and total features (genes) such that cells or genes below and equal to the 5th percentile were discarded. Next, cells with more than 10% of their gene expression assigned to mitochondrial genes were discarded as these cells are likely to be undergoing apoptosis. Five sex-associated DEGs (*Xist, Ddx3y, Eif2s3y, Hsp90ab1, P4ha1*) identified in the bulk RNA analysis that overlap with DEGs detected between 24 m WT (female) and 6 m WT (male) in our single-cell data were also filtered out. Cells not in the G1 phase were also removed using scores calculated from cyclone^96^. Lastly, genes must contain more than 1 count in at least 2 cells, resulting in a dataset consisting of 6685 genes by 893 cells. SCATER (version 1.6.1)^97^, SCRAN (version 1.6.6)^98^, and SINGLE CELL EXPERIMENT (version 1.0.0)^99^ were used for plotting PCAs and quality control plots^97^. Normalization was done by calculating Log_2_ counts per million (CPM). Violin plots of DAM1 and DAM2 genes were obtained using Seurat’s (version 2.3.4) VlnPlot function in R^100^.

### Feature selection

For optimization, each feature selection method (M3DROP (version 3.5.0)^101^, highly variable genes, correlation-based, PCA-based, depth-adjusted negative binomial (DANB)) was implemented before running SC3 (version 1.7.6)^40^. Rand index was calculated using MCLUST^102^ to quantify accuracy of the feature selection method. DANB was found to have the highest rand index of approximately 90%. The number of feature genes was ascertained by calculating rand indexes after running SC3. We found that the rand index does not increase significantly beyond the 25th percentile of genes used. Therefore, we used the top 1671 (25th percentile) of the genes as our set of feature genes, which were optimal for discriminating subpopulations of cells in our dataset.

### Clustering

Clustering was performed using the SC3 method^40^, which is based on unsupervised clustering of scRNA-seq data. The optimal number of clusters (*k*) was found using the Sc3_estimate_k function of SC3, and subsequently we set *k* = 4, achieving a rand index of approximately 91.7%. The Kruskal-Wallis test within SC3 (get_de_genes) was also used to detect DEGs across all 4 a priori labels and clusters.

### General R packages

General R packages used include ggplot2^103^, pheatmap^104^, reshape^105^, reshape2^105^, ggbeeswarm^106^, igraph^107^, readxl^108^, magrittr^109^, dplyr^110^, RColorBrewer^111^, R.utils^112^, ggrepel^113^, gridExtra^114^, ggthemes^115^, Matrix^116^, biobase^117^, matrixStats^118^, scales^119^, annotables^120^ and gplots^121^. preprocessCore^122^ was used for core preprocessing. Gene sets used for clusterProfiler enrichment analysis were derived from Bader Lab^123^. Annotation of human genes was done using org.Hs.eg.db package^124^.

### Differential expression and gene regulatory network analyses

For differential expression analysis, we utilized edgeR^125^(version 3.20.8) via the edgeRQLF function for pairwise differential expression analysis across two cell populations (i.e., between 2 a priori labels or 2 SC3-derived clusters), and size factors were calculated using computeSumFactors() from SCRAN. Multiple testing correction was implemented using the Benjamini & Hochberg (BH) correction and significant DEGs were called at the BH-adjusted *p*-value < 5% threshold. The top 50 DEGs across the 4 groups in Fig. 2g were plotted using SC3’s get_de_genes(), which uses a non-parametric Kruskal-Wallis test.

For regulon identification, gene regulatory network analysis was performed using SCENIC method (version 0.1.7)^49^. SCENIC integrates a random forest classifier (GENIE3)^49^ (version 1.0.0) to identify potential TF targets based on their co-expression with RcisTarget^49^ (version 0.99.0) for *cis*-regulatory motif enrichment analysis in the promoter of target genes (±500 bp of the transcription start site (TSS)) and identify the regulon, which consists of a TF and its co-expressed target genes. The *Mus musculus* 9 (mm9) motif database provided by the SCENIC authors was used. Finally, for each regulon, SCENIC uses the AUCell^49^ (version 0.99.5) algorithm to score the regulon activity in each cell. The input for SCENIC was the 6685 (genes) by 893 (cells) matrix obtained after filtering, as detailed above, and gene expression is reported in Log_2_ CPM units. Unlike in the original SCENIC pipeline, we did not implement the 2-step filtering as suggested because the input matrix was already filtered using our own criteria. Otherwise, all parameters used for running were specified in the original SCENIC pipeline. The regulon activity matrix was binarized (giving 1/0 activity score for each cell) and the heatmap of the hierarchical clustering of the binarized matrix was plotted upon removing TFs with less than 100 genes (as these identified regulons are sporadically expressed in the binary heatmap, and not clearly separated compared to larger regulons). In addition, we focused only on regulons that are active in more than 10% of the cells (Fig. 4e). For Supplementary Data 7, each TF’s regulon activity refers to the AUC (area under curve) scores from AUCell step in the SCENIC pipeline. Briefly, for each cell, each regulon’s AUC represents both the fraction of regulon genes expressed in the cell and the expression levels of these genes with respect to non-regulon genes. For each regulon, we normalize the regulon activity based on the maximum regulon activity across all cells. Lastly, we calculate the mean of the normalized regulon activity for each a priori cell-type group (i.e., 6 M WT, 24 M WT, 6 M 5xFAD XO4^−^ and 6 M 5xFAD XO4^+^). For the regulon analysis in Supplementary Fig. 10c, we opted to employ the python version of SCENIC (pySCENIC version 0.10.3)^50^ for the larger dataset because pySCENIC is computationally more efficient and scalable. Importantly, we note that pySCENIC is able to recapitulate results obtained from R version of SCENIC. Ranking databases used were mm9-500bp-upstream-7species.mc9nr.feather and mm9-tss-centred-10kb-7species.mc9nr.feather which were downloaded from https://resources.aertslab.org/cistarget/. The motif database was downloaded from https://resources.aertslab.org/cistarget/motif2tf/. Resulting output was binarized using pySCENIC’s binarize() function. The row means of the binarized score were calculated, thus representing the proportion of cells in each group that is activated for the regulon. The top 20 regulons in terms of variance across all cell-type groups are visualized.

### Pseudotime analysis

We used diffusion map in the destiny R package (version 2.6.1) for the pseudotime analysis^126^. Specifically, for the phagocytosing pseudotime, we used the list of 536 DEGs between 6 m 5xFAD XO4^−^ and 6 m 5xFAD XO4^+^ cells (FDR < 0.05). For ageing, we used 104 DEGs between 6 m WT and 24 m WT cells (FDR < 0.05). In order to plot the pseudotime, phagocytosis-specific and ageing-specific genes were defined as the non-overlapping genes between phagocytosis and ageing. This resulted in 474 phagocytosis genes and 42 ageing genes for diffusion map calculation. For defining pseudotime order, cells were ranked based on the first component of the diffusion map. For Supplementary Fig. 7a, b, we plotted the top 20 ageing-specific and top 20 phagocytosis-specific genes (based on absolute LFC) ordered by their respective pseudotime. Another pseudotime algorithm Slingshot (v1.4.0) was performed to recapitulate the results. Lineages were constructed based on PCA dimension reduction and the 4 a priori groups: 6 M WT, 24 M WT, 6 M 5xFAD XO4^−^ and 6 M 5xFAD XO4^+^. Similarly, cells were ranked based on the generated pseudotime order.

### Combined analysis of aged mouse microglia

Single-cell RNA-sequencing was performed on 3 CX3CR1^GFP^ (WT for AD mutations) mice of different ages (6, 9, and 12 m) using 10X Genomics (v3.1.0) and mm10 (v3.0.0) as the transcriptome. The minimum number of cells needed for a gene to be detected was set at 20. For each murine single-cell dataset, preprocessing was done with the following criteria: <200 number of detected features <4000, percentage mitochondrial genes <10%, and cells not in G1 phase were removed. Seurat’s NormalizeData() was used for log normalization; FindVariableFeatures() was used to find highly variable genes with the vst method and number of features set at 5000, while data were scaled using ScaleData(). RunPCA() and RunUMAP() were performed using 50 dimensions. Clustering was set at a resolution of 0.6. Subsequently, the 3 processed mouse datasets were merged using MergeData() before undergoing the same preprocessing steps above. scds (v1.2.0)^127^ was used to calculate the cxds score for identifying doublets. Cells with cxds score of more than 1.0 were removed and further filtering of number of counts <5000 and number of features <2000 was implemented to remove outlier clusters, giving us a final expression matrix of 12,604 genes by 3259 cells.

In order to combine the microglia datasets of different ages, Seurat3 integration was performed between our initial single-cell mouse dataset (6685 genes by 893 cells) and the downsampled, aged microglial dataset, where each time point was downsampled by taking 200 random cells yielding an expression matrix of (12,604 genes and 600 cells). This was followed by Slingshot (v1.4.0) pseudotime analysis where start.clus was defined as 6 m WT (from initial dataset of 6685 genes by 893 cells) and end.clus was defined as 24 m WT. Similarly, the Slingshot trajectory with 6 m WT as starting cluster and 24 m WT as ending cluster was taken to be the ageing trajectory. We next ranked the cells according to the generated pseudotime. This whole process—from integration to ranking—was performed iteratively 1000 times and the median rank for each group was noted for each iteration. The final pseudotime generated was based on the distribution of median ranks generated from this iterative process.

### Comparison with mouse microglia datasets

In all, 4 mice signatures were defined from downloaded datasets: AD vs WT (282 genes, adjusted *p*-value < 0.05, Zhou et al.)^51^, AD vs Trem2KO AD (166 genes, adjusted *p*-value < 0.05, Zhou et al.), DAM vs homeostatic (1176 genes, FDR < 0.05, Keren-Shaul et al.)^13^ and DAM2 vs DAM1 (244 genes, FDR < 0.05 and absolute (LFC) > 0.25, Keren-Shaul et al.). GO analysis was performed using clusterProfiler (v3.14.3) and DOSE (v3.12.0)^128^. For Zhou et al. mice data (GSE140511) used for pySCENIC regulon analysis, preprocessing was performed using the following criteria: percentage mitochondrial genes <5%, 300< number of detected features <5600, and 300< number of UMIs <9000. Seurat (v3.1.5) was used for normalization using the LogNormalize method and scale factor of 10,000. FindVariableFeatures was performed using the vst method and number of features set at 5000, while ScaleData was used for scaling. Dimension reduction was performed using RunPCA() and RunUMAP() with the number of dimensions set at 50. Cell types for Zhou et al. were defined using markers from the R package, BRETIGEA (Brain Cell Type Specific Gene Expression Analysis)^129^. For cluster identification of microglia, FindNeighbors and FindClusters were used with 50 PCA dimensions and resolution set at 0.4. Functional enrichment analysis was done using clusterProfiler.

### Comparison with human microglia datasets

In all, 4 human microglia datasets were downloaded: Leng et al.^54^ (Synapse: 10.7303/syn21788402; only data from entorhinal cortex was used for analysis), Zhou et al.^51^ (Synapse: 10.7303/syn21125841), Mathys et al.^52^ (https://www.synapse.org/#!Synapse:syn18485175), and Grubman et al.^53^ (GSE138852). Seurat was used to normalize and scale each dataset. Normalization was performed using the LogNormalize method and scale factor of 10,000. FindVariableFeatures was performed using the vst method and number of features set at 5000. Dimension reduction was performed using RunPCA() and RunUMAP() with the number of dimensions set at 50. For Leng et al., classification of Alzheimer’s Disease (AD) state was as: No AD (Braak Stage 0), Mild AD (Braak Stage 2) and AD (Braak Stage 6). For Mathys et al. and Zhou et al., classification of AD status was as: AD (clinical cognitive diagnosis/dcfdx = 4 or 5), Mild AD (dcfdx = 2 or 3) and No AD (dcfdx = 0 or 1). Note that a dcfdx of 2 or 3 corresponds to MCI. For Zhou et al., preprocessing of data was performed with cut-offs of percentage mitochondrial genes <5%, 400< number of detected features <7000, and 400< number of UMIs <20,000. Cell types for Zhou et al. were defined using markers from BRETIGEA^129^. Integration of all 4 datasets was performed using Seurat3 integration approach. FindIntegrationAnchors() was performed using 50 dimensions and 5000 anchor features. Subsequent PCA and UMAP dimension reduction were performed using 30 dimensions. Clustering was done using FindNeighbors() on 30 PCA dimensions while resolution for FindClusters() was set at 1.0. For the mouse signature score calculation, 5 DEG sets: XO4^+^ vs XO4^−^ (our study), AD vs WT^51^, AD vs Trem2KO AD^51^, DAM vs homeostatic^13^ and DAM2 vs DAM1^13^ were defined as mentioned in previous sections. Mouse genes were converted to human genes using biomaRt^130^ (8th July 2020) and only genes with positive fold change were retained, before Seurat’s AddModuleScore() was performed on the integrated assay to derive the signature scores. For differential expression, FindAllMarkers() was used on the RNA assay with both min.pct and LFC threshold set at 0.25. For the proportion dot plots, each proportion was calculated with respect to the separate datasets and clusters. The proportion is defined as the number of patients with cells present in the respective cluster divided by the total number of patients with similar AD status. A high proportion indicates that the cluster has a substantial number of patients represented in that particular AD status. The percentage is calculated as: (1) For each patient, we calculate the percentage of the patient’s cells expressed in each cluster. (2) The final percentage is the median of all the previously derived percentages of patients with cells expressed in that cluster. A high percentage indicates that, for patients with cells represented in that cluster, there is a high percentage of their cells present in the cluster. Boxplot statistics were calculated using the package ggsignif (version 0.6.0)^131^, rstatix (version 0.6.0)^132^ and ggpubr (version 0.4.0)^133^. Note that 1 patient (10102206) from Mathys et al. was removed from the proportion analysis because of its dcdfx of 6 which was classified as Other Dementia. Functional enrichment analysis was done using clusterProfiler.

### Pruning of regulons

For the *Hif1a* regulon (*n* = 1122 genes), we further pruned the regulon size as follows: first, we overlapped the genes in the regulon with the 1671 feature genes resulting in common set of 371 genes; second, we derived the DEGs from the set using the Kruskal-Wallis test via SC3’s get_de_genes() across all 4 a priori clusters (adjusted *p*-value = 0.05), which resulted in 203 genes. Note that we also applied the same methodology to prune *Elf3* regulon (*n* = 670 genes), yielding 106 genes.

### Comparison with Wendeln et al

The red module containing *Hif1a* (*n* = 949 genes) was taken from Wendeln et al.^65^ for comparison with our pruned *Hif1a* regulon (*n* = 203 genes).  The hypergeometric test was used to calculate the significance of the overlap (*p*-value).

### Projection analysis

In order to determine the relation between our bulk RNA-seq and scRNA-seq data, we projected our single-cell data onto the bulk using flashClust (version 1.1.2)^134^ and reference component analysis (RCA)^135^. Input units were in Log_2_CPM value, and no additional normalization or transformation was performed. Briefly, the expression profile of each single cell was projected onto each sample in the bulk RNA-seq data by calculating the Pearson correlation coefficient between the log_2_ (CPM) vector from scRNA–seq and bulk RNA-seq. For each cell, the Pearson correlation coefficients were *z*-score-transformed and grouped by hierarchical clustering of the bulk RNA-seq data. The results of the projection analysis are reported in Supplementary Fig. 14d.

### Ingenuity pathway analysis

To find upstream regulators of the regulons identified by SCENIC, we implemented IPA^55^ (27th June 2018). For *Hif1a*, we used the pruned set of 203 genes as input for IPA (five genes: *Gltscr2, Wbp5, Amica1, Myeov2* and *0610011F06Rik* were not present in the IPA database) and their respective fold changes; here, we used the log_2_ fold changes derived from the 5xFAD XO4^+^ vs 5xFAD XO4^−^ comparison. Next, we used IPA to predict the upstream regulators of the *Hif1a* regulon. As a first step, we extracted the regulators from the top five Regulator Effects’ networks robustly inferred by IPA (consistency score > 10) from the *Hif1a* regulon gene set. In doing this, we required the *Hif1a* gene to be included in the set as a direct downstream target of the regulated network. All upstream regulators must also have an absolute activation *z*-score higher than 2. We also required the regulated network to have a significant overlap with the *Hif1a* regulon gene set (*p* < 0.05). The top ten upstream activators are reported in (Fig. 6a) and are ranked by their potential activation (*z*-score); the complete list of predicted activators is presented in Supplementary Data 9. The cartoon diagram of the hypothesis generated by IPA was drawn using BioRender (https://app.biorender.com) under an individual license with unlimited academic publishing rights.

### In-solution tryptic digestion of proteins

Synaptosomes were prepared using the Procedure for Synaptic Protein Extraction from Neuronal Tissue and Syn-PER Synaptic Protein Extraction Reagent (Thermo Fisher, #87793, containing a half tablet of cOmplete™ Protease Inhibitor Cocktail (Roche, #CO-RO) per 12.5 ml Syn-PER reagent) from 250 mg of frozen mouse-brain tissue. Cell pellets were lysed (60 µl per 2 × 10^4^ microglial cells per sample; 150 µl per ~4 × 10^5^ microglial cells for library preparation; 500 µl for bulk synaptosomes) in 1% (w/v) sodium deoxycholate (SDC, Merck) in 100 mM Tris pH 8.1, then boiled at 95 °C for 5 min. After centrifugation, sample supernatant was subjected to alkylation by addition of 40 mM chloracetamide (CAA, Merck) and incubation for 20 min at room temperature (RT) in the dark. Samples were digested by addition of porcine trypsin (enzyme-to-protein ratio of 1:100; for 2 × 10^4^ microglial samples, 300 ng was used; Merck) and incubated overnight at 37 °C with shaking. The following day, the reaction was stopped and SDC precipitated through addition of formic acid to a final concentration of 1% (v/v). Peptides were extracted through adding an equal volume of 100% (v/v) water-saturated ethyl acetate, vortexing and centrifugation at maximum speed in a benchtop microfuge and transferring the aqueous phase to a new tube, with this entire step repeated once. Samples were then vacuum concentrated (Labconco Centrivap) and peptides purified by C_18_ ZipTips^®^ (Merck) prior to analysis by LC-MS/MS or LC-SWATH-MS. Note that all samples were supplemented with 200 fmole of each iRT peptide^136^ (Biognosys, #Ki-3002-1). For bulk synaptosome samples, peptides were further fractionated prior to LC-MS by reversed-phase HPLC using an Ettan ÄKTA micro HPLC system (GE Healthcare), as described elsewhere^137^.

### LC-MS/MS and spectral library generation

Spectral libraries were generated from mass spectrometry of tryptic peptides derived from a combination of microglia cells and synaptosomes. Purified peptides were analysed on a TripleTOF^®^ 6600 mass spectrometer (SCIEX) equipped with an on-line Eksigent Ekspert nanoLC 415 (SCIEX). Following autosampler injection, samples were subjected to trap-elution through loading onto a trap column (Eksigent nanoLC trap, #5016752; ChromXP C18, 3 µm 120 Å, 350 µm × 0.5 mm [SCIEX]) at 2 µl/min for 10 min in loading buffer (2% (v/v) acetonitrile in water supplemented with 0.1% formic acid) followed by separation at 300 nl/min across an analytical column (Eksigent nano LC column, #805-0012; ChromXP C18, 3 µm 120 Å, 75 µm × 15 cm [SCIEX]) equilibrated in 98% buffer A (0.1% (v/v) formic acid in water) and 2% (v/v) buffer B (80% (v/v) acetonitrile in water supplemented with 0.1% (v/v) formic acid) followed by increasing concentrations of buffer B. Specific gradient conditions were: increase from 2% to 10% (v/v) buffer B from 0 to 2 min, then from 10% to 40% (v/v) buffer B from 2 to 152 min, then from 40% to 50% (v/v) buffer B from 152 to 154 min, then from 50% to 99% (v/v) buffer B from 154 to 157 min, then hold at 99% (v/v) buffer B from 157 to 167 min, and then from 99% to 2% (v/v) buffer B from 167 to 168 min, followed by re-equilibration at 2% (v/v) buffer B until the end of the run. The mass spectrometer was operated in information-dependent acquisition mode using the following settings: for MS1 accumulation time of 200 ms, scan range of 300–1800 *m*/*z*; for MS2, a switch criteria was used of the top 20 precursors exceeding 40 counts with charge state from 2 to 5, rolling collision energy and with ions excluded for 30 s after two occurrences; MS2 accumulation time was set to 150 ms and with a scan range of 80–2000 *m*/*z*. Acquired spectra were searched using ProteinPilot™ v5 (SCIEX) against the complete reference mouse proteome (Uniprot, 201707 build) and the resultant search was imported into Skyline v3.7.11317^138^.

### LC-SWATH-MS

For data-independent acquisition, purified peptides were analysed on a TripleTOF^®^ 6600 mass spectrometer (SCIEX) using the same LC setup and conditions as above, with the exception that the mass spectrometer was operated in SWATH-MS^34^ mode, using the following conditions: initial MS1 scan across 400–1250 *m*/*z* with accumulation time of 150 ms, followed by 100 variable SWATH windows (calculated using the Variable Window Calculator Excel tool, downloaded from http://sciex.com/support/software-downloads) spanning a range of 400–1241 *m*/*z* with a 1 Da overlap and each with an accumulation time of 25 ms. Rolling collision energy was used, with a collision energy spread of 5.

### SWATH-MS data analysis

SWATH-MS data were analysed using Skyline v3.7.11317 against the generated microglial and synaptosome spectral library, applying predicted retention times from the included iRT peptides detected within each sample in order to aid peak picking. Peak scoring was then re-trained within Skyline following the addition of shuffled decoy peptides. Data were initially refined through accepting peptides with an absolute ppm < 10 and, for each peptide, there needed to be at least one sample of the set with a peptide dotp of >0.8; peptides not meeting these criteria were excluded. Subsequently, all remaining peptides were subjected to manual interrogation followed by exporting integrated peak areas into Microsoft Excel 2016 for further processing. The list of peptides used for signal normalization is listed in Supplementary Data 4. Peptides corresponding to 304 of the XO4^+^ covariate genes were above the limit of detection. Samples and proteins were clustered using one minus Spearman correlation, and data expressed as a heatmap of log_2_-transformed normalized fold changes compared to WT microglia using GENE-E software v3.0.215 (Broad Institute).

### Organotypic hippocampal slice cultures

Organotypic hippocampal (brain) slice cultures (OSHCs) were adapted from published protocols^139^. On day 0, brains from 6-month-old 5xFAD and WT animals were coronally sectioned through the hippocampus on a vibratome (Leica; settings: speed = 0.4 mm/s and amplitude = 1.00 mm) in ice-cold cutting medium (MEM 1x, Life Technologies) containing 10 mM Tris, 50 U/ml Penicillin and 50 µg/ml Streptomycin) to obtain 400 μm thick brain slices. Brain slices were cultured in vitro with culture medium (25% (v/v) MEM 2x, 25% (v/v) HBSS 1x (Life Technologies), 25% (v/v) Horse serum (Life Technologies) with 10 mM Tris, 25 U/ml Penicillin and 25 µg/ml Streptomycin and 0.455% (v/v) 7.5% NaHCO_3_ aqueous solution) on Millicell Cell Culture Insert (Merck) at air–medium interface. The media was completely replaced every second day. After 3 days of resting, Aβ plaques on brain slices were stained using an alternative fluorescent amyloid plaque-labelling dye, NIAD-4 (10 μM, BioVision, #2710) for 3 h, prior to the addition of ex vivo microglia-enriched fraction isolated from 6-month-old 5xFAD and WT animals. Microglia-rich fractions enriched by Percoll gradient (described in Acute isolation of microglia and fluorescence-activated cell sorting) were stained with CFSE (final concentration 5 μM; Life Technologies) for 20 min at 37 °C, and 2 × 10^4^ cells were added per hippocampus onto NIAD-4-stained hippocampal slice cultures for 5 days. As a control, synaptosome-labelled pHrodo-red particles were added to OHSCs for 5 days. Endogenous and replenished microglia were purified from OSHCs by mechanical dissociation using 70 μm mesh and enriched for microglia by density gradient centrifugation in 30% (v/v) isotonic Percoll at 1000*g* for 15 min. Cell pellets were incubated with Fc block (1:200; BD Biosciences, #553141) for 15 min on ice prior to staining with CD11b-PE (1:50) and CD45-BV786 (1:200) for 15 min. Cells were washed once in PBS and resuspended in 400 μl Zombie IR dye (1:1000; Thermo Fisher Scientific) for live cell discrimination. Endogenous (CFSE^−^) and exogenous (CFSE^+^) microglia (single, live, CD11b^+^, CD45^lo^) that were either positive or negative for NIAD4 were sorted into 96-well plates (10 cells/well) containing 10 μl of Lysis Buffer from the Single Cell to Ct kit (Thermo Fisher Scientific), using the FACSAria™ III cell sorter. As a control, pHrodo-red-containing microglia were also sorted from the slices. The sorting/gating strategy is shown in Supplementary Fig. 9a. cDNA synthesis and pre-amplification (18 cycles) were performed in accordance with manufacturer’s instructions and pre-amplified cDNA was diluted 5-fold prior to qPCR. The primers and probes used are listed in Supplementary Data 12. Three independent biological experiments were performed. If enough cells were present from any populations, additional technical replicates were sorted. We report all the data, including experimental (solid circles) and technical (open circles) replicates, when available, for these experiments in Fig. 3d–g. The percentage of sorted cells belonging to Cluster 1 or Cluster 2 is calculated based on all replicates.

### Differentiation to iMGLs

iHPC Differentiation Base Medium: IMDM (50%; Thermo Fisher Scientific), F12 (50%), ITSG-X, 2% v/v, Thermo Fisher Scientific), L-ascorbic acid 2-Phosphate magnesium (64 μg/ml; Sigma), monothioglycerol (400 μM; Sigma), PVA (10 μg/ml; Sigma), Glutamax (1x; Thermo Fisher Scientific), chemically defined lipid concentrate (1x; Thermo Fisher Scientific), non-essential amino acids (NEAA; 1x; Thermo Fisher Scientific), Penicillin/Streptomycin (P/S; 1% V/V; Thermo Fisher Scientific). Use 0.22 μm filter.

iMGL Differentiation Medium: phenol-free DMEM/F12 (1:1), ITS-G, 2%v/v, B27 (2% v/v), N2 (0.5%, v/v), monothioglycerol (200 μM), Glutamax (1x), NEAA (1x), and additional insulin (5 μg/ml; Sigma), filtered through a 0.22 μm filter; supplemented with M-CSF (25 ng/ml; Miltenyi Biotec), IL-34 (100 ng/ml; Miltenyi Biotec), and TGFβ-1 (50 ng/ml; Miltenyi Biotec) and cholesterol (1.5 μg/ml; Avanti Polar Lipids^140^).

The protocol for iMGL derivation was adapted from^59^ with modifications from the StemDiff Hematopoietic Kit (Stem Cell Technologies, #05310), as we recently described^60^ and similar to^141^. H9 CX3CR1-TdTomato cells were cultured on vitronectin (Thermo Fisher Scientific, #A14700)-coated T25 flask (Sarstedt, #83.3910.002) in E8 medium (Thermo Fisher Scientific, #A1517001). Two days prior to differentiation, cells were detached in 0.5 mM EDTA and 40–80 colonies/well were seeded into a 12-well plate in E8 medium. On day 0, E8 medium was exchanged for 1 ml of supplemented iHPC Differentiation Base Medium per well. iHPC Differentiation Base Medium supplemented with FGF2 (50 ng/ml), BMP4 (50 ng/ml), Activin-A (12.5 ng/ml), ROCKi (1 μM) and LiCl (2 mM), and incubated in a hypoxic incubator. On day 2, medium was changed to 1 ml of iHPC Differentiation Base Medium supplemented with FGF2 (50 ng/ml) and VEGF (50 ng/ml), and incubated in a hypoxic incubator. On day 4, medium was changed to 1 ml iHPC Differentiation Base Medium containing FGF2 (50 ng/ml), VEGF (50 ng/ml), TPO (50 ng/ml), SCF (10 ng/ml), IL-6 (50 ng/ml) and IL-3 (10 ng/ml), and incubated under normoxia. Half the medium was replaced on days 5 and 7. On day 10, the supernatant containing the HPCs was collected, centrifuged (300*g* for 5 min at RT), then 0.5 ml cell-containing media was replaced and supplemented with 0.5 ml fresh media. On day 12, the supernatant containing HPCs was collected and plated onto Matrigel (1:100; hESC-qualified Matrix, LDEV-Free, Falcon, #354277)-coated 12-well plates at 4 × 10^4^ cells per well in iMGL complete differentiation medium. Every 2 days, each well was supplemented with 0.5 ml per well of complete differentiation medium, and at day 22 a 50% media change was performed. iMGL experiments were performed at days 22–23 of differentiation. iMGLs were stimulated with human BMP9 (20 ng/ml; Biolegend), Pam3csk (100 ng/ml; Invivogen), Poly:IC (1000 ng/ml; Invivogen) and/or rapamycin (5 nM; Sigma) for 24 h prior to RNA isolation or collection of culture supernatant.

### Comparison with gene expression perturbed by small molecules

We consider the DEGs upregulated by TLR1-agonist administration but downregulated by rapamycin as determined using Voom/Limma functions in degust (http://degust.erc.monash.edu, and FDR < 0.05, LFC > 1). We compared the LFC of LY86, CTSB, HIF1A, LDLR, CD63, CTSL and CTSA in this dataset with those in XO4^−^ vs 6 M WT and XO4^+^ vs 6 M WT DE analysis. In addition, for Supplementary Fig. 13c, we compared 866 DEGs upregulated by TLR1-agonist Pam3csk and downregulated by rapamycin with 1092 DEGs from the bulk RNA DE analysis between XO4^+^ and XO4^−^ genes (FDR < 0.05, LFC > 1). We subsequently plotted the overlap (65 genes) between these 2 sets of analysis to represent genes which are differentially expressed in both XO4^+^ microglia (vs XO4^−^ microglia) and upregulated by Pam3csk administration but downregulated by rapamycin. To calculate the *p*-value, we use a hypergeometric test where the population is taken to be the 4319 DEGs from the bulk RNA DE analysis between XO4^+^ and XO4^−^ genes (LFC > 1). This set of genes has 92 genes overlapping with the 866 DEGs upregulated by Pam3csk and downregulated by rapamycin. The sample taken for the hypergeometric test is the 1092 DEGs which has 65 genes overlapping with the 866 DEGs upregulated by Pam3csk and downregulated by rapamycin. Overall, phyper formula used is phyper(65-1,92,4319-92,1092,lower.tail = F). bioMart was used to convert all mice genes to their human orthologues. Functional enrichment analysis was done using clusterProfiler.

### Immunofluorescence staining

Right brain hemispheres from methoxy-XO4-injected PBS-perfused mice were fixed in 4% PFA overnight, followed by immersion in 30% (w/v) sucrose solution for 48 h and frozen with liquid N_2_. Samples were stored at −80 °C prior to sectioning. Frozen hemispheres were cryostat-sectioned into 60 or 20 μm thick sections onto slides for histological staining. Sections were blocked for 1.5 h in PBST (containing 2% (w/v) BSA and 0.5% (v/v) Triton-X) followed by 1 h incubation with 0.5% (v/v) Mouse on Mouse (M.O.M.^™^) Blocking Reagent (Vector Laboratories, #MKB-2213-1). Sections were then stained with primary antibodies, including the rabbit anti-Iba-1 (1:500, #019-19741, Wako, Virginia, USA), mouse anti-PSD95 (1:160, #MAB1596, Merck Millipore), mouse anti-6E10 (1:500, #803001, Biolegend), rat anti-CD68 (1:100, MCA1957, Biorad), overnight at RT followed by Alexa Fluor 488 goat anti-rabbit IgG (H + L) (1:250, #A11008, Life Technologies), Alexa Fluor 635 goat anti-mouse IgG (H + L) (1:250, #A31575, Life Technologies) or Alexa Fluor 568 donkey anti-mouse IgG (H + L) (1:500, #A10037, Life Technologies) or Alexa Fluor 488 goat anti-rat IgG (1:800, #A11006, Life Technologies), respectively, for 2 h at RT. Sections were then mounted with Mowiol mounting medium. For 6E10 staining, paraffin-embedded human brain sections were de-waxed for antigen retrieval with 90% formic acid (5 min), followed by citrate boiling (45 min, 98 °C DAKO Citrate Buffer, DAKO PT Link). All sections were then blocked using 0.1% (w/v) Sudan Black B in 70% ethanol (5 min) followed by PBST (containing 2% (w/v) BSA and 0.5% (v/v) Triton-X; 1 h). Sections were stained with primary antibodies, including the rabbit anti-Iba-1 (1:500, #019-19741, Wako), goat anti-PSD95 (1:50, # ab12093, Abcam), mouse anti-6E10 (1:500, #803001, Biolegend), rabbit anti-SPP1 (1:100, #ab8448, Abcam), rabbit anti-HIF1A (1:NB100-479, Novus Biologicals), rabbit anti-DAP12 (1:250, #ab124834, Abcam) overnight at RT followed by Alexa Fluor 488 goat anti-rabbit IgG (H + L) (1:500, #A11008, Life Technologies), Alexa Fluor 647 donkey anti-goat IgG (H + L) (1:500, #A21447, Life Technologies) or Alexa Fluor 568 donkey anti-mouse IgG (H + L) (1:500, #A10037, Life Technologies), respectively, for 2 h at RT. Alternatively to 6E10, sections were taken from PBS into a 100 μM solution of methoxy-X04 in (40% ethanol) (adjusted to pH 10 with 0.1 N NaOH) for 10 min, then the sections were dipped briefly 5 times into tap water before differentiation in 0.2% NaOH in 80% ethanol for 2 min. Sections were dipped briefly a few times into tap water, prior to washing with PBS. Sections were then mounted with Mowiol mounting medium.

### Confocal imaging and image analysis

Mouse sections were imaged on a Leica SP8 confocal microscope using a 63x oil 1.4 NA objective and 2x zoom with 1024 × 1024 resolution, resulting in a pixel size of 90 nm. Then, 30–40 μm *z*-stacks were acquired with a 0.3 μm *z*-step size, using sequential scans with 3x averaging at 488 and 647 nm wavelengths and 1x averaging at 405 nm wavelength. For human brain sections, microglia were analysed from four 4.5–5 μm *z*-stacks per patient, obtained at 63x, 1x zoom, 2048 × 2048 resolution. For quantitation of PSD95 or GAD65 engulfed by microglia or C3 co-localization with microglia, 3D rendering of confocal images was performed using surfaces, spots and cell functions and batch analysis in Imaris (v8.3.1). XO4^+^ microglia were defined as IMARIS-rendered surfaces in the Iba1 channel with mean fluorescence intensity above the threshold in the methoxy-XO4 channel. Experimenters were blinded to the genotype during image acquisition and processing.

### qRT-PCR

qPCR was performed using the Biomark Fluidigm 96.96 protocol. Briefly, assays (Supplementary Data 12) and samples were combined in a 96.96 Dynamic array IFC according to Fluidigm^®^ 96.96 Real-Time PCR Workflow Quick Reference PN 6800088. Here, 5 μl of each assay at a final concentration of 10x was added to each assay inlet port and 5 μl of diluted sample was added to each sample inlet port according to the ChipPipetting Map. The data were analysed with Fluidigm Real-Time PCR analysis software (V4.1.2). The limit of detection was set to 30. Samples with a Ct value for *Actb* (Mm00607939_s1) outside the 15–25 range were excluded from further analyses. log_2_-transformed ΔCt values for the 42 detected genes were used for clustering by SC3 and relationships between samples were visualized by SPRING (https://kleintools.hms.harvard.edu/tools/springViewer), which uses a *k*-nearest-neighbour graph rendered using a force-directed layout^142^. Plots were generated using the ggplot2 function in R. For validation of *HIF1A* regulon in human ES-derived iMGLs or the mouse BV2 microglial cell line, 400 ng RNA was reverse transcribed using SuperScript™ II Reverse Transcriptase (Thermo Fisher Scientific, #18064014). qPCR was performed using TaqMan assays (*SPP1*, Hs00959010_m1; *HIF1A*, Hs00153153_m1; *GAPDH*, Hs02758991_g1; *APOE*, Hs00171168_m1; *TREM2*, Hs00219132_m1; *CX3CR1*, Hs01922583_s1; housekeeping gene *SNRPD3*, Hs00188207_m1, mouse TaqMan assays *Apoe*, Mm00437573_m1; *Spp1*, Mm00436767_m1; *Trem2*, Mm04209424_g1; *Ctsa*, Mm00447197_m1; *Igf1*, Mm00439560_m1; *P2ry12*, mM00446026_M1; *Hif1a*, Mm00468869_m1; *Actb*, Mm00607939_s1; *Tyrobp*, Mm00449152_m1) in a Roche LightCycler^®^ 480 (Roche). We represented the changes in gene expression of several genes following the knockdown of *mHIF1a* in the form of a heatmap. Since different genes have different ranges of ΔCT values, we scaled the gene expression in a gene-wise manner, specifically by subtracting the value against the mean, followed by dividing against the standard deviation. To test the difference between different conditions and mCherry DMSO (baseline) is significant, two-sample *t*-tests were performed for each gene and the *p*-values were subsequently combined using sum of logs method, also known as Fisher’s method, via the sumlog function in the metap package (v1.4)^143^.

### Cytometric bead array

Cytometric bead array (CBA) was carried out using the BD (New Jersey, USA) CBA human flexi kit using a protocol modified from the manufacturer’s protocol. Here, 5 μl of each standard (highest concentration at 5000 pg/ml in assay diluent) and sample were incubated with 5 μl of capture bead mix (containing 0.1 μl of each cytokine Capture Bead diluted in Capture Bead Diluent) for 1 h in a 96-well V-bottom assay plate. This was followed by the addition and incubation with detection reagent mix (containing 0.1 μl of each cytokine PE Reagent diluted in Detection Reagent Diluent for 1 h in the dark). Each well was then washed once with 200 μl of Wash Buffer, and beads were resuspended in 80 μl of Wash Buffer for analysis by FACS using the LSRFortessa X-20 (BD Biosciences). At least 200 single-bead events from each cytokine population were collected. Results obtained were analysed using the FCAP Array Software Version 3.0 (BD).

### Human primary microglia culture and transfection

Primary human microglia isolated from cortex (Celprogen, #37089-01) were cultured on Matrigel-coated 12-well plates at 5 × 10^5^ cells per well in iMGL differentiation media at 37 °C in 5% CO_2_. Cells (up to passage 5) were transfected for 48 h with dox-inducible GFP-tagged Gateway-generated Piggybac expression constructs with inserted cDNA encoding human *HIF1A* and/or *ELF3* open reading frames using Glial Mag transfection kit (Oz Biosciences, #GL00500) according to manufacturer’s instructions. Primary microglia were co-transfected with plasmids encoding Hybase transposase and *rtta* at a ratio of 1:2:2 (Hybase: rtta: ORF).

### Preparation and treatment with fibrils

Aβ_1–42_ (Bachem, #4014447.5000) monomers were prepared by HFIP solubilization and aliquots were stored at −20 °C over desiccant prior to use. For preparation of aggregated fibrils, Aβ_1–42_ was freshly resuspended in 5 mM in DMSO at RT and diluted to 100 μM final Aβ with 10 mM HCl and 150 mM NaCl at RT made in 18 MΩ sterile water. Following 15 s vortex, Aβ was incubated at 37 °C for 24 h; 200 nM Aβ fibrils were added to iMGLs for 48 h.

### Fluorescent labelling of Aβ fibrils

Fibrillar Aβ (fAβ) was labelled with pHrodo™ Green STP ester using protocol adapted from Fujifilm/Cellular Dynamics Labelling Amyloid Beta with pHrodo Red protocol. First, 100 μl of fAβ was centrifuged at 16,000*g* for 2 min then diluted to 200 μl with 0.2 M Na_2_CO_3_(aq) and centrifuged at 16,000*g* for 1 min to collect the aggregates. Supernatant was removed and fAβ pellet was rinsed with 200 μl HBSS by pipetting to mix. fAβ was then pelleted by centrifugation at 16,000*g* for 1 min. Following the removal of supernatant, fAβ pellet was resuspended in 200 μl 0.1 M Na_2_CO_3_(aq) by pipetting. The solution was then added to 1.6 μl of 8.9 mM pHrodo^TM^ Green stock (prepared according to Thermo Fisher Scientific, #P35369 protocol) or 0.45 μl of 12 mg/ml Atto 647 N NHS ester (1 vial of 0.24 mg Atto 647 N NHS ester was dissolved in 20 μl of DMSO, #76508, Sigma) and incubated at 37 °C for 75 min. The reaction tube was centrifuged at 16,000*g* for 1 min and supernatant was removed. The pellet of stained fAβ was washed once by vortexing in 500 μl methanol. fAβ was pelleted by centrifugation at 16,000*g* for 1 min and resuspended by pipetting and vortex in 100 μl HBSS. pHrodo^TM^-Green-labelled fAβ was added to BV2 cells at 1:100 dilution.

### Phagocytosis of pHrodo *E. coli*, synaptosomes and pHrodo™-Green-labelled fAβ

Synaptosomes were isolated from WT mouse brain tissue or human brain (obtained from Victorian Brain Bank), according to the Syn-PER Synaptic Protein Extraction Reagent (Thermo Fisher Scientific, #87793) protocol. The protein concentration was measured by nanodrop, and synaptosomes were labelled with pHrodo™ Red succinimidyl ester (Thermo Fisher Scientific, #P36600) as described in^144^ or with blue fluorescent 2.0 µm FluoSpheres™ Carboxylate-Modified Microspheres (Life Technologies, F8824) according to manufacturer instructions. Mouse pHrodo-conjugated synaptosomes were resuspended at 3.5 μg/μl in 5% DMSO in PBS and stored at −80 °C until use and human pHrodo-conjugated synaptosomes were resuspended at 5.5 μg/μl in 1% (w/v) BSA and stored at 4 °C until use. To examine the phagocytic properties of the XO4^+^ and XO4^−^ microglia populations, the microglia-enriched cell suspensions were isolated from methoxy-XO4-injected mice as described above, stained for CD45 and/or CD11b and seeded in 96-well plates. Following 30 min of resting at 37 °C and 5% CO_2_, microglia were incubated with pHrodo-conjugated synaptosomes (4.25 μg per well), pHrodo™ Green *E. coli* BioParticles™ Conjugate (Thermo Fisher Scientific, #P35361, 66.7 ng per well) or pHrodo^TM^-Green-labelled fAβ. Cells were collected after 45 min incubation at 37 °C and 5% CO_2_ and stained with antibodies to microglia cell surface markers (CD11b-PE-Cy7, 1:200, Biolegend, #101216; CD45-APC-Cy7, 1:200, Tonbo Biosciences, #25-0459-T100). XO4^+^ and XO4^−^ microglia uptake of pHrodo-conjugated synaptosomes, pHrodo™ Green *E. coli* BioParticles™ Conjugate, or pHrodo^TM^-Green-labelled fAβ was analysed using the BD™ LSR II analyser. iMGLs, BV2 cells or primary human microglia were incubated with conjugated synaptosomes (3.44 μg/μl) for 1.5 h, and uptake of synaptosomes was analysed using the BD™ LSR II analyser or during sorting on the BD FACSAria^TM^ Fusion or BD Influx^TM^ Cell Sorter.

### Murine microglial cell line BV2 culture, transduction and phagocytosis

The murine microglial cell line BV2 was kindly provided as a gift from Prof. Peter Crack (The University of Melbourne, Australia). BV2 cells were cultured in DMEM media supplemented with 1x GlutaMAX^TM^-I, 5% (v/v) FBS, 50 U/ml Penicillin and 50 µg/ml Streptomycin. BV2 cells were seeded in 24-well plates at 10,000 cells per well and transduced for 48 h with mCherry expressing lentivirus with constitutively expressed shRNA directed against mouse *Hif1a* (pLV[shRNA]-mCherry-U6 > mHif1a[shRNA#1], VectorBuilder) or mCherry control lentivirus. Stably transduced BV2 cells were sorted for equivalent levels of mCherry expression, seeded in 12-well plates at 5000 cells per well and rested for 24 h prior to treatment with AF488-labelled Aβ fibrils for 24 h. qPCR was used to confirm knockdown of *Hif1a* and activation of *Hif1a* regulon genes in FACS-sorted cells that had phagocytosed pHrodo-labelled fAβ. The FACS gating/sorting strategy is in Supplementary Fig. 10d.

BV2 cells were transduced with dox-inducible mCherry-tagged lentiviral *Hif1a* overexpression constructs (pLV[Exp] -mCherry-TRE > mHif1a[NM_001313919.1]) and constitutive *rtta-GFP*. Briefly, BV2 were seeded in 12-well plates at 20,000 cells per well and transduced for 48 h with MOI100 of both *mCherry-Hif1a* and *rtta-GFP* vectors in the presence of doxycycline (dox, 2 µg/ml). Stable lines were generated by first FACS-sorting mCherry^+^GFP^+^ cells, further incubation with and without dox for 2 passages followed by sorting mCherry^+^GFP^+^ and GFP^+^ cells. Cells were seeded in 12-well plates at 100,000 cells per well and rested for 24 h prior to treatment with AF647-labelled Aβ fibrils (1:100) for 24 h. Phagocytosis experiments with blue-labelled synaptosomes were performed in *shRNA.Hif1a* and *Hif1a* transduced BV2 cells, as above.

### Intracellular staining for HIF1A

Following cell treatment with AF488- or AF647-labelled Aβ fibrils, cells were washed with PBS, pulse vortexed and fixed for 30 min at RT in 100 µl of 1x fix/perm buffer (# 00-5523-00, Life Technologies). Cells were washed twice with addition of 200 µl of 1x permeabilization buffer (# 00-5523-00, Life Technologies) and centrifugation at 400*g* for 5 min at RT. Cells were stained for 30 min at RT in 30 µl of rabbit anti-HIF1A antibody (1:100, NB100-479, Novus Biologicals, diluted in permeabilization buffer), washed twice and incubated with AlexaFluor 647 donkey anti-rabbit IgG (#A31573, Life Technologies) or Pacific Blue goat anti-rabbit IgG (#P10994, Life Technologies) for 30 min at RT. Following 2 washes, stained cells were resuspended in 200 µl FACS buffer and analysed using the BD™ LSR II analyser.

### Statistics

Differences between 2 groups were compared by 2-tailed *t*-test, and for more than 2 groups, by one-way ANOVA and Tukey’s, Holm-Sidak’s or Dunnet’s post hoc tests, as appropriate. Where results from *t*-tests and ANOVA are reported, assumption of normality of the distributions was determined using the Shapiro-Wilk normality test. Alternatively, non-parametric Wilcoxon (or Mann-Whitney) and Kruskal-Wallis and Dunn’s post hoc tests were used, as indicated on a case-by-case basis. To analyse the significance of gene enrichment of sensome genes in the gene signature associated with XO4^+^ microglia, we used the hypergeometric test. The hypothesis that plaque-distal human microglia internalize more PSD95 than plaque-associated microglia was tested by paired 1-tailed one-sample *t*-test.

### Reporting summary

Further information on experimental design is available in the Nature Research Reporting Summary linked to this paper.

## Supplementary information

Supplementary Information

Description of Additional Supplementary Files

Supplementary Data 1

Supplementary Data 2

Supplementary Data 3

Supplementary Data 4

Supplementary Data 5

Supplementary Data 6

Supplementary Data 7

Supplementary Data 8

Supplementary Data 9

Supplementary Data 10

Supplementary Data 11

Supplementary Data 12

Reporting Summary

## Data Availability

Bulk RNA-seq and Single-cell-seq raw and processed data from this study are available from: https://www.ncbi.nlm.nih.gov/geo/query/acc.cgi?acc=GSE165306. Proteomics raw data are included in Supplementary Data 4. The mass spectrometry proteomics data have been deposited to the ProteomeXchange Consortium via the PRIDE partner repository with the dataset identifier PXD024731 Source data are provided with this paper.

## Source data

Source Data

## Code availability

Code is available from the authors by reasonable request. Source data are provided with this paper.
