# Supplementary material for: Transcriptional signature in microglia associated with Aβ plaque phagocytosis
Source: Nat Commun. 2021 May 21;12:3015. doi: 10.1038/s41467-021-23111-1 (PMC8140091; doi:10.1038/s41467-021-23111-1)
Supplement: Supplementary file 1 — Supplementary Information [file 41467_2021_23111_MOESM1_ESM.pdf]

Supplementary Fig 1

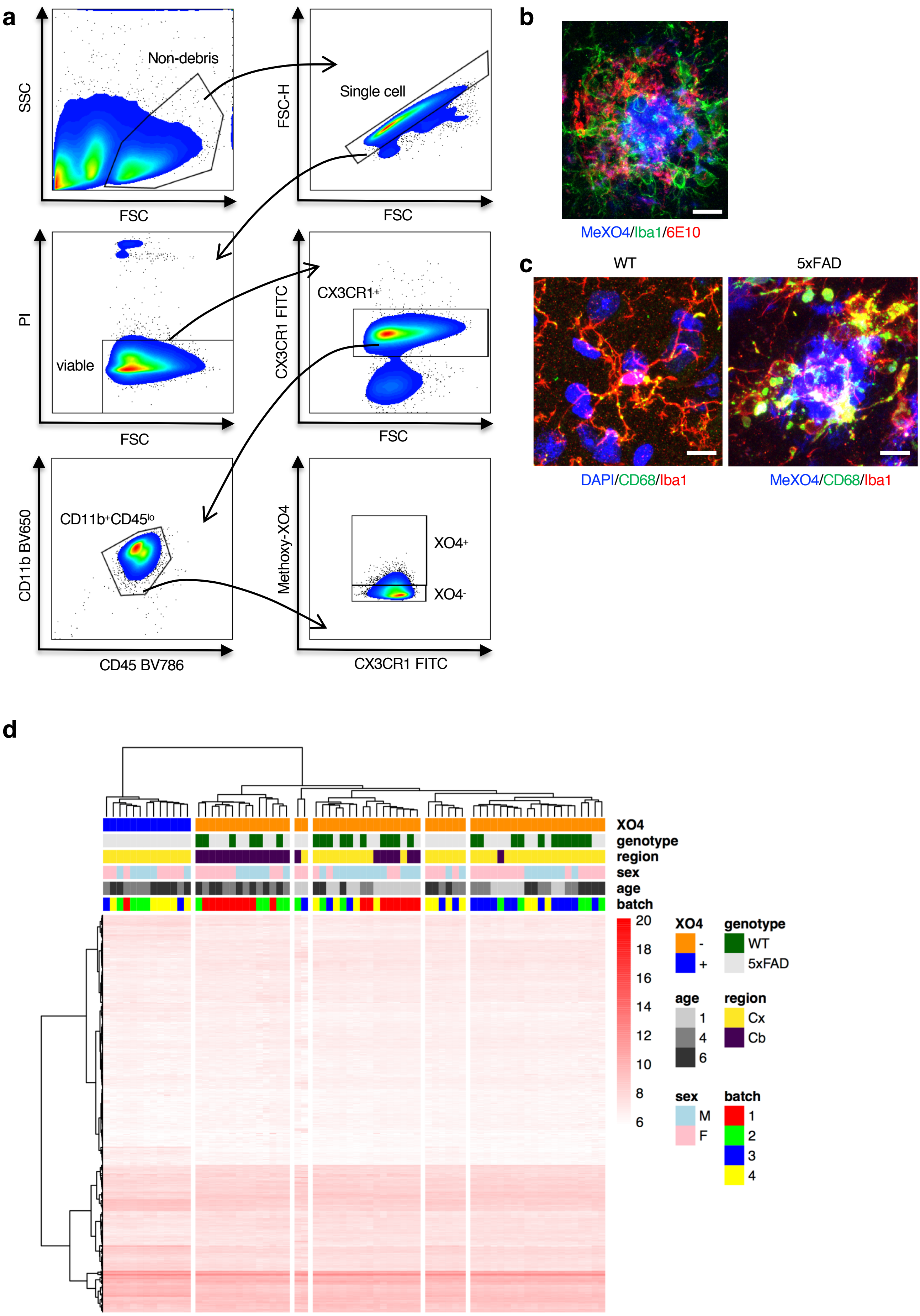

Supplementary Fig. 1a FACS gating strategy for sorting $XO4^{+}$ and $XO4^{-}$ microglia, presented in Fig. 1e, i, j and Fig. 2. **b**, Methoxy-XO4 labels the core of $6E10^{+}$ plaques, representative image from n = 6 mice. Scale: 10 µm **c,** Immunofluorescence image of brain sections from 6m WT and 5xFAD mice Methoxy XO4, the phagosome marker CD68 and microglia marker Iba1, representative from n = 5 mice per genotype, staining performed independently twice with similar results. Scale: 10 µm **d,** Hierarchical clustering using ward.D2 linkage and Euclidean distance of bulk RNA-seq data.

Supplementary Fig 2

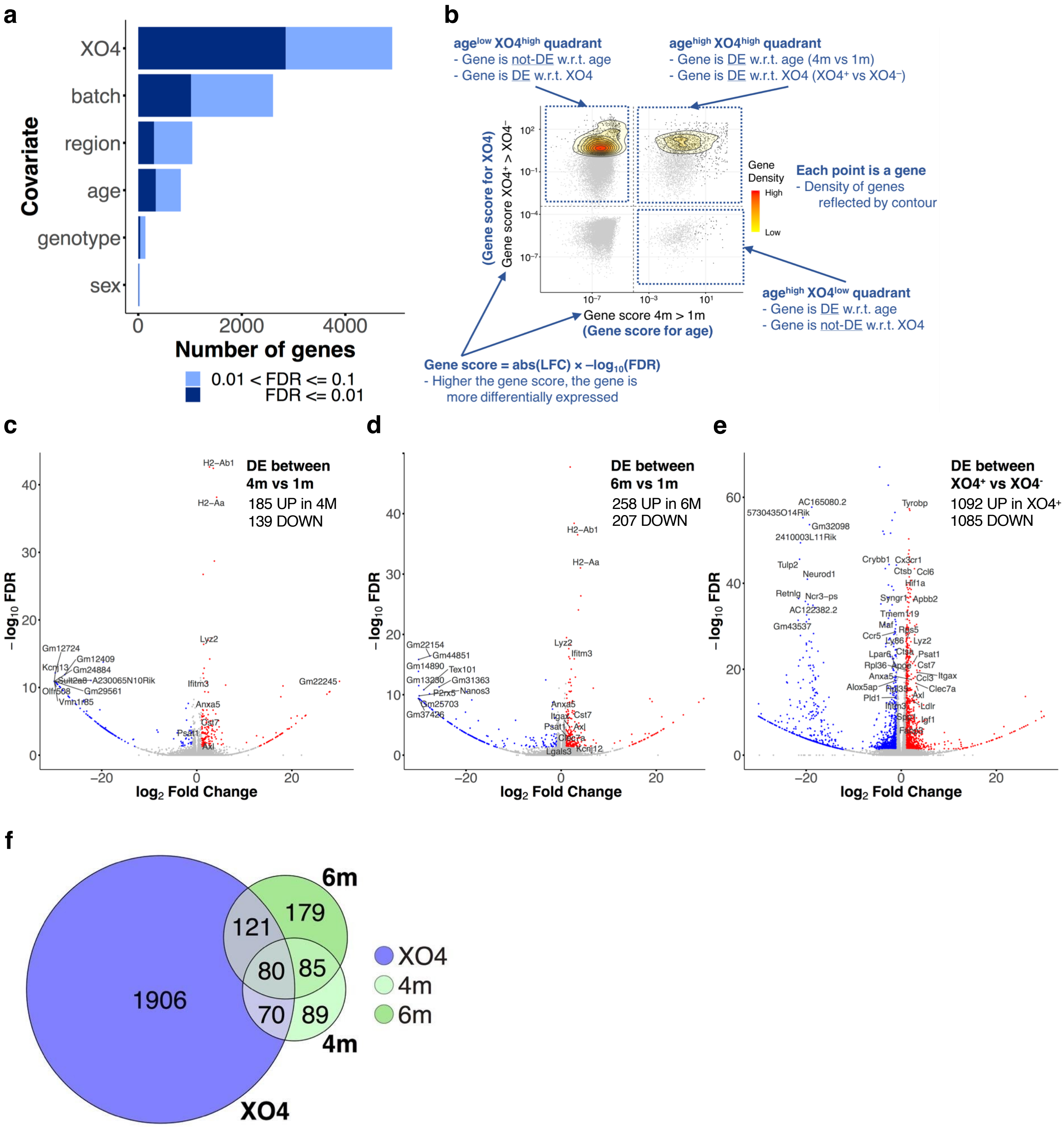

Supplementary Fig. 2a, The number of genes for which expression levels could be explained by each covariate in bulk RNA-seq data. **b,** cartoon showing how to interpret gene cytometry plots in Fig. 1f-g. **c-e,** Volcano plots representing log_{10}FDR on the y axis and log_2 Fold change on the x axis show significant gene expression changes (red, upregulated; blue, downregulated, log_{10}FDR<0.01 between **c,** 4m and 1m microglia, **d,** 6m and 1m microglia, and **e,** $XO4^-$ and $XO4^+$ microglia. **f,** Overlap between DEGs (abs(LFC)>1 and FDR<0.05) for (i) $XO4^+$ vs $XO4^-$, (ii) 4m vs 1m and (iii) 6m vs 1m.

Supplementary Fig 3

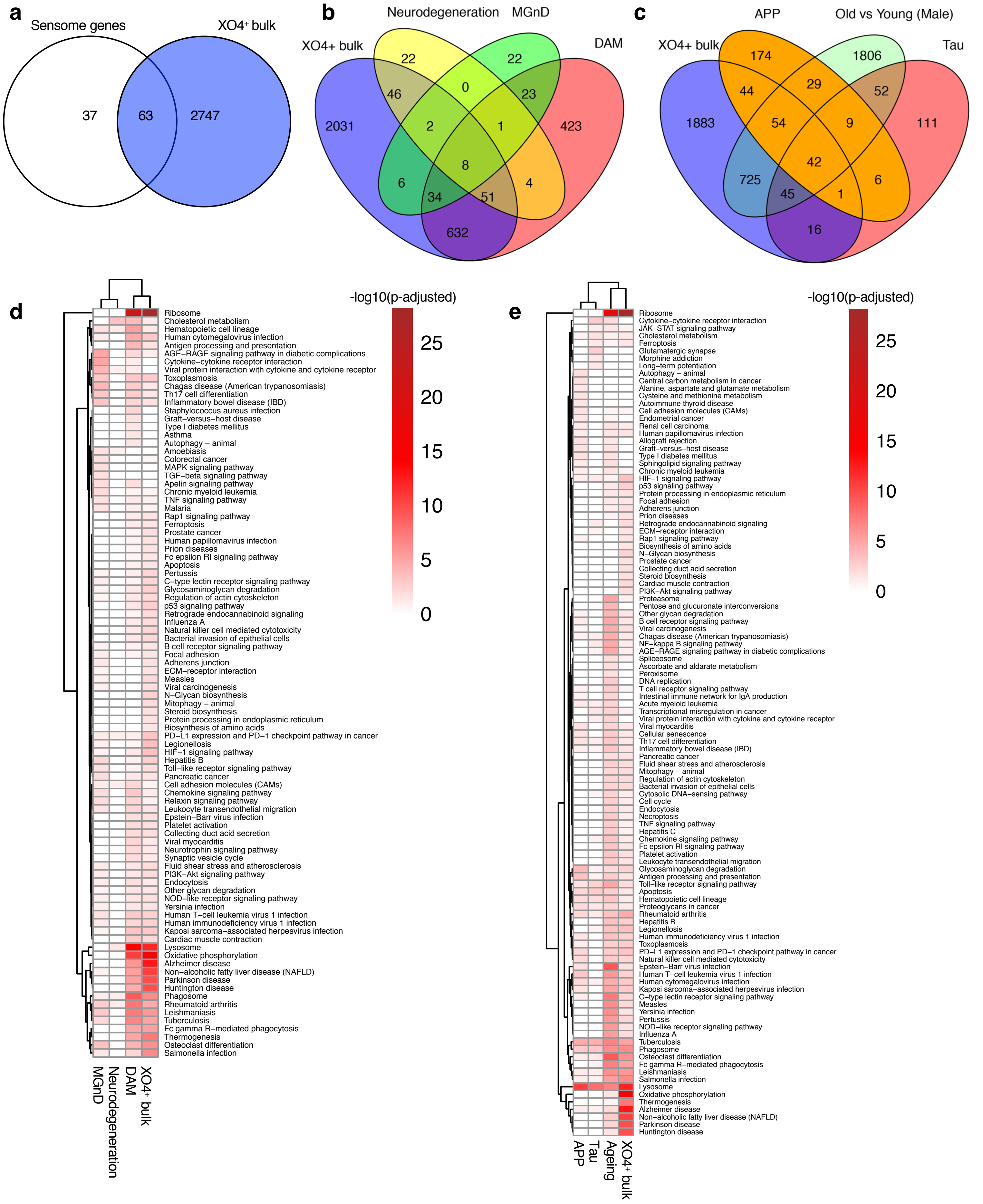

Supplementary Fig. 3a, Overlap between microglial sensome genes and $XO4^+$ genes (bulk RNA analysis), $p=6.1x10^{-11}$ by hypergeometric distribution test. **b-c,** Venn diagram showing overlap between microglial bulk gene expression signatures associated with $XO4^+$ microglia and other reported microglial signatures **b,** [1–3] or **c,** [4] **d-e,** Heatmap of enriched KEGG pathways in the microglial populations identified in each study **d,** [1–3] or **e,** [4] compared to bulk gene expression signatures associated with $XO4^+$ microglia, colored by $-\log_{10}$(adjusted p-value).

Supplementary Fig 4

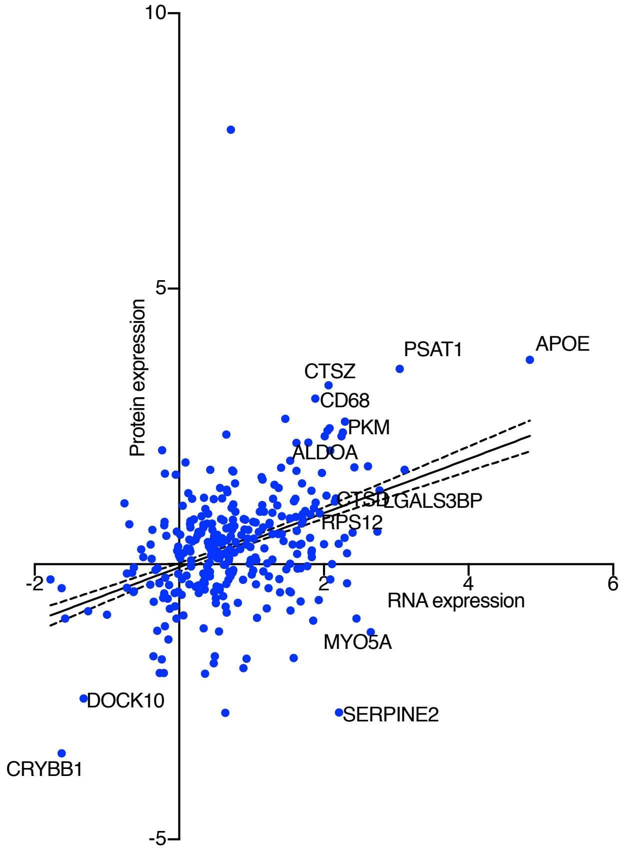

Supplementary Fig. 4 Scatter plot of DEPs and DEGs between XO4+ and WT groups. The linear regression and 95% confidence intervals are shown.

Supplementary Fig 5

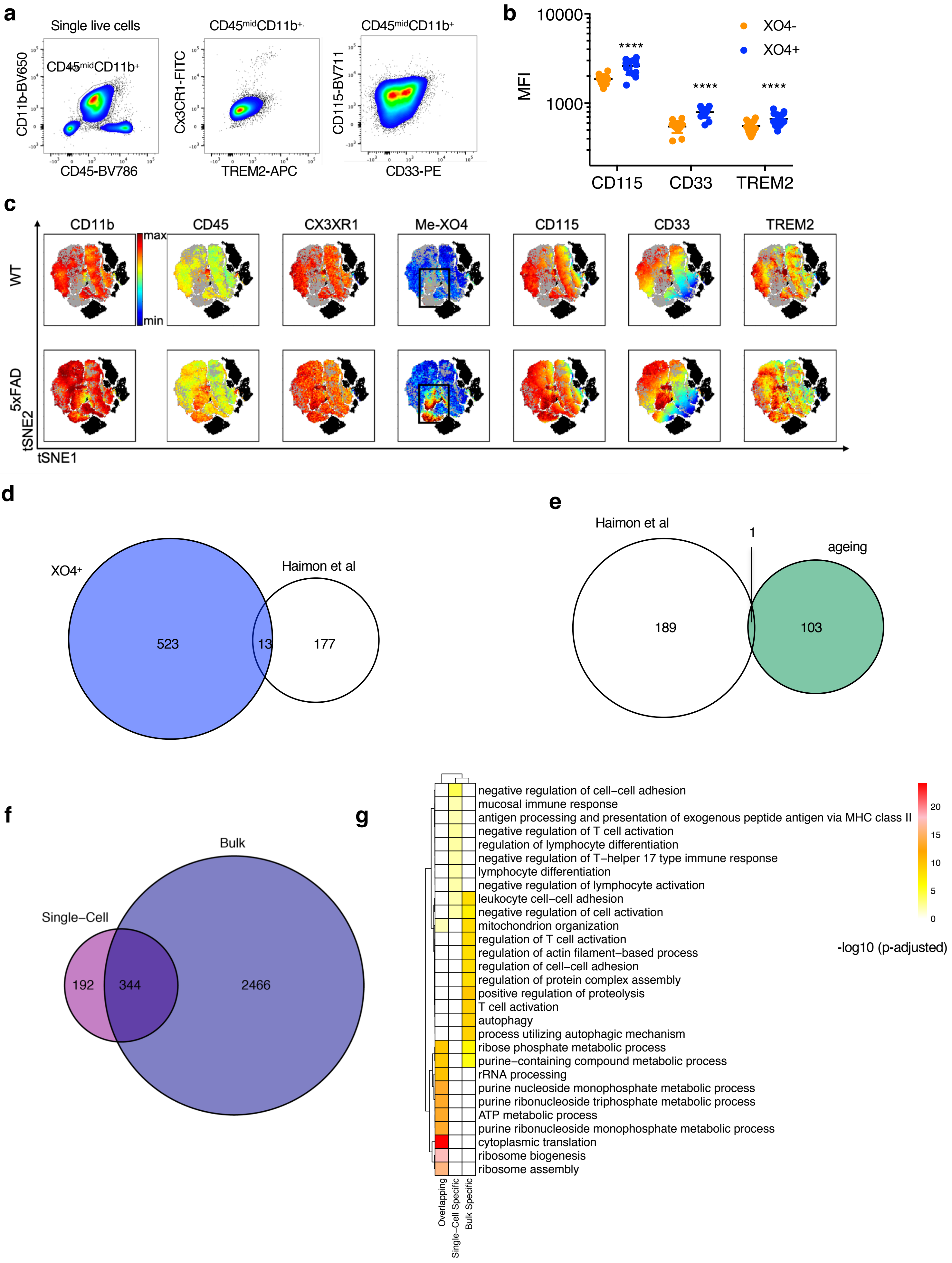

Supplementary Fig. 5a-c, To examine the heterogeneity inside the $XO4^+$ cell population, we employed dimensionality reduction representation (viSNE[5]) on our FACS datasets (**a**). We labelled myeloid cells with a subset of 6 microglial sensome markers[6] including LOAD risk factors CD33[7] and TREM2[8] that regulate microglial Aβ phagocytosis[9,10]. **b,** the median fluorescence intensity (MFI) of CD115, CD33 and TREM2 in 6m 5xFAD FACS-gated $XO4^-$ compared to $XO4^+$ microglia, pooled males and females, n=14 5xFAD mice. $XO4^+$ labeled microglia revealed uniformly high expression of CD33, TREM2 and the Csf1 receptor, CD115 (****, CD115 $p=6.5x10^{-6}$, CD33 $p=8.9x10^{-11}$, TREM2 $p=3.0x10^{-6}$ compared to $XO4^-$ microglia by 2-tailed paired t-test, df=13, t=7.249, 18.69, 7.792, respectively). **c,** viSNE, representative of n=3 mice per genotype, of myeloid cells isolated from WT (top) and 5xFAD (bottom) 6m male mice. Microglia ($CD11b^+CD45^{lo}CX3CR1^+$) are colored for expression of CD11b, CD45, CX3CR1, Methoxy-XO4, CD115, CD33 and TREM2, whereas remaining myeloid cells are black for clarity. The grey cells are microglia from the other genotype. All microglia homogeneously expressed CD11b, CX3CR1 and low levels of CD45, whereas expression of microglia-specific proteins, CD115, CD33 and TREM2 was highly variable in individual WT and 5xFAD $XO4^-$ microglia. **d-e,** Venn diagram showing overlap between **d,** the microglial gene expression signature associated with $XO4^+$ or **e,** ageing microglia from our study and the DEGs induced by sorting artifacts, previously reported in [4,11]. p-values of the overlap are 0.6556427 and 0.9379955, respectively, by hypergeometric test. **f,** Venn diagram showing overlap between single cell and bulk gene signatures associated with $XO4^+$ microglia and **g,** GO enrichment results of the overlapping (344 genes) and bulk or single-cell specific signature genes associated with $XO4^+$ microglia.

Supplementary Fig 6

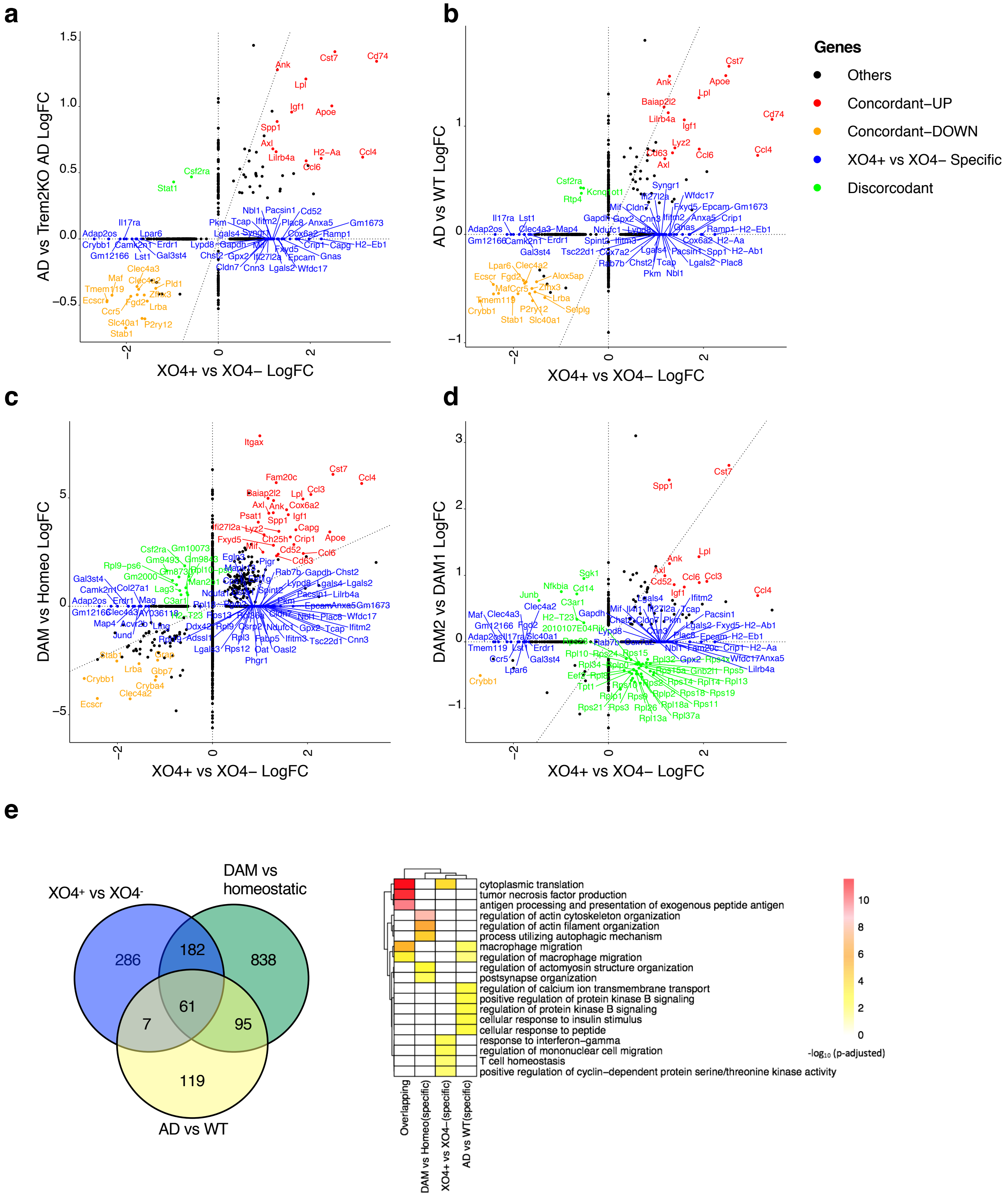

Supplementary Fig. 6a-d, Pairwise comparisons of DEGs between [12] and this study **(a-b)**, and [1] and this study **(c-d)**. Scatter plot of DEGs (false discovery rate, FDR<0.05) with $XO4^+$ and $XO4^-$ specific DEGs from single cell analyses with highest LogFC highlighted. Genes with concordant upregulation between each 2 datasets examined are highlighted in red, concordant downregulation are highlighted in orange. DEGs with discordant regulation are highlighted in green, and genes that are DE in XO4+ vs XO4- but not in the comparison dataset are highlighted in blue. **e,** Venn diagram showing the overlapping and distinct DEGs across the 3 datasets and GO enrichment results of the overlapping (61 genes) and dataset-specific gene sets i.e., $XO4^+$ vs $XO4^-$ (286 genes), DAM vs homeostatic (838 genes), and AD vs WT (119 genes).

Supplementary Fig 7

a Ageing pseudotime

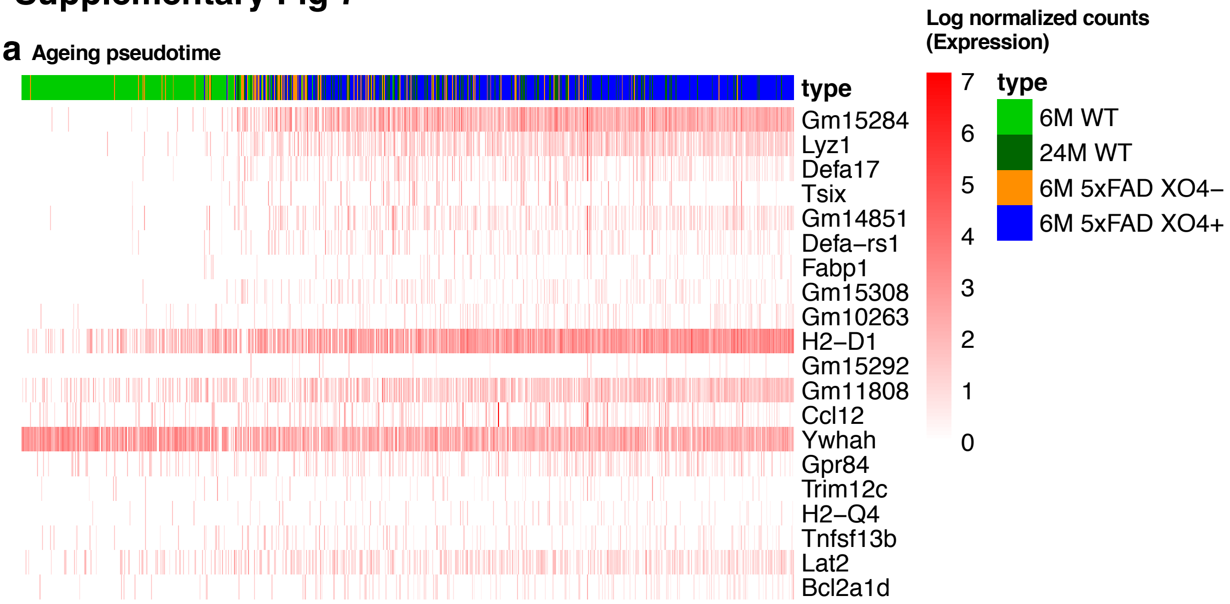

b Phagocytosis pseudotime

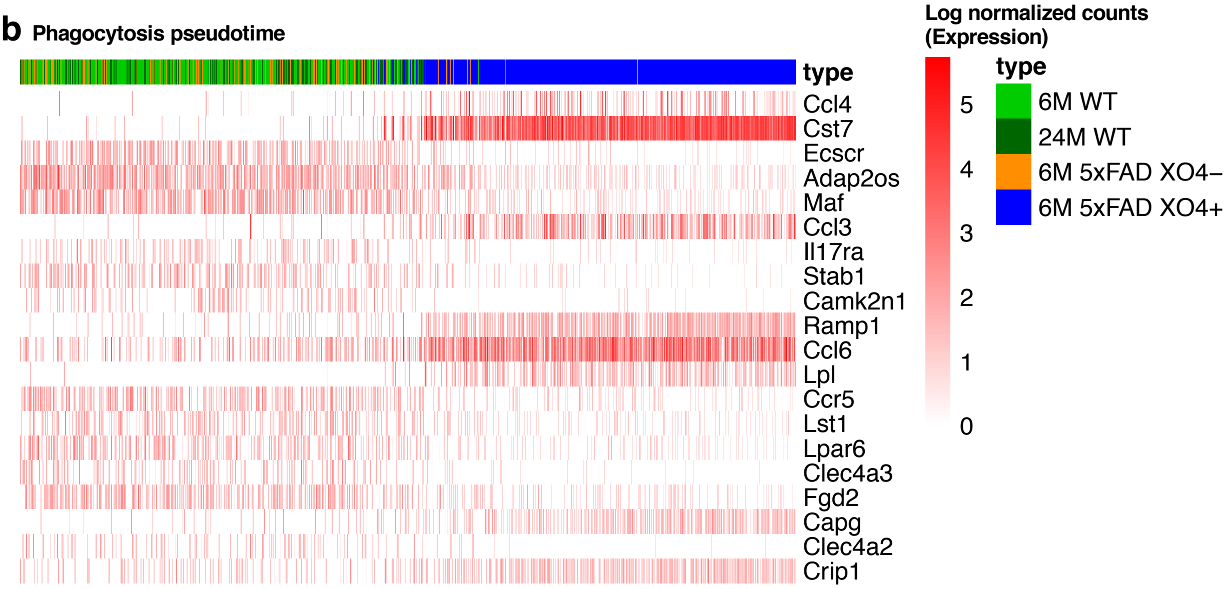

Supplementary Fig. 7 a-b, Log_2CPM Expression Heatmap of top 20 trajectory-specific DE based on absolute log Fold Change (24M WT vs 6M WT and $XO4^+$ vs $XO4^-$) ordered by **a**, age pseudotime or **b,** phagocytosis pseudotime, respectively.

Supplementary Fig 8

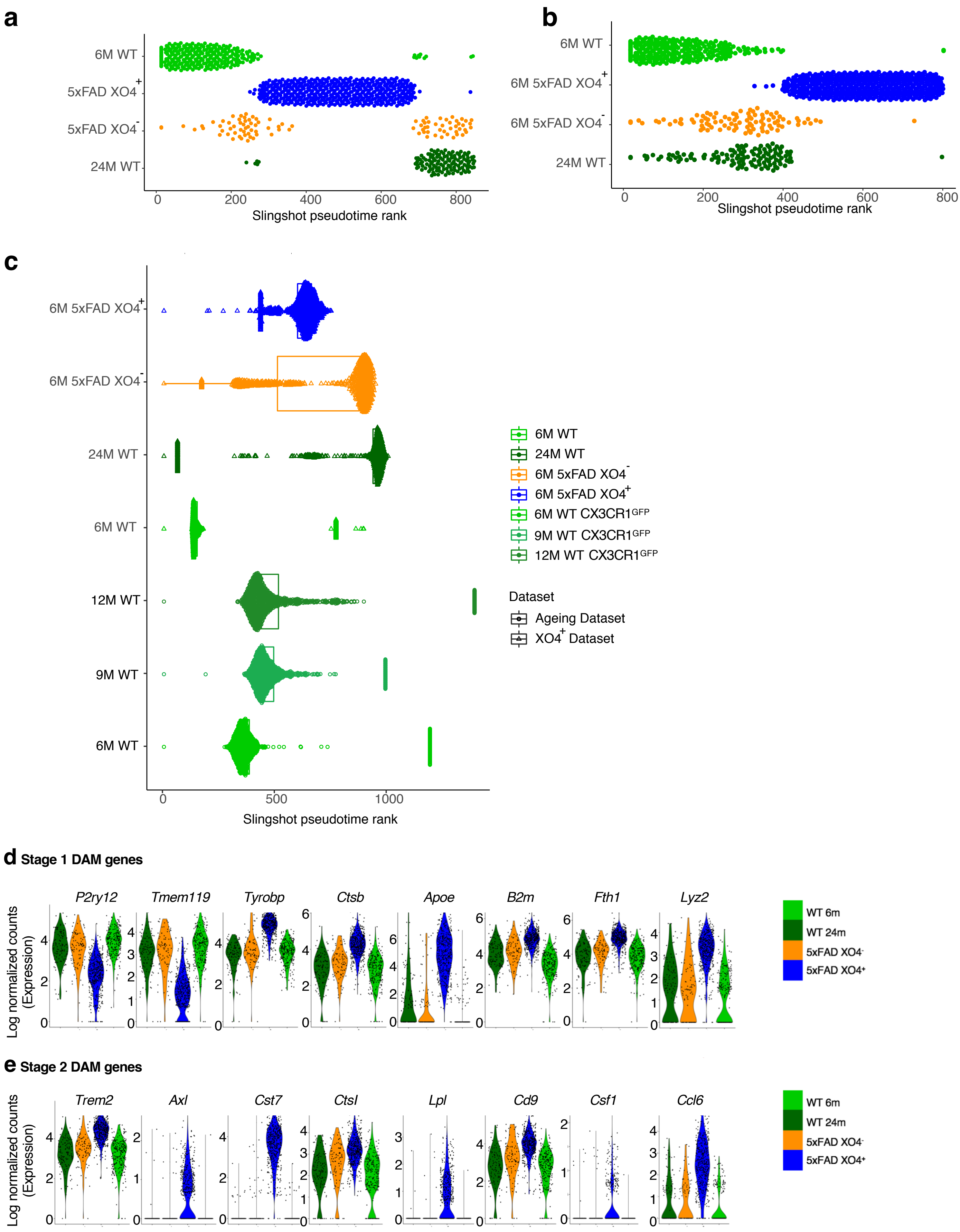

Supplementary Fig. 8 a-b, Slingshot pseudotime trajectory analysis of 6M WT, 24M WT, 6M 5xFAD XO4$^-$ and 6M 5xFAD XO4$^+$ microglia. **c** Slingshot pseudotime trajectory analysis of 6M, 9M, 12M CX3CR1^{GFP} and 6M WT, 24M WT, 6M 5xFAD XO4$^-$ and 6M 5xFAD XO4$^+$ microglia. The lower, middle, and upper hinges represent the lower quartile, median, and upper quartile respectively while the upper and lower whiskers extend ± 1.5 times of the interquartile range from the corresponding hinges. **d-e,** Violin plots showing Log_2CPM expression of **d,** Stage I DAM and **e,** Stage II DAM genes[1] in a priori cell clusters.

Supplementary Fig 9

a

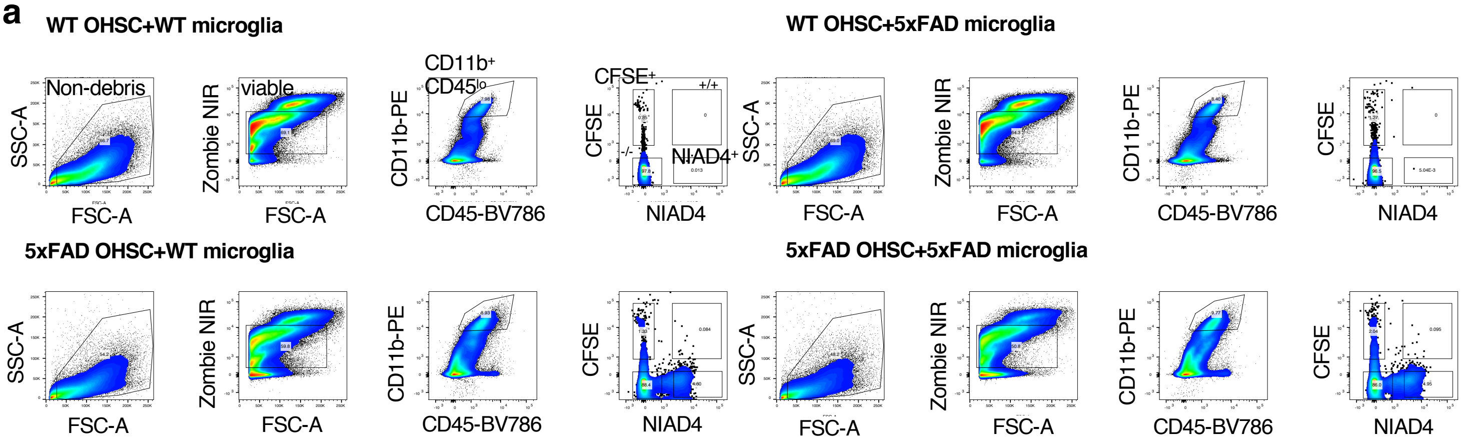

b Genes downregulated in NIAD4+

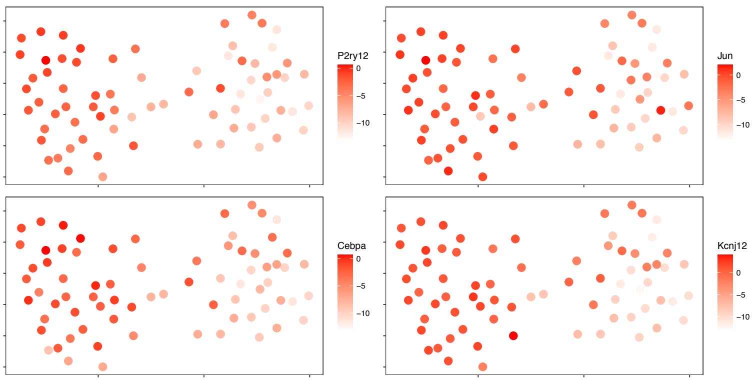

c Genes upregulated in NIAD4+

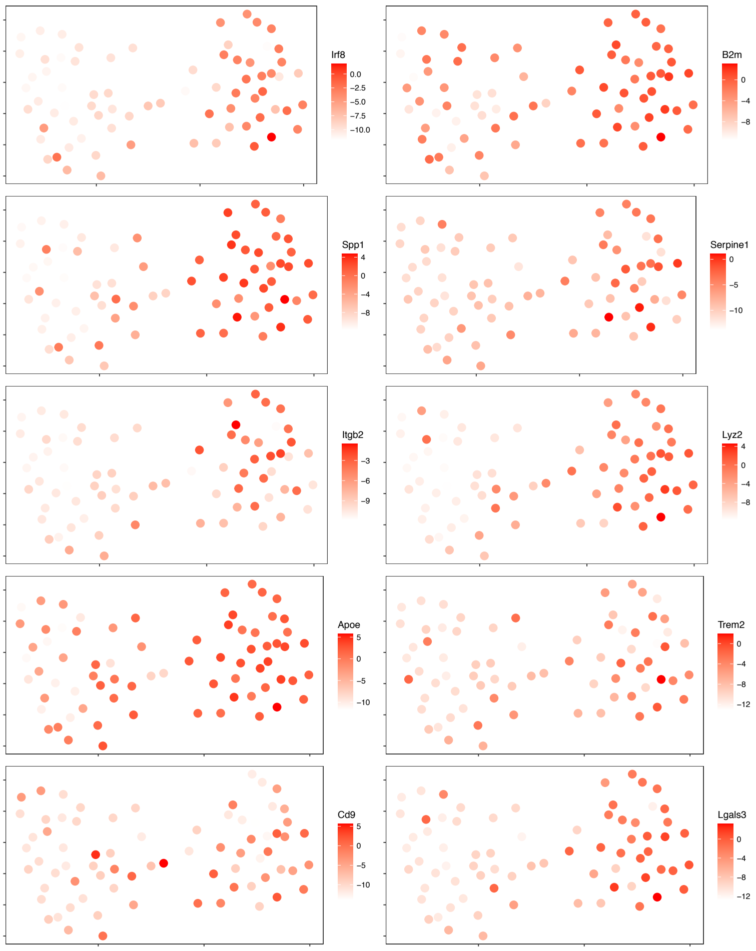

Supplementary Fig. 9a, FACS gating/sorting strategy for data presented in Figure 3. Heatmap of gene expression in microglia isolated from OHSCs visualized with a k-nearest-neighbor graph rendered using a force directed layout[13], colored by log2 transformed ΔCt values of selected genes upregulated in **b,** homeostatic microglia or **c,** NIAD4^{+} (XO4^{+}) microglia.

Supplementary Fig 10

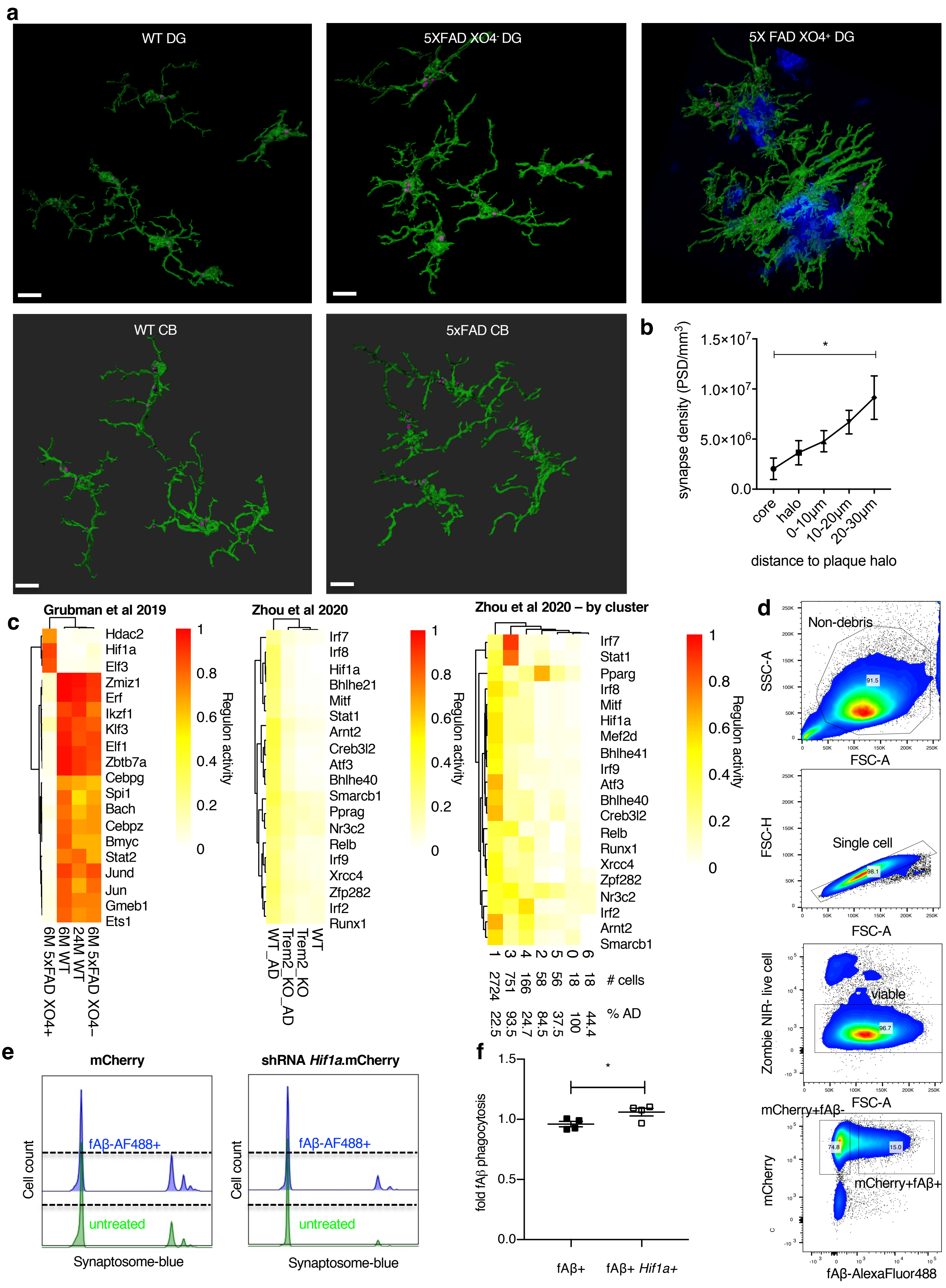

Supplementary Fig. 10a, Representative 3D reconstruction of confocal z-stacks showing PSD95 internalized within 6m WT, 5xFAD XO4$^-$ or 5xFAD XO4$^+$ microglia cells (scale bars = 15 µm). **b**, the relationship between synapse density and distance from plaques as calculated by IMARIS from hippocampal z-stack images from n = 5 5xFAD animals. Data are expressed as mean ± SEM of volume of PSD95 staining per mm^3 within regions at each distance from the plaque. p=0.017 by 1-way ANOVA and Dunnet's multiple comparison test. **c,** pySCENIC regulon analysis showing that *Hif1a*, *Elf3* and *Hdac2* are predicted to control the XO4$^+$ gene regulatory network, and the AD and Cluster 1 network from [12] are predicted to be controlled by *Irf8, Arnt2, Smarcb1, Mitf, Hif1a, Bhlhe41, Creb3l2, Atf3, Bhlhe40* and *Mef2d*. Clustering was performed on [12] (Synapse DOI: 10.7303/syn21125841) with a resolution of 0.4 after filtering for microglia and preprocessing. For each cell, pySCENIC outputs a binary score for each regulon with 1 indicating on state and 0 indicating off state. The regulon activity score plotted here is the percentage of cells (within each group) with the regulon turned on. The top 20 regulons in terms of variance across a priori cell group labels are visualised. **d,** FACS gating strategy for sorting shRNA *Hif1a.*mCherry and mCherry BV2 cells that were AF488-fAβ$^+$ or AF488-fAβ$^-$, presented in Fig. 4f. **e,** Representative FACS histograms showing fluorescence intensity for synaptosomes in *mCherry* and *shRNA Hif1a mCherry* BV2 cells. AF488-fAβ$^+$ cells are gated on AF488 fluorescence. **f,** The proportion of cells that are phagocytic for fluorescent fAβ following transduction (or not) with a dox-inducible *Hif1a* overexpression construct and treatment with fAβ. Data are expressed as fold change in % phagocytosis relative to fAβ-treated dox -ve controls (mean ± SEM, 4 independent experiments, *p=0.038 by 2 tailed paired t-test, t=3.536, df=3).

Supplementary Fig 11

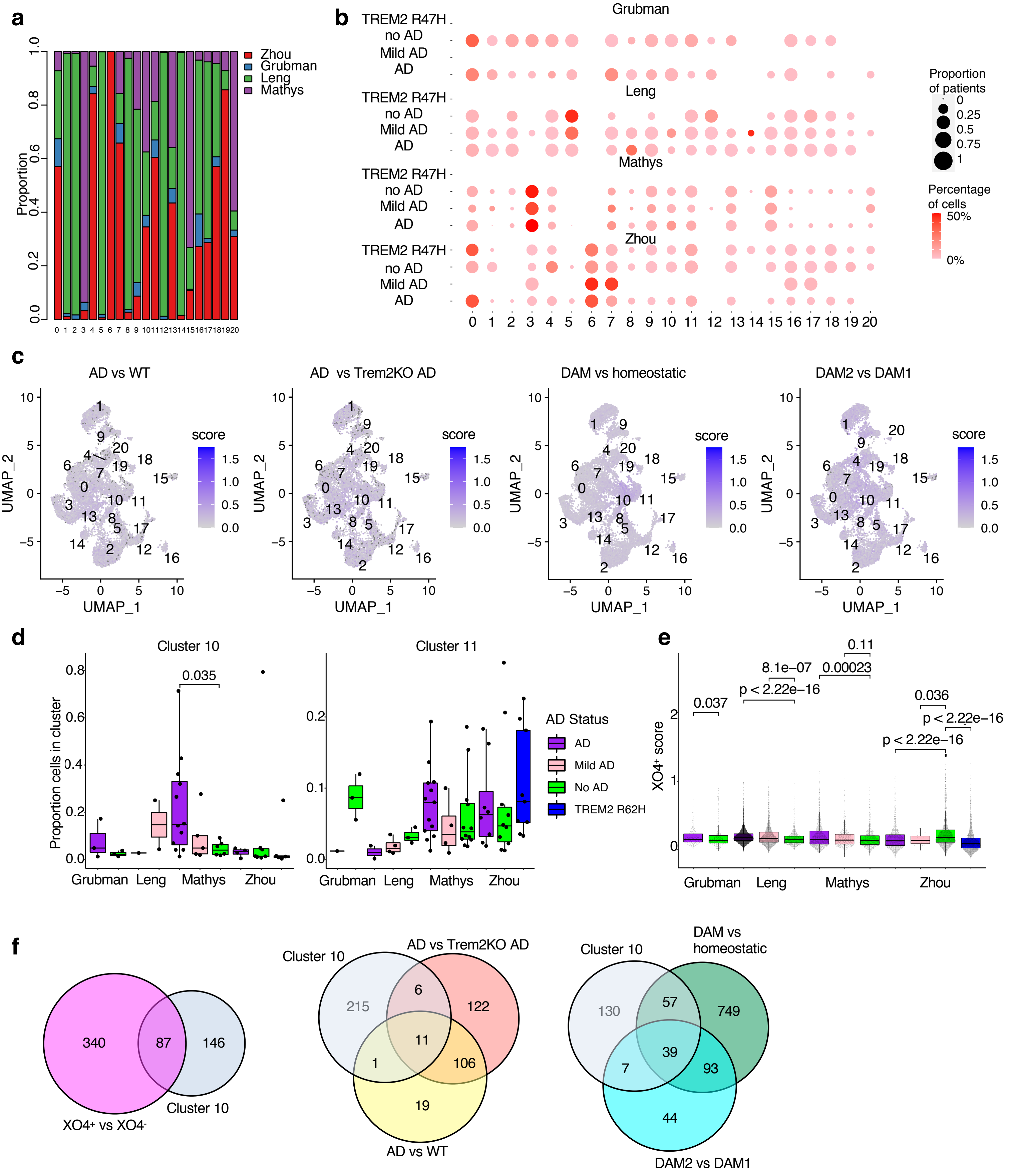

Supplementary Fig. 11a, The proportion of cells in each cluster from each study, and **b,** bubble plot showing the proportion of cells in each cluster that are from AD, mild AD, no AD patients in each study. The size of the circle represents the proportion of patients in the group with any cells in that cluster, and the color of the circle represents the median percentage of cells in the cluster for patients with any cells in that cluster. **c,** UMAP projection of single microglia nuclei from control and AD patient entorhinal and frontal cortex samples, combined by integrating data from [12,14–16], comprising 102 patients. Clustering and analysis of gene expression signature scores is performed using Seurat v3. UMAP projection is colored by each gene expression signature score. **d,** box plots showing the proportion of patient microglia within Cluster 10 (left) and Cluster 11 (right) for patients with microglia in Cluster 10 (Grubman: n = 12 patients, Leng: n = 3 patients, Mathys n = 23 patients, Zhou: n = 16 patients) or 11 (Grubman: n = 8 patients, Leng: n = 9 patients, Mathys n = 29 patients, Zhou: n = 27 patients), respectively, separated by study and diagnosis ($p=0.035$, Wilcoxon test with No AD as reference). The lower, middle, and upper hinges represent the lower quartile, median, and upper quartile respectively while the upper and lower whiskers extend ± 1.5 times of the interquartile range from the corresponding hinges. **e,** box plots showing the distribution of each gene expression signature score, split by study, disease diagnosis and TREM2 genotype (Wilcoxon test with No AD as reference; n = 101 patients, 1 patient classified as other dementia excluded). The lower, middle, and upper hinges represent the lower quartile, median, and upper quartile respectively while the upper and lower whiskers extend ± 1.5 times of the interquartile range from the corresponding hinges. **f,** Overlap between Cluster 10 DEGs and mouse microglia gene expression signatures. biomaRt was used for converting mouse gene symbols to human gene symbols.

Supplementary Fig 12

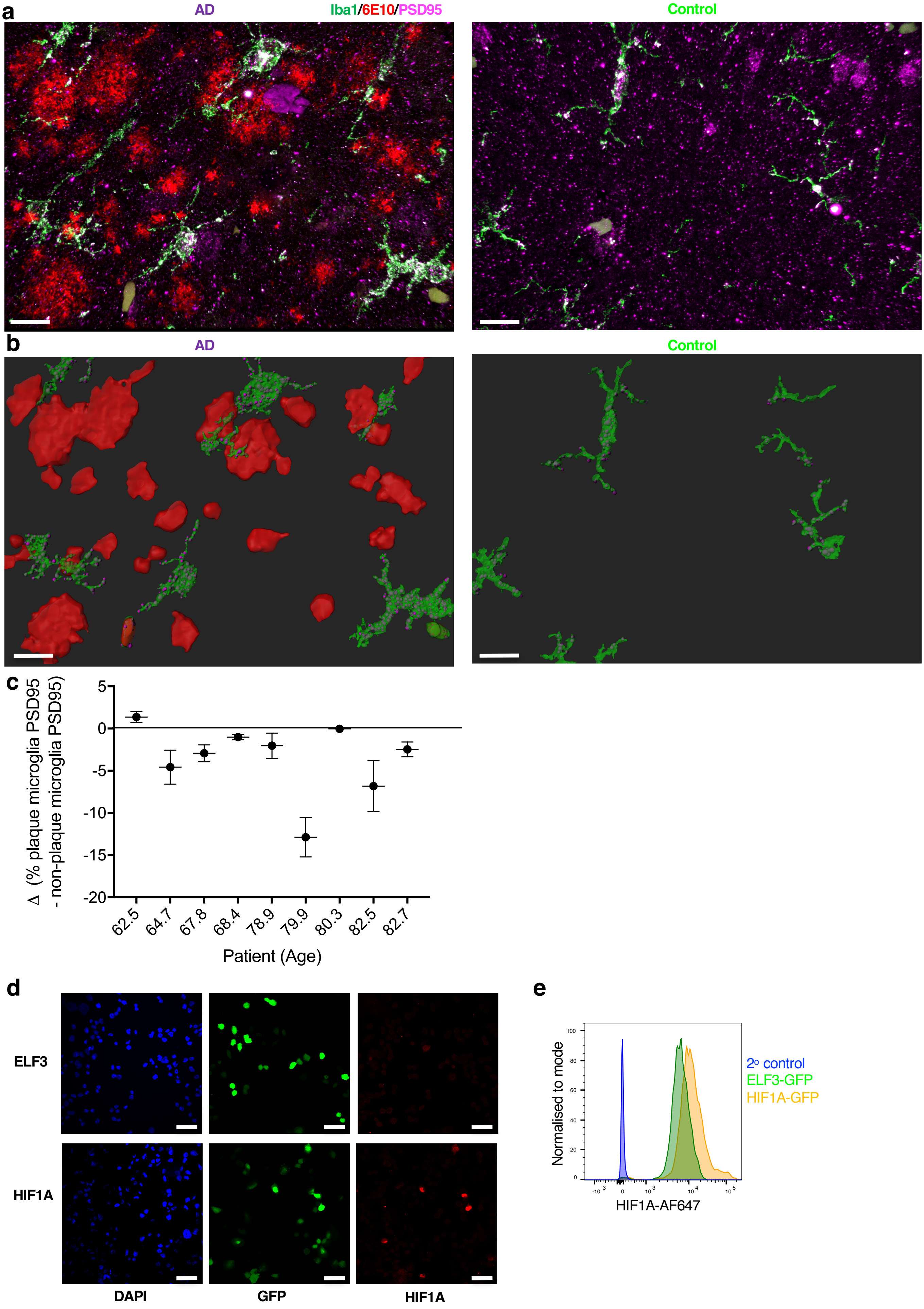

Supplementary Fig. 12a, Representative z-stack 3D projection and **b,** Imaris 3D reconstruction showing PSD95 internalized within microglia cells in human frontal cortex sections from AD patients (n = 9) and cognitively normal individuals (n = 8) stained with PSD95, 6E10 and Iba1 (scale bars for a and b = 15 μm)**. c,** quantitation of PSD95 internalized within microglia that are plaque adjacent or plaque distal, n = 9 AD patients, with n = 21, 22, 16, 18, 4, 3, 11, and 11 $XO4^+$ microglia, and 28, 13, 16, 9, 15, 8, 15, 10 and 11 $XO4^-$ microglia analyzed per patient, respectively. Data are mean ± SEM for individual microglia in each patient. p=0.02 using paired 1-tailed one-sample t-test to test whether the mean differences in PSD95 within plaque adjacent compared to plaque-distal microglia are significantly different from 0. **d-e,** Expression of HIF1A by **d,** immunofluorescence, scale bars = 50 μm and **e,** intracellular FACS staining in stable *ELF3* or *HIFA*-overexpressing cells.

Supplementary Fig 13

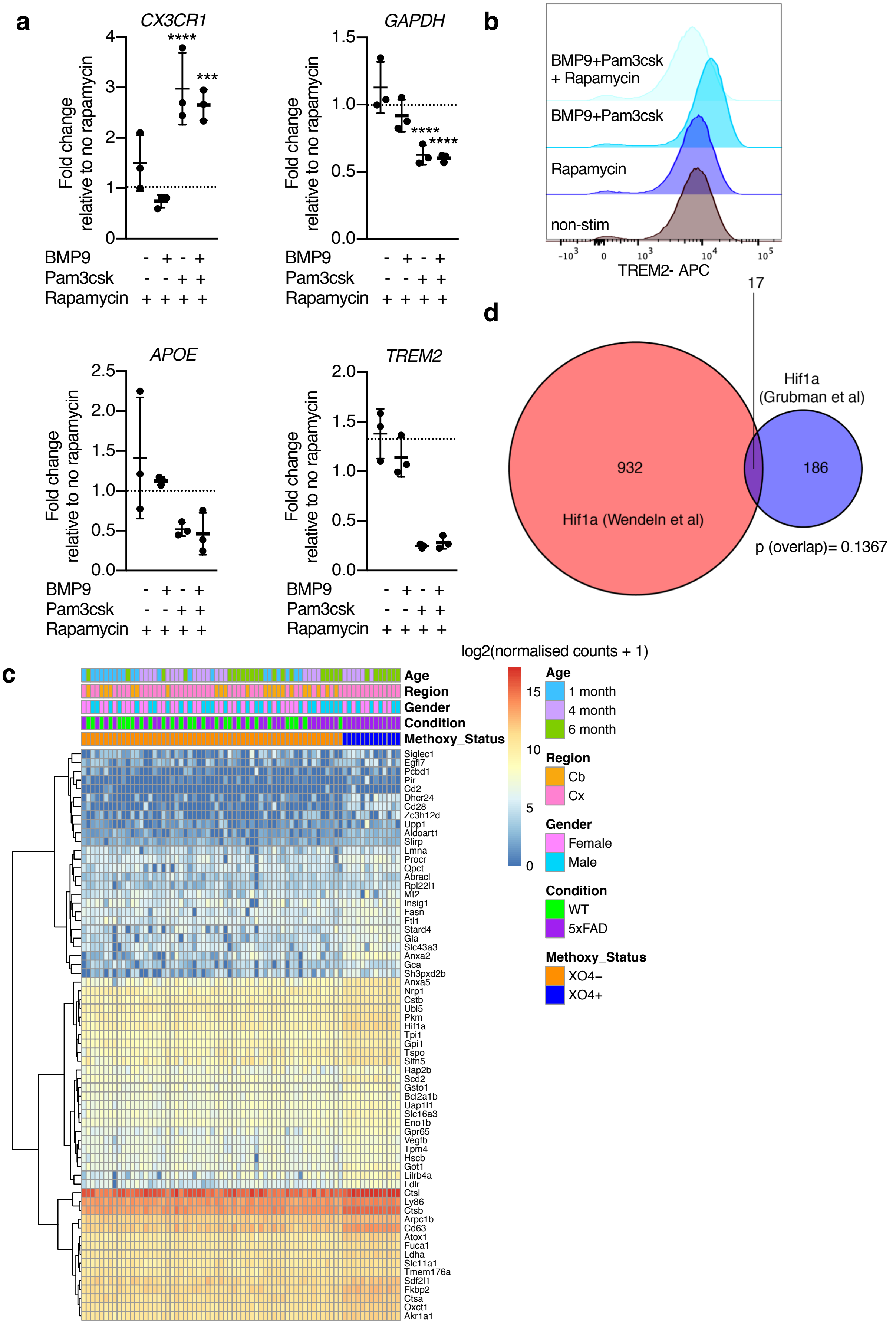

Supplementary Fig. 13a, Stimulation of iMGLs with MyD88-dependent TLR agonist Pam3csk (alone or with BMP9) induces *GAPDH* as predicted, but also represses *APOE* and *TREM2*, genes associated with $XO4^+$ microglia but not in the *Hif1a* regulon. Data are fold changes induced by rapamycin normalized to each respective treatment in the absence of rapamycin, n=3 independent experiments. *CX3CR1* ****$p=8.0x10^{-6}$, ***p=0.00017; *GAPDH* ****$p=2.0x10^{-5}$, $p=1.6x10^{-5}$, respectively by two-way ANOVA and Holm-Sidak post-test (comparing each treatment with rapamycin to the equivalent treatment in absence of rapamycin), n=3 independent experiments. **b**, TREM2 surface protein expression in iMGLs following treatment with BMP9 and Pam3csk is reduced by the mTOR inhibitor, rapamycin, as measured by FACS. **c,** heatmap showing concordant overlap of DEGs associated with the phagocytic signature in $XO4^+$ microglia from bulk RNA-seq data compared to human DEGs induced by Pam3csk and repressed by rapamycin in iMGLs. **d,** Overlap between the *Hif1a* module from Wendeln *et al.*[64] and the *Hif1a* regulon described in this study.

Supplementary Fig 14

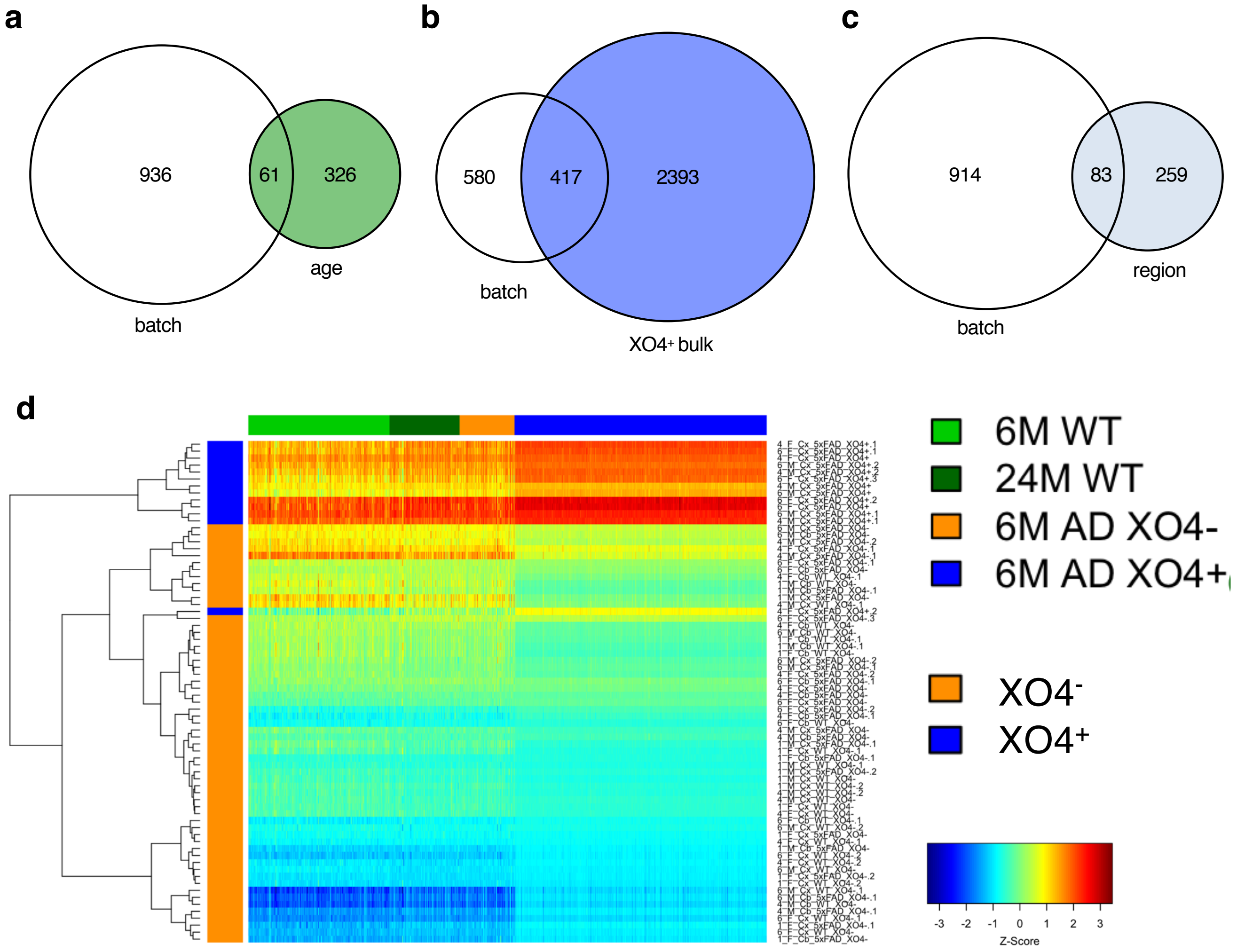

Supplementary Fig. 14a-c, Overlap between batch-associated and age-associated genes (DESeq2 Likelihood Ratio Test FDR<0.01), **a,** 15.8% (61 out of 387), **b,** XO4-associated genes 14.8% (417 out of 2810) and **c,** region associated genes 24.3% (83 out of 342). **d,** Projection of single cell RNA-seq data onto bulk RNA-seq data using Reference Component Analysis (RCA).

Supplementary References

1. Keren-Shaul, H. *et al.* A Unique Microglia Type Associated with Restricting Development of Alzheimer's Disease. *Cell* **169**, 1276–1290.e17 (2017).
2. Krasemann, S. *et al.* The TREM2-APOE Pathway Drives the Transcriptional Phenotype of Dysfunctional Microglia in Neurodegenerative Diseases. *Immunity* **47**, 566–581.e9 (2017).
3. Friedman, B. A. *et al.* Diverse Brain Myeloid Expression Profiles Reveal Distinct Microglial Activation States and Aspects of Alzheimer's Disease Not Evident in Mouse Models. *Cell Rep.* **22**, 832–847 (2018).
4. Kang, S. S. *et al.* Microglial translational profiling reveals a convergent APOE pathway from aging, amyloid, and tau. *J. Exp. Med.* **215**, 2235–2245 (2018).
5. Amir, E.-A. D. *et al.* viSNE enables visualization of high dimensional single-cell data and reveals phenotypic heterogeneity of leukemia. *Nat. Biotechnol.* **31**, 545–552 (2013).
6. Hickman, S. E. *et al.* The microglial sensome revealed by direct RNA sequencing. *Nat. Neurosci.* **16**, 1896–1905 (2013).
7. Bertram, L. *et al.* Genome-wide association analysis reveals putative Alzheimer's disease susceptibility loci in addition to APOE. *Am. J. Hum. Genet.* **83**, 623–632 (2008).
8. Guerreiro, R. *et al.* TREM2 variants in Alzheimer's disease. *N. Engl. J. Med.* **368**, 117–127 (2013).
9. Griciuc, A. *et al.* Alzheimer's disease risk gene CD33 inhibits microglial uptake of amyloid beta. *Neuron* **78**, 631–643 (2013).
10. Yuan, P. *et al.* TREM2 Haplodeficiency in Mice and Humans Impairs the Microglia Barrier Function Leading to Decreased Amyloid Compaction and Severe Axonal Dystrophy. *Neuron* **92**, 252–264 (2016).
11. Haimon, Z. *et al.* Re-evaluating microglia expression profiles using RiboTag and cell isolation strategies. *Nat. Immunol.* **19**, 636–644 (2018).
12. Zhou, Y. *et al.* Human and mouse single-nucleus transcriptomics reveal TREM2-dependent and TREM2-independent cellular responses in Alzheimer's disease. *Nat. Med.* **26**, 131–142 (2020).
13. Weinreb, C., Wolock, S. & Klein, A. M. SPRING: a kinetic interface for visualizing high

dimensional single-cell expression data. *Bioinformatics* **34**, 1246–1248 (2018).

14. Spina, S., Heinsen, H., Grinberg, L. T. & Kampmann, M. T. Molecular characterization of selectively vulnerable neurons in Alzheimer's Disease. *bioRxiv* (2020).
15. Mathys, H. *et al.* Single-cell transcriptomic analysis of Alzheimer's disease. *Nature* (2019) doi:10.1038/s41586-019-1195-2.
16. Grubman, A. *et al.* A single-cell atlas of entorhinal cortex from individuals with Alzheimer's disease reveals cell-type-specific gene expression regulation. *Nat. Neurosci.* **22**, 2087–2097 (2019).
17. Wendeln, A.-C. *et al.* Innate immune memory in the brain shapes neurological disease hallmarks. *Nature* **556**, 332–338 (2018).
18. Krämer, A., Green, J., Pollard, J., Jr & Tugendreich, S. Causal analysis approaches in Ingenuity Pathway Analysis. *Bioinformatics* **30**, 523–530 (2014).
