# Supplementary material for: Transcriptional signature in microglia associated with Aβ plaque phagocytosis
Source: Nat Commun. 2021 May 21;12:3015. doi: 10.1038/s41467-021-23111-1 (PMC8140091; doi:10.1038/s41467-021-23111-1)
Supplement: Supplementary file 2 — Description of Additional Supplementary Files [file 41467_2021_23111_MOESM2_ESM.pdf]

Description of Additional Supplementary Files

Supplementary Data 1 Covariate associated genes and corresponding functional enrichment results. (Tab 1) List of covariate genes associated with or overlapping between age (n = 387 genes), region (n = 342 genes) and $XO4^+$-associated gene expression signatures (n = 2,810 genes) from bulk RNA-sequencing analysis (see Methods for details). When more than one covariate is associated with a gene, this is indicated as follows, CovariateA_CovariateB_CovariateX. (Tabs 2-8) Gene Ontology (GO) and Kyoto Encyclopedia of Genes and Genomes (KEGG) pathways enrichment results for the genes associated with each covariate. Only significant enrichments are reported (adjusted p-value <0.05). P-values were adjusted using the default g:SCS algorithm from the gProfiler R package. The genes contributing the significant functional enrichment are also reported.

Supplementary Data 2 Functional enrichment results for $XO4^+$ microglia genes.
Gene Ontology (GO) and Kyoto Encyclopedia of Genes and Genomes (KEGG) pathways/terms enriched in gene expression signatures identified only in $XO4^+$ microglia in bulk (n = 2,031 genes) and single cell (n = 284 genes) analyses, respectively. Only significant pathways/terms are reported (adjusted p-value < 5%). p.adjust refers to Benjamini-Hochberg corrected p-value for the significance of enrichment. Genes contributing to the enrichment are also reported. Gene lists for microglial gene expression signatures (Fryer et al., DAM, MGnD, Friedman Neurodegeneration)[13,14,31,33] used in these comparisons are also provided.

Supplementary Data 3 Relationship between $XO4^+$ associated DEPs and DEGs.

Supplementary Data 4 Details of peptides in the mass spectrometry analysis.
The peptides used for normalization of LC-SWATH-MS data, the peptides detected in spectral library samples from microglia and purified synaptosomes, and raw peptides concentrations detected in this study.

Supplementary Data 5 DEGs from single cell RNA-seq analysis.
List of DEGs in single cell analysis of FACS-sorted microglia. (i) Ageing DEGs (n = 104 genes with FDR<0.05), (ii) $XO4^+$ DEGs (n = 536 genes with FDR<0.05), (iii) specific ageing DEGs (i.e., non-overlapping with $XO4^+$ DEGs, n = 42 genes with FDR<0.05), (iv) specific $XO4^+$ DEGs (i.e., non-overlapping with Ageing DEGs, n = 474 genes with FDR<0.05), (v) list of DEGs overlapping between (i) and (ii) (n = 62 genes with FDR<0.05). LFC, Log_2(Fold Change); Log CPM, Log_2(Counts Per Million); F, F statistic (edgeR output); FDR, false discovery rate was estimated using the Benjamini-Hochberg method.

Supplementary Data 6 qPCR data.
Raw low input qPCR data from OHSCs, Ct values.

Supplementary Data 7 Single Cell Regulatory Network Inference and Clustering (SCENIC) and pySCENIC results.
(Tab 1 - Regulon activity) For each of the predicted top 10 regulons (*Hif1a*, *Elf3, Spi1, Erf, Fli1, Elf2, Maf, Etv3, Ets1,* and *Irf2*), we report the mean of normalized regulatory activity scores in each a priori cell type group (i.e., 6M WT, 24M WT, 6M 5xFAD $XO4^-$ and 5xFAD $XO4^+$). Each regulon includes at least 100 genes that are expressed in at least 10% of the cells. (Tabs 2-11) Genes that

belong to each regulon. (Tab 12) Raw binarized pySCENIC results for Grubman et al. (893 microglia). (Tab 13) Raw binarized pySCENIC results for Zhou et al. (3,791 microglia).

Supplementary Data 8 Analysis of human microglia clusters.
Tab 1 - Cluster composition of microglia in each patient
Tab 2 - DEGs associated with each human microglial cluster.

Supplementary Data 9 Ingenuity Pathway Analysis (IPA) results.
The upstream activators of the *Hif1a* regulon predicted by IPA[54]. The activation z-score is a statistical measure of the match between the expected relationship direction of regulation and the observed gene expression. Positive z-scores are indicative of predicted activation. p-value of overlap refers to the significance of the overlap between the *Hif1a* regulon gene set and the regulated target genes predicted by IPA.

Supplementary Data 10 Functional enrichment results for the $XO4^+$ genes modulated by upstream activators.
Kyoto Encyclopedia of Genes and Genomes (KEGG) pathways enriched in the gene set comprising the overlap (n = 65 genes) between (i) the network of genes induced by Pam3csk and repressed by rapamycin (n = 866 genes) and (ii) the gene expression signature associated with $XO4^+$ microglia (n = 1,092 genes). Only significant KEGG pathways are reported (adjusted p-value <5%). p.adjusted refers to Benjamini-Hochberg corrected p-value for the significance of enrichment. Genes contributing to the enrichment are also reported.

Supplementary Data 11 8bp sample index sequences used for sample multiplexing for bulk RNA-seq.

Supplementary Data 12 List of TaqMan gene expression assays used for qPCR by Biomark Fluidigm 96.96.
