# Supplementary material for: Transcriptional signature in microglia associated with Aβ plaque phagocytosis
Source: Nat Commun. 2021 May 21;12:3015. doi: 10.1038/s41467-021-23111-1 (PMC8140091; doi:10.1038/s41467-021-23111-1)
Supplement: Supplementary file 15 — Reporting Summary [file 41467_2021_23111_MOESM15_ESM.pdf]

nature research

Corresponding author(s): Jose M. Polo, Enrico Petretto and Alexandra Grubman

Last updated by author(s): 2021/02/09

Reporting Summary

Nature Research wishes to improve the reproducibility of the work that we publish. This form provides structure for consistency and transparency in reporting. For further information on Nature Research policies, seeAuthors & Referees and theEditorial Policy Checklist .

Statistics

For all statistical analyses, confirm that the following items are present in the figure legend, table legend, main text, or Methods section.

n/a	Confirmed	
☐	☒	The exact sample size (*n*) for each experimental group/condition, given as a discrete number and unit of measurement
☐	☒	A statement on whether measurements were taken from distinct samples or whether the same sample was measured repeatedly
☐	☒	The statistical test(s) used AND whether they are one- or two-sided<br>*Only common tests should be described solely by name; describe more complex techniques in the Methods section.*
☐	☒	A description of all covariates tested
☐	☒	A description of any assumptions or corrections, such as tests of normality and adjustment for multiple comparisons
☐	☒	A full description of the statistical parameters including central tendency (e.g. means) or other basic estimates (e.g. regression coefficient) AND variation (e.g. standard deviation) or associated estimates of uncertainty (e.g. confidence intervals)
☐	☒	For null hypothesis testing, the test statistic (e.g. *F*, *t*, *r*) with confidence intervals, effect sizes, degrees of freedom and *P* value noted<br>*Give P values as exact values whenever suitable.*
☒	☐	For Bayesian analysis, information on the choice of priors and Markov chain Monte Carlo settings
☒	☐	For hierarchical and complex designs, identification of the appropriate level for tests and full reporting of outcomes
☐	☒	Estimates of effect sizes (e.g. Cohen's *d*, Pearson's *r*), indicating how they were calculated

Our web collection on statistics for biologists contains articles on many of the points above.

Software and code

Policy information about availability of computer code

Data collection	BD FACSDiva Software (v8.0.1) for BD FACSAria™ III cell sorter and BD LSRFortessa™ X-20 Flow Cytometer, Leica Application Suite X (v3.5.7.23225) for Leica SP8 confocal microscope, LightCycler® 480 Software (v1.5.1.62) for LightCycler® 480 Real-Time PCR System
Data analysis	SCATER(version 1.6.1), SCRAN(version 1.6.6), SINGLE CELL EXPERIMENT (version 1.0.0), SEURAT(version 2.1.0, 2.3.4, 3.1.5), M3DROP (version 3.5.0), SC3 (Single cell consensus clustering)(version 1.7.6), MCLUST(version 5.4), EdgeR(3.20.8), SCENIC(version 0.1.7), GENIE3 (version 1.0.0), AUCell(version 0.99.5), RcisTarget(version 0.99.0) , Reference Component Analysis (RCA) (2.0), Ingenuity Pathway Analysis (IPA) (27th June 2018), pheatmap (version 1.0.8 and 1.0.12), DESeq2 (version 1.16.1 and version 1.18), gProfileR (version 0.6.4), 10x Genomics Cell Ranger (version 3.1.0), bioMart (2.42.1), ggplot2(version 3.0.0), Cytobank (version 6.1.2), ggbeeswarm (0.6.0), igraph (1.2.2), magrittr (1.5), dplyr (0.7.8), RColorBrewer (1.1.2), gplots (3.0.1), preprocessCore (1.40.0), flashClust (1.1.2), R.utils (2.6.0), destiny (2.6.1), pyscenic (0.10.3), ggsignif (0.6.0) , ggpubr (0.4.0), Slingshot(v.1.4.0), scds(1.2.0), clusterprofiler (3.14.3), gridExtra(2.3),ggthemes (4.2.0), ggrepel(0.8.2), rstatix(0.6.0), VennDiagram(1.6.20), org.Mm.eg.db (3.10.0), Matrix(1.2.18), readxl(1.3.1), IMARIS (v8.3.1), FlowJo (v9), SPRING (https://kleintools.hms.harvard.edu/tools/spring.html), GraphPad Prism (v7.0e), limma (3.34.9), biobase (2.46.0), matrixStats (0.57.0), BRETIGEA (1.0.0), scales(1.1.1), Fluidigm Real-Time PCR Analysis software(v4.1.2), annotables (0.1.91), DOSE (3.12.0), reshape2 (1.4.4)

For manuscripts utilizing custom algorithms or software that are central to the research but not yet described in published literature, software must be made available to editors/reviewers. We strongly encourage code deposition in a community repository (e.g. GitHub). See the Nature Research guidelines for submitting code & software for further information.

Data

Policy information about availability of data

All manuscripts must include a data availability statement. This statement should provide the following information, where applicable:

- Accession codes, unique identifiers, or web links for publicly available datasets
- A list of figures that have associated raw data
- A description of any restrictions on data availability

All raw data and relevant processed data are deposited at GSE165306. All other data are available from authors upon reasonable request.

Field-specific reporting

Please select the one below that is the best fit for your research. If you are not sure, read the appropriate sections before making your selection.

- [x] Life sciences
- [ ] Behavioural & social sciences
- [ ] Ecological, evolutionary & environmental sciences

For a reference copy of the document with all sections, see nature.com/documents/nr-reporting-summary-flat.pdf

Life sciences study design

All studies must disclose on these points even when the disclosure is negative.

Sample size

We performed bulk RNA-seq profiling on 71 samples across age (1m, 4m & 6m), gender (Female & Male), genotype (wild-type and 5xFAD) and plaque-phagocytosis profile (XO4+ and XO4-) with at least 3 biological replicates per sample group. We performed scRNA-seq profiling on 893 microglia isolated from 6m or 24m wild type or 5xFAD mice (6m WT = 243 cells, 24m WT = 121 cells, 6m 5xFAD XO4- = 95 cells, 6m 5xFAD XO4 + = 434 cells). PSD95 internalisation within microglia cell in human frontal cortex sections was demonstrated using samples collected from n=9 AD patients and n=8 cognitively normal individuals. All other experiments were carried out in at least 3 biological replicates. As results and findings observed were supporting of one another, suggest that our sample size was appropriate.

Data exclusions

One of five bulk RNA seq replicates was removed due to technical problems that resulted in a low correlation with other samples, also median of the gene expression for this sample is zero.
One of ten human AD patients was excluded for PSD95 analysis within microglia, as no microglia were detected by the IMARIS algorithm.

Replication

Experiments were reproduced at least in 3 biological replicates, unless stated otherwise. Please refer to figure legends and methods for details. All replications in these experiments were successful but the ones indicated in Data exclusion.

Randomization

Mice were allocated for experiment according to genotype, gender and age.

Blinding

Experimenters were blinded to the genotype during immunohistological processing of tissue samples and during image acquisition and analysis. Otherwise, the investigators were not blinded during data collection and analysis as we did not consider blinding required in these types of experiments.

Reporting for specific materials, systems and methods

We require information from authors about some types of materials, experimental systems and methods used in many studies. Here, indicate whether each material, system or method listed is relevant to your study. If you are not sure if a list item applies to your research, read the appropriate section before selecting a response.

Materials & experimental systems

n/a	Involved in the study	
[ ]	[x]	Antibodies
[ ]	[x]	Eukaryotic cell lines
[x]	[ ]	Palaeontology
[ ]	[x]	Animals and other organisms
[ ]	[x]	Human research participants
[x]	[ ]	Clinical data

Methods

n/a	Involved in the study	
[x]	[ ]	ChIP-seq
[ ]	[x]	Flow cytometry
[x]	[ ]	MRI-based neuroimaging

Antibodies

Antibodies used

CD11b-BV650, Biolegend, 141723, M1/70, B241224
CD45-BV786, BD Biosciences, 564225, 30-F11, 7068552
CX3CR1-FITC, Biolegend, 149019, SA011F11, B229602
CD11a-PE-Cy7, BD Biosciences, 558191, 2D7, 7159640

	TREM2-APC, R&D Systems, FAB17291N, 237920, AADS0515091<br>CD33-PE, eBioscience, 12-0331-82, 9A11-CD33, 4308505<br>CD115-BV711, Biolegend, 135515, AFS98, B229223<br>CD11b-PE, Miltenyi Biotec, 130-098-087, M1/70.15.11.5, 5170823203<br>rabbit anti-Iba-1, Wako, 019-19741, WDH4173<br>mouse anti-PSD95, Merck Millipore, MAB1596, 6G6-1C9, 2967851<br>mouse anti-6E10, Biolegend, 803001, 6E10, B225309<br>goat anti-PSD95, Abcam, ab12093, GR317630-3<br>HIF-1 alpha Antibody, Novus Biologicals, NB100-479, AP-3<br>Alexa Fluor 488 goat anti-rabbit IgG (H+L), Life Technologies, A11008, 1705869<br>Alexa Fluor 635 goat anti-mouse IgG (H+L), Life Technologies, A31575, 1402215<br>Alexa Fluor 568 donkey anti-mouse IgG (H+L), Life Technologies, A10037, 762708<br>Alexa Fluor 647 donkey anti-goat IgG (H+L), Life Technologies, A21447, 1917928<br>Alexa Fluor 647 donkey anti-rabbit IgG (H+L), Life Technologies, A31573, 2181018<br>Pacific Blue goat anti-rabbit IgG (H+L), Life Technologies, P10994, 38885A
Validation	Antibodies obtained from the commercial source were validated by the company; detailed validation analysis and relevant literatures are provided on the company website for the products used in this study. Some antibodies were validated in previously published study as indicated in methods or the relevant literature was cited.<br><br>CD11b-BV650 (https://www.biolegend.com/en-us/search-results/brilliant-violet-650-anti-mouse-cd206-mmr-antibody-8842)<br>CD45-BV786 (https://www.bdbiosciences.com/us/reagents/research/antibodies-buffers/immunology-reagents/anti-mouse-antibodies/cell-surface-antigens/bv786-rat-anti-mouse-cd45-30-f11/p/564225)<br>CX3CR1-FITC (https://www.biolegend.com/en-us/products/fitc-anti-mouse-cx3cr1-antibody-11878?GroupID=GROUP20)<br>CD11a-PE-Cy7 (https://www.bdbiosciences.com/us/applications/research/t-cell-immunology/regulatory-t-cells/surface-markers/mouse/pe-cy7-rat-anti-mouse-cd11a-2d7/p/558191)<br>TREM2-APC (https://www.rndsystems.com/products/human-mouse-trem2-alexa-fluor-700-conjugated-antibody-237920_fab17291n)<br>CD33-PE (https://www.thermofisher.com/antibody/product/CD33-Antibody-clone-9A11-CD33-Monoclonal/12-0331-82)<br>CD115-BV711 (https://www.biolegend.com/ja-jp/products/brilliant-violet-711-anti-mouse-cd115-csf-1r-antibody-9030)<br>CD11b-PE (replacement product: #130-113-797; https://www.miltenyibiotec.com/SG-en/products/cd11b-antibody-anti-human-mouse-m1-70-15-11-5.html#pe:30-tests-in-60-ul)<br>rabbit anti-Iba-1 (https://labchem-wako.fujifilm.com/us/product/detail/W01W0101-1974.html)<br>mouse anti-PSD95 (https://www.merckmillipore.com/SG/en/product/Anti-Post-Synaptic-Density-Protein-95-Antibody-clone-6G6-1C9,MM_NF-MAB1596)<br>mouse anti-6E10 (https://www.biolegend.com/en-us/products/purified-anti-beta-amyloid-1-16-antibody-11228)<br>goat anti-PSD95 (https://www.abcam.com/psd95-antibody-ab12093.html)<br>HIF-1 alpha Antibody (https://www.novusbio.com/products/hif-1-alpha-antibody_nb100-479)

Eukaryotic cell lines

Policy information about cell lines

Cell line source(s)	1. H9 CX3CR1-TdTomato cells (Grubman et al 2020 Stem Cell Reports), 2. BV2 cells (gift from Prof Peter Crack, University of Melbourne) , 3. Human Microglia Primary (Celprogen, 37089-01)
Authentication	1. PCR, Southern Blotting, Teratoma (Grubman et al 2020 Stem Cell Reports), 3. Human Microglia Primary cells were authenticated by Celprogen as stated in the data sheet; the Human Microglia Primary was derived from CNS-Cortex (brain).
Mycoplasma contamination	Tested: negative
Commonly misidentified lines<br>(See ICLAC register)	no commonly misidentified cell lines used in this study.

Animals and other organisms

Policy information about studies involving animals; ARRIVE guidelines recommended for reporting animal research

Laboratory animals	Mus Musculus (Male and Female)<br>Heterozygous 5xFAD transgenic mice (B6SJL hybrid background) - 1, 4 and 6 months old<br>Wild type - 1, 4, 6, 9, 12 and 24 months old<br>Animals were housed at Monash Animal Research Platform (MARP) under specific pathogen-free conditions (temperature: 18-24°C; humidity: 40-70%) in a day-night controlled light cycle, provided with food and water ad libitum.
Wild animals	Study did not involve wild animals.
Field-collected samples	Study did not involve samples collected from the field.

Ethics oversight	Protocols and use of animals were undertaken with the approval of the Monash University Animal Welfare Committee following the 2004 Australian Code of Practice for the Care and Use of Animals for Scientific Purposes and the Victorian Prevention of Cruelty to Animals Act and Regulations legislation.<br>Ethics Approval: Monash Animal Research Platform MARP/2016/112

Note that full information on the approval of the study protocol must also be provided in the manuscript.

Human research participants

Policy information about studies involving human research participants

Population characteristics	Patients were selected for immunofluorescence analysis: 10 AD and 10 control patients based on pathological analysis of Amyloid and Braak clinical staging. Patients were female and age matched, with mean age 72.6 (control) and 73.4 (AD), range 59-82.7 years.
Recruitment	Patients were chosen from brain donors to the Victorian Brain Bank. Pathological assessment of AD performed by Prof Catriona McLean
Ethics oversight	Ethics Approval: MUHREC 2016-0554

Note that full information on the approval of the study protocol must also be provided in the manuscript.

Flow Cytometry

Plots

Confirm that:

- [x] The axis labels state the marker and fluorochrome used (e.g. CD4-FITC).
- [x] The axis scales are clearly visible. Include numbers along axes only for bottom left plot of group (a 'group' is an analysis of identical markers).
- [x] All plots are contour plots with outliers or pseudocolor plots.
- [x] A numerical value for number of cells or percentage (with statistics) is provided.

Methodology

Sample preparation	Samples were prepared as described in Methods section.<br>Microglia isolation: Whole mouse brains, excluding brain stem and olfactory bulbs, were dissected into cerebellum and non-cerebellum regions for microglia isolation. Single cell suspensions were prepared from brain tissues by mechanical dissociation using mesh of decreasing sizes from 250microns to 70 microns and enriched for microglia by Percoll density gradient separation. Microglia-enriched cell population isolated from the 37%-70% Percoll interphase was diluted in ice-cold PBS and recovered by cold centrfugation. The cell pellet was then stained with antibodies to microglial cell surface markers.<br>OSHC microglia isolation: OSHC was mechanical dissociated using 70 µm mesh and enriched for microglia by density gradient centrifugation in 30 % (v/v) isotonic Percoll. Cell pellet was then stained with antibodies to microglial cell surface markers.<br>In vitro cells: Cells were dissociated as appropriate with Accutase, Trypsin or EDTA. Cell pellet was stained with the appropriate antibodies and resuspended in FACS buffer for FACS analysis or FACS sort.
Instrument	BD FACSAria™ III cell sorter & BD LSRFortessa™ X-20 Flow Cytometer
Software	Collection: BD FACSDiva Software (v8.0.1),<br>Analysis: FlowJo (Version 10.4.2), Cytobank (Version 6.1.2; Cytobank, Inc.).
Cell population abundance	Gated using appropriate negative controls, >95% of the live single cells prepared for sorting from brain samples were identified as microglia. Additional cells were sorted from each sample for post-sort to check for purity of sorted cell population. FACS isolation of replenished and endogenous microglia was carried out using appropriate negative controls and sample purity was determined by post-sort check. mCherry+ BV2 cells were FACS sorted for AF488-fAβ using appropriate negative controls. Abundance of cell populations of interest (synaptosome, E. coli or fAβ internalization by ex-vivo mouse microglia, synaptosome internalization by mCherry and shRNA Hif1a mCherry BV2 cells, synaptosome internalization by iMGLs, HIF1A expression in HIF1A- or ELF3- overexpressing primary human microglia) was determined using appropriate negative control during FACS analyses.
Gating strategy	Gating strategy for microglia isolated from brain samples is as presented in Supplementary Fig. 1a. Microglia were defined as live/propidium iodide -, CD11b+, CD45lo, CX3CR1+ single cells and were negative for CD11a. The Methoxy-XO4+ population gate was set using Methoxy-XO4-injected wild-type animals.<br>Endogenous and exogenous microglia (CD11b+, CD45lo) from OSHCs are defined as CFSE- and CFSE+, respectively.<br>Samples of ex-vivo mouse microglia, BV2 cells, iMGLs and primary human microglia were gated accordingly to respective gene marker and fluorescent label of synaptosome, E. coli or fAβ as described, refer to methods for details. Gating strategies for FACS sorted cells are shown in Supplementary figures 1a, 9a and 10d.

- [x] Tick this box to confirm that a figure exemplifying the gating strategy is provided in the Supplementary Information.
